# Direct Human-AI Comparison in the Animal-AI Environment

**DOI:** 10.3389/fpsyg.2022.711821

**Published:** 2022-05-24

**Authors:** Konstantinos Voudouris, Matthew Crosby, Benjamin Beyret, José Hernández-Orallo, Murray Shanahan, Marta Halina, Lucy G. Cheke

**Affiliations:** ^1^Leverhulme Centre for the Future of Intelligence, University of Cambridge, Cambridge, United Kingdom; ^2^Department of Psychology, University of Cambridge, Cambridge, United Kingdom, Cambridge, United Kingdom; ^3^Department of Computing, Imperial College London, London, United Kingdom; ^4^Valencian Research Institute for Artificial Intelligence (VRAIN), Universitat Politècnica de València, València, Spain; ^5^Department of History and Philosophy of Science, University of Cambridge, Cambridge, United Kingdom

**Keywords:** human-AI comparison, artificial intelligence, AI benchmarks, comparative cognition, out-of-distribution testing, Animal-AI Olympics, cognitive AI

## Abstract

Artificial Intelligence is making rapid and remarkable progress in the development of more sophisticated and powerful systems. However, the acknowledgement of several problems with modern machine learning approaches has prompted a shift in AI benchmarking away from task-oriented testing (such as Chess and Go) towards *ability*-oriented testing, in which AI systems are tested on their capacity to solve certain *kinds* of novel problems. The Animal-AI Environment is one such benchmark which aims to apply the ability-oriented testing used in comparative psychology to AI systems. Here, we present the first direct human-AI comparison in the Animal-AI Environment, using children aged 6–10 (*n* = 52). We found that children of all ages were significantly better than a sample of 30 AIs across most of the tests we examined, as well as performing significantly better than the two top-scoring AIs, “ironbar” and “Trrrrr,” from the Animal-AI Olympics Competition 2019. While children and AIs performed similarly on basic navigational tasks, AIs performed significantly worse in more complex cognitive tests, including detour tasks, spatial elimination tasks, and object permanence tasks, indicating that AIs lack several cognitive abilities that children aged 6–10 possess. Both children and AIs performed poorly on tool-use tasks, suggesting that these tests are challenging for both biological and non-biological machines.

## Introduction

In recent years, humans have been outperformed by AIs in several domains previously thought to be too difficult for current or near-future systems. These include Chess (Campbell et al., 2002; Silver et al., 2017), Jeopardy! (e.g., Ferrucci et al., 2013), Go (Silver et al., 2016, 2017), Shogi (Silver et al., 2017), Dota 2 (OpenAI, 2018), StarCraft II (DeepMind, 2019), and multiplayer no-limit Texas hold’em poker (Brown and Sandholm, 2019). These results are remarkable, but they remain limited in several ways, due to the nature of the deep neural networks (DNNs) that underpin them. Douglas Heaven reports that many developers, engineers, and scientists see DNNs as “fundamentally brittle” (Heaven, 2019). They are exceptional at solving test problems taken from the same distribution as their training data but perform poorly and unpredictably when faced with slightly different problems (Crosby, 2020; Geirhos et al., 2020; Hernández-Orallo, 2020). Geirhos et al. (2020) argue that DNNs suffer from several issues that give rise to this phenomenon, wherein they appear to be *intelligently* solving tasks when they are actually only finessing solutions *via* a number of “shortcuts” (the so-called *Clever Hans Effect of AI*; Sebeok and Rosenthal, 1981; Sturm, 2014; Hernández-Orallo, 2019, 2020). Geirhos et al. argue that an effective measure against this issue is to test AIs on *out-of-distribution* (o.o.d.) *data*, since, so long as testing uses data drawn from the same distribution as the training data (i.i.d., independent and identically distributed), it is impossible to distinguish between an agent that genuinely knows *how to solve* a problem and one that is using problem-irrelevant shortcuts to maximize reward (also Dickinson, 2012 for a perspective from animal psychology). Cases of adversarial attacks on image classifiers illustrate this: Dong et al. (2018) report how a DNN can be fooled into classifying the Alps as a dog and a puffer fish as a crab, by adding human-imperceptible noise to the original image. What such cases suggest is that certain AIs are overfitted to their training data or are using task-irrelevant correlations as ‘shortcuts’ to solve problems (Crosby, 2020). The move from i.i.d. to o.o.d. testing is gaining popularity (e.g., Jia and Liang, 2017; Agrawal et al., 2018; Akula et al., 2020; Teney et al., 2020 for an overview), but not only because it promotes robustness in AI systems. It also marks a shift toward more *ability*-oriented AI benchmarking, as opposed to *task*-oriented benchmarking as in the cases of Chess and Go (Hernández-Orallo, 2017a,b). While many developers of, say, image classifiers would claim that they are creating systems with “the ability to classify objects,” the use of i.i.d. testing runs the risk of creating systems that simply “solve the task of classifying the training distribution,” ultimately by whatever means necessary. Using o.o.d. testing enables researchers to have grounds to say that they are testing for the presence of abilities. However, determining which o.o.d. data to select is problematic—it would not do to require a facial recognition system to start classifying dogs and cats in a test setting, if its only input were faces. In this way, then, the o.o.d. data need to be *meaningfully related* to the training data but related in *unpredictable* and/or highly abstract ways (Crosby, 2020). A new testing platform, the Animal-AI Environment, is a means of generating training, validation, and test sets for use in AI development, wherein we can generate out-of-distribution, relevant, and abstract ability-oriented testing.

The Animal-AI Environment, first implemented in the Animal-AI Olympics 2019 (Beyret et al., 2019; Crosby et al., 2020), offers a means by which to test AIs on a range of tasks used in comparative cognition. This provides a way to meaningfully define the training-test distribution relation, through testing of cognitively defined *abilities*. Comparative cognition as a field of research has long been asking whether animals can solve out-of-distribution, abstract tasks. A major thrust of the research literature is to develop experimental paradigms that can distinguish between “associative learning” using surface features (i.e., “shortcuts”) and “cognitive ability” (see Buckner, 2011). To do this, researchers must develop ways for eliciting behaviours from animals that could not plausibly arise from “mere” stimulus–response association, thus enabling them to adjudicate between the predictions of “associative,” “shortcut” theories and “cognitive,” ability-based theories of animal learning. Regardless of whether this research practice is fruitful *in principle* (e.g., Papineau and Heyes, 2006; Meketa, 2014), it has resulted in a rich catalogue of experimental designs that can be used to at least minimally distinguish between “shortcut” approaches to problem-solving and ability-based, intentional approaches. The Animal-AI Environment offers a simple way of reconstructing homologues of these designs for use in AI testing, enabling, *for the first time*, AI developers to test their systems on these ability-oriented tasks. The Animal-AI Olympics contained 300 comparative cognition tasks using the inventory of objects available in the Animal-AI Environment. These were kept as a secret, and therefore difficult to predict, test set for investigating the *abilities* of the AIs submitted to the competition. The marriage of comparative cognition and AI benchmarking thus enables o.o.d. testing, but since the environment’s inventory of objects is strictly defined, selection of o.o.d. test sets is transparent. There is a further benefit to this partnership, in that we know that these tasks are relevant for comparison with biological systems since real animals have been demonstrated to solve them. Therefore, while the precise nature of these abilities is hotly contested, it is manifestly true that these abilities are possessed by (some) animals and are thus of potential evolutionary value. The AAI Environment offers a rich resource for the next stage in AI benchmarking.

In this paper, we present the first direct AI-human comparison using the AAI Environment, contrasting human children aged 6–10 with the top AI agents submitted to the 2019 Animal-AI Olympics Competition. We were interested in how children compared to AIs on these common cognitive tasks, and whether this differed by the developmental stage of the child (using age as a proxy). First, we detail five reasons why human-AI comparison using this environment is beneficial to both AI and comparative cognition research. Second, we introduce the Animal-AI Environment, Testbed, and Olympics in full. Third, we present the first direct AI-human comparison using this framework. While AI-human comparisons have been done before in other environments (e.g., Insa-Cabrera et al., 2011; Schrimpf et al., 2018, 2020; Kosoy et al., 2020), this study is novel in its treatment of AI as objects for cognitive science research. Both AIs and human participants were presented with completely new, out-of-distribution cognitive problems to solve, in which neither have received extensive problem-specific training. In this respect, the experimental paradigm developed here permits a more direct comparison of the general problem-solving capabilities of each type of agent. It extends beyond simplified same/different claims in human-AI comparison by drawing on methodologies from (comparative) psychology. We used robust and transparent null hypothesis significance testing methods to compare performance between age groups and AIs. We also applied clustering and dimensionality reduction to examine whether patterns of performance across the tasks were informative as to whether an agent was drawn from a human or an AI population. It is hoped that human performance in this study will constitute a benchmark for general problem-solving skill in future AI algorithms, thus offering the first sophisticated case of the new generation of AI benchmark.

## The Animal-AI Environment, Testbed, and Olympics

The Animal-AI Environment is the platform in which the Animal-AI Testbed was developed. The testbed offers a means by which to test AIs on a range of tasks used in comparative cognition to examine the problem-solving capabilities of a range of species, from crows, chimpanzees, and humans, to octopuses, goldfish, and dolphins. The Animal-AI Olympics is a competition, first run in 2019, that asks AI developers to build systems that could solve the tasks in this testbed. In this paper, we compare the AI systems submitted to the Animal-AI Olympics 2019 competition with human children aged 6–10 as a demonstration of how the Animal-AI Testbed can be leveraged for direct human-AI comparison, laying the foundation for future direct comparisons using both human and non-human animals.

The Animal-AI (AAI) environment uses Unity (Juliani et al., 2020) to generate the virtual analogues of comparative cognition tasks used in the AAI testbed, including trap-tube and string-pulling/hook tasks, radial-arm- and Y- mazes, and Thorndike escape boxes. The AI agents submitted to the 2019 AAI Olympics Competition were predominantly Deep Reinforcement Learning systems. A principal aim of the Animal-AI Olympics Competition is to determine whether AI agents could solve problems that animals have been shown to solve in the lab. The general set-up of the testbed and the Olympics aims to imitate elements of animal cognition tasks both in terms of the type of problem and the context in which it is presented. Developers of AI agents were provided with the test arena and the inventory of objects that could populate it, and then instructed that their agents would be tested on problems that animals can solve, without being provided, during the development process, with any of these problems. They were informed, however, that the tasks would only ever include items and objects that were available for interaction during training. Within the AAI Testbed, while the participating agent is not fully “embodied” in the sense of having a functional body (with limbs, multiple senses, etc), it is contextualized in that it acts within a 3D visual arena (where information is provided in the form of pixels – i.e., visually) whose properties and possible interactions are dictated by a physics engine. Egocentric velocity is provided as a vector for the AIs and as a visual display for human players. This provides the speed of the agent in the three dimensions, and acts as a proxy for proprioception. There are objects that can be moved, and objects that cannot, there are movements that can be performed (e.g., ascending a ramp), and those that cannot (e.g., flying, jumping). In each task, the objective for the agent is to maximize its “points” within a time-limit. Points are gained or lost through contact with rewards of differing size and significance, and punishments of differing severity. Points start at 0 and decrease linearly with each timestep. This creates time pressure, and therefore motivation for fast and decisive action. Obtaining a yellow “fruit” increases points, obtaining a green “fruit” also increases the points and is the ultimate objective, ending the level. Moving onto an orange area of floor accelerates the rate of point decrement over time, and so should be avoided unless necessary. Touching red “fruit” or moving onto a red area of floor (“Lava”) ends the level with failure (“death”). All fruit can be either stationary, or in motion through all three dimensions, and they can be of various sizes, indicating different reward values (but always spherical). There are a range of objects that might help or hinder achieving the goal, including opaque grey and translucent immovable barriers, blue platforms, pink ramps, pushable blocks, and pushable “cardboard” boxes. More information is available in the Supplementary Material.^1^ The 2019 AAI competition used 3 variations of each of 300 individual tasks, almost all adapted directly from the animal cognition literature, ranging from simple object retrieval to complex tool-use tasks.^2^ The AAI competition also provides plenty of raw data, in the form of a points score on each task and positional data at each time step, which can be variously processed for the purposes of the specific hypothesis testing.

## Why Compare Artificial Intelligence to Humans?

The Animal-AI Environment is clearly well-placed in serving as an o.o.d. AI benchmark (see Chollet, 2019; Crosby, 2020; Hernández-Orallo, 2020). However, what benefit does human testing offer? We present five reasons why direct human-AI comparison using the AAI Environment is critical:

### Tests the Assumption That Tasks Used in the AAI Olympics Are Solvable by Humans

Beyret et al. (2019) and Crosby et al. (2020) argue that the tasks used in the AAI Olympics are easily solvable by humans. This is a plausible assumption to make since variants of these tasks are solvable by at least some non-human animals and human children in the lab. However, it remains an untested assumption within the specific context of the AAI Environment for o.o.d. testing. It may be the case that certain extraneous factors (e.g., reaction times, ability to interact with the environment, appearance of objects) make some or all of these tasks much harder to solve than the “real-life” physical tasks they were based on. If so, holding AIs to these standards would be unfair, constituting a case of what Buckner (2013) calls “anthropofabulation,” or the exaggeration of human capabilities. Anthropofabulation is a concern within comparative cognition research. It is often assumed that “simple” behaviors, such as bending wire to make a hook (Weir et al., 2002) are also simple for humans. However, these assumptions are rarely tested, and when they are, many of these tasks prove surprisingly non-trivial – spontaneous wire bending, for example, does not occur reliably until 8–10 years of age (Beck et al., 2011). Similarly, control conditions that assume rational behavior (for example, not avoiding a trap if it is non-functional, e.g., on top of a tube) are sometimes failed by adult humans even if the requisite understanding (things do not fall up) is demonstrably present (Silva et al., 2005). Hence, the solvability of the Animal-AI tasks, while plausibly assumed, must nevertheless be tested empirically. In this way, we may show that these tests are meaningful analogues of “real life” cognitive tasks, and within the grasp of artificial agents, if development occurs in the right areas, permitting *actionable research* (Crosby, 2020).

### Provides Direct Data of How a Biological Agent Solves Each Task

Turing (1950) suggests that instead of artificially simulating the adult mind, we should instead focus on simulating the child mind. To this end, providing data on not just whether but *how* children solve some of the tasks in the AAI Testbed offers a concrete cognitive blueprint for how engineers might develop systems capable of solving the same tasks. By analyzing which objects children attend to or which search paths they take when solving tasks, and how this differs to the way certain families of machine learning techniques perform in identical tasks, we can develop a diagnostic for progress in AI research towards “human-like” intelligence. In this way, the AAI Environment moves from being just a benchmark for measuring AI progress, to a dynamic research programme facilitating dialogue between cognitive science and AI. It may be argued that lab data already exist on how children perform in some of the tasks presented here. While these comparisons are certainly informative and important for AI progress, having humans interact with the AAI Environment directly means more sophisticated comparisons can be made beyond the gross level of success rates. When both types of agents are tested within the same environment (albeit *via* different interfaces), the playing field is levelled, permitting more accurate human-AI comparison (Firestone, 2020). Therefore, for the purposes of developing AIs that can pass the AAI Olympics, the empirical studies conducted and proposed here provide more pertinent data than can be obtained in the laboratory.

### Provides a Stepping-Stone Toward Direct Comparison With Non-human Animals

The limitations in comparing physical task data and that collected in virtual environments also apply to comparisons between AIs and non-human animals. While we have a range of data, collected from a myriad of species, on how animals perform on the physical versions of tasks, current comparisons to AI performance can only be indirect. This implementation of the AAI Environment for use with humans acts as a stepping-stone towards later implementations with non-human animals, whether *via* joystick control, touchscreen, virtual reality, or scaled real-life matched copies of the testbed arena (Crosby, 2020). These future studies will provide a rich compendium of data on how animals from a range of species solve certain types of tasks, enabling more informed cognitive modelling in AI research (e.g., Lake et al., 2017). This study provides a starting point, presenting data from human children in different developmental stages.

### Facilitates a Reciprocal Dialogue Between Cognitive Science and AI

The benefits do not just extend to AI research, but would enable a “virtuous cycle” of breakthroughs in cognitive science as well (Hassabis et al., 2017). For example, if AIs make similar errors to, say, six-year-olds, or chimpanzees, or pigeons, on some task, then an examination of the architecture and training curriculum of the AI may well shed light on why those errors appear in the biological agents. While many AI systems are “black boxes” in a certain sense, they are not opaque in the same ways as the “black boxes” of chimpanzee, pigeon, or child brains. AIs can, for example, be reset, trained on a wholly different dataset, or restructured in certain ways such that we can form hypotheses about which factors might underly these errors. Instead of rearing new individuals which can take several years and incur high financial (and potentially ethical) cost, AI systems can be simply reset, or rerun thousands of times with minimal expenditure. AI-based analyses can then feed back into the cognitive science literature, promoting further study, and testing of phenomena that may have previously gone unnoticed. New and existing models based on these observations and insights can also be “embodied” as agents in these tasks, with their ability to explain biological behaviour assessed, setting a “virtuous cycle” into motion.

### Offers a New Experimental Resource for Comparative and Developmental Psychology

Aside from AI research, the Animal-AI Environment is also a useful resource for generating ersonali experiments for use in comparative and developmental psychology labs to supplement laboratory research. Since developers are free to generate their own configurations within the environment, psychologists would be able to create “gamified” versions of their experiments that are playable online and remotely. This would permit sample sizes to be inflated and therefore statistical power to increase. Furthermore, in the pandemic and post-pandemic world where the possibility of lab and school closures remains high, the ability to conduct studies of, say, physical cognition remotely is a major benefit.

In summary, these five reasons exemplify the importance of human-AI comparison using this testbed. This study is valuable to not only AI developers interested in developing embodied and computer vision systems, but also to the AI world more generally, as well as to comparative and developmental psychologists.

## Materials and Methods

**Figure 1:**
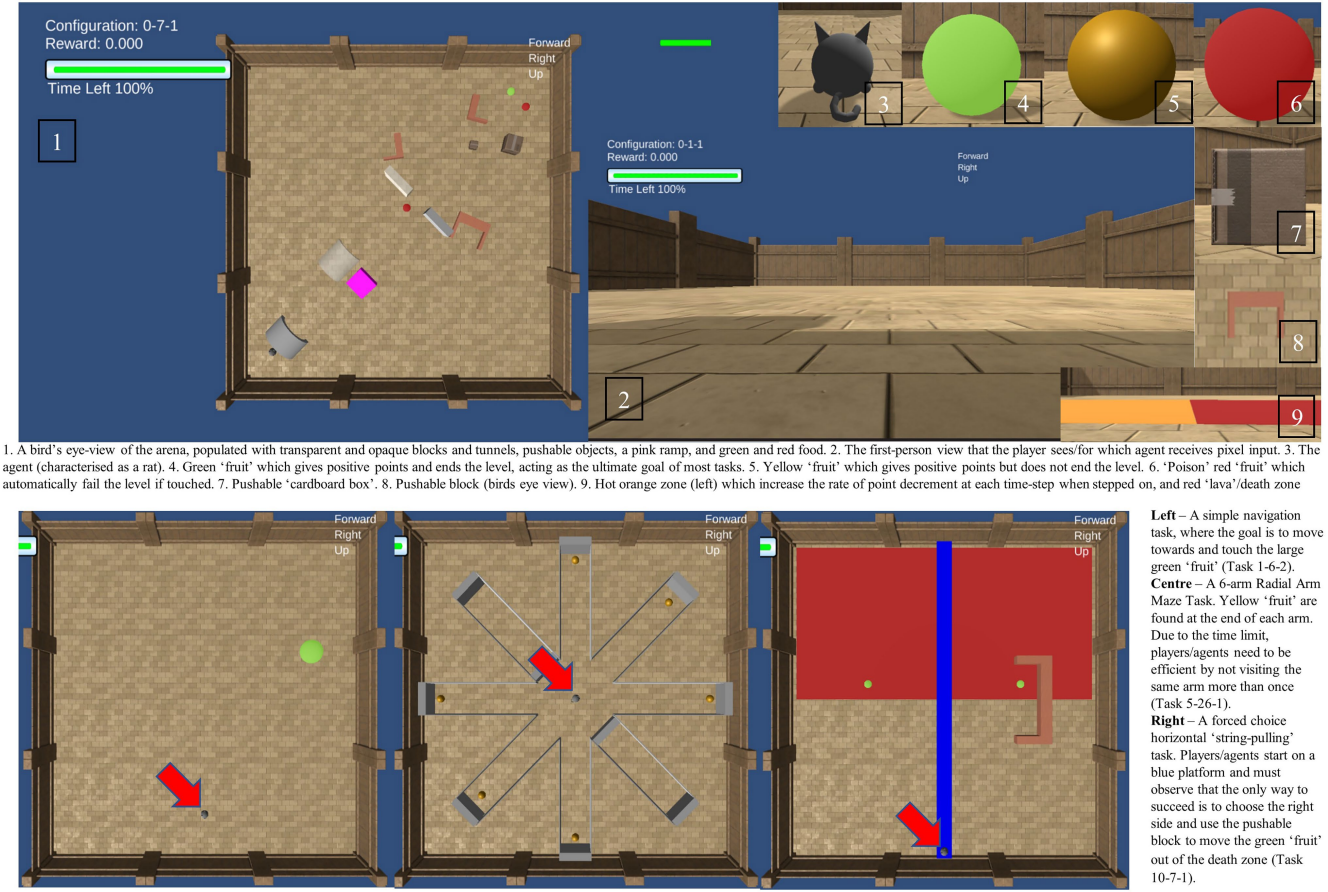
A visual description of the Animal-AI Environment and Testbed. Full details are presented in the Supplementary Material. Images of the Animal-AI Environment and Testbed are licensed under Apache License, Version 2.0 (http://www.apache.org/licenses/LICENSE-2.0).

The AAI testbed includes 300 tasks which can be broadly divided into 10 groups of increasing difficulty. They are presented in Table 1 and Figure 1. In the AAI Olympics competition, AI agents were tested on 3 variations of each of the 300 tasks. Variations included minor perturbations of objects in the arena (light, shadow, position), and minor adjustments in starting position. This was to reduce the influence of task-irrelevant biases on performance and is similar to running repeated trials on the same test subject. Pass marks, or threshold values, are part of the AAI Testbed, and are designed to signify if an agent has the ability being tested for. For example, in the 6-arm Radial Arm Maze, the pass mark is set such that the test is passed if all six food items are obtained within the time limit and failed otherwise. For the current study, 40 tasks were adapted into online computer games permitting children to interact with the environment. Four tasks from each level were randomly selected, the only constraint being that they must be easily rendered by Unity WebGL for the online study. One of the three variants of each task was randomly selected. The Animal-AI Environment and Testbed, originally written in Unity, was made into an online game using Unity WebGL (Juliani et al., 2020), enabling online access *via* the competition website using ersonalized User IDs and passwords. Full details of these tasks can be viewed in the Supplementary Material. The entire AAI Testbed can be played on the AAI website (FN3). This study was given ethical approval by the Cambridge Psychology Research Ethics Committee.

**Table 1:** Tasks grouped into 10 levels of increasing difficulty.

Level Name	Level Description	What is required of the agent?	Task Examples
L1 - Food Retrieval	Rewarding and aversive stimuli in an open arena containing no obstacles.	Basic navigation towards rewarding stimuli and away from aversive stimuli. Tests whether the agent can navigate the arena and achieve the simple goals of obtaining rewards.	This is not a tested skill within comparative cognition, as it is assumed that any creature is able to feed itself to survive.
L2 - Preferences	Rewarding and aversive stimuli arranged in forced-choice or free-choice arrangements. All stimuli can be viewed from the same position (the agent need not reorient itself to view the stimuli)	Selection of most rewarding stimuli when presented with multiple visible options. Tests whether the agent has a notion of which stimuli are the most rewarding.	Y-mazes (Ryback, 1969; Castilla, 1972; Pollard et al.,1994; Pajor et al., 2003) Delayed gratification tasks (Beran, 2002)
L3 - Static Obstacles	Rewarding stimuli are fully or partially occluded by opaque or transparent static obstacles such as walls, ramps, tunnels, or boxes.	Navigation around variable static objects to obtain rewards that may be initially out of view. Tests whether the agent can explore the arena in the search for occluded rewarding stimuli.	Detour tasks and cylinder tasks (Maclean et al., 2014; Langbein, 2018) Escape boxes (Thorndike, 1911)
L4 - Avoidance	Rewarding and aversive stimuli are arranged around aversive zones.	Navigation in an arena containing aversive zones. Tests whether the agent avoids aversive stimuli.	Y-maze variants (see L2)
L5 - Spatial Reasoning and Support	Rewarding stimuli are occluded, or not simultaneously visible from one position. They may also be supported out of reach by other static objects.	Inferences about the locations of rewarding stimuli from their absence elsewhere. Tests whether the agent can reason about space and how external objects can support each other.	T-mazes (Qin and Wheeler, 2007) Spatial elimination tasks, support tasks, and radial arm mazes (Bailey et al., 1989; Kamil et al., 1994; Hughes and Blight, 1999; Bailey et al., 2000; Lipp et al., 2001; Leplow et al., 2003)
L6 - Generalisation	A selection of tasks from previous levels, except that the colour of the walls and flooring (except orange and red zones) is varied.	The agent is required to ignore irrelevant cues about colour. Tests whether the agent is using the colour of background objects as a cue to behaviour in the arena.	This is often a feature of controls within animal cognition tasks rather than a feature of test variables, e.g., counterbalancing colour, or stimulus location.
L7 - Internal Modelling	A selection of tasks from previous levels except that visual information is blocked at periodic intervals.	The agent is required to continue navigating towards rewarding objects despite lack of visual input. Tests whether the agent behaves through step-by-step responses to pixel output or whether broader action plans are carried out.	‘Lights out’ radial arm mazes (Etienne et al., 1994)
L8 - Object Permanence and Working Memory	Rewarding stimuli pass out of view behind occluding objects.	The agent is required to navigate to rewarding stimuli by inferring where they are from their initial trajectories before they were occluded. Tests whether the agent acknowledges that objects persevere even when they move behind a barrier.	Primate Cognition Test Battery (PCTB; Herrmann et al., 2007) Object Permanence tasks (Chiandetti and Vallortigara, 2011)
L9 – Numerosity and Advanced Preferences	Preference tasks (L2) where the number of rewarding stimuli in each choice is high (3+). These are augmented with object permanence tasks (L8)	The agent is required to count the number of rewarding stimuli available and judge which option is optimal for the goal of reaching maximum points. These stimuli may pass behind occluding objects. Tests whether the agent acknowledges the number of rewarding stimuli in making preference decisions.	Numerical discrimination tasks (e.g., Kilian et al., 2003; Stancher et al., 2015)
L10 – Causal Reasoning	Rewarding stimuli are only accessible through interactions with one or more non-rewarding stimuli such as boxes and pushable blocks.	The agent is required to manipulate non-rewarding objects in the arena to facilitate them in obtaining rewarding stimuli. Tests whether the agent can reason about how objects interact causally to carry out multi-stage actions.	Trap-tube tasks (e.g., Seed et al., 2009) String-pulling/hook tasks (Hauser et al., 1999; Taylor et al., 2007; Jacobs and Osvath, 2015) Tool choice tasks (e.g., Wimpenny et al., 2009; see also Jelbert et al., 2014) Box and banana task (Köhler, 1925)

“Rewarding stimuli” refer to green/yellow “fruit. “Aversive stimuli” refer to red “fruit.” “Aversive zones” refer to red and orange areas of the arena. In the final column, the experimental paradigms that inspired each level are referenced.

### Participants

Data on AI agents were drawn from the results of the Leverhulme Centre for Future of Intelligence’s Animal-AI Olympics competition (Crosby et al., 2020). For training, the developers were provided with a “playground” which contained the testing arena which could be configured to include permutations of the possible objects. Most of the competition entries used deep reinforcement learning algorithms, some with convolutional neural network architectures augmenting the RL algorithms, along with hand-coded elements to bias learning in favor of certain perceivable competition goals. For example, the AI agent “Trrrrr,” the winner of the AAI Olympics competition, was rewarded for achieving vertical velocity, meaning it actively sought out ramps (see Crosby et al., 2020). Note that the specific computational architectures and training curricula for each agent were not submitted by the competition entrants as they were proprietary, so that information is not available at this time. 61 AIs were submitted to the competition. Full data were available for 58 of these agents. Four of those AIs were not involved in simulations of all 40 tasks, and so were excluded. A threshold for inclusion was used to remove any agents that failed in 2 out of 4 of the tasks in L1, since this eliminated agents that were unable to perform the fundamental task of navigating towards and obtaining the green reward. Agents that could not succeed in doing this were unlikely to be indicative of cutting-edge machine learning algorithms. This resulted in a sample size of 30 AI agents being involved in the final analyses.

Children were recruited *via* parents/guardians using an online sign-up form distributed on social media. We were interested in recruiting participants from a range of age groups, to begin to understand whether performance on AAI Tasks could be associated with developmental stage. Studies using computerized test batteries have shown large age-related effects (Gur et al., 2012). We wanted to understand for example, which AIs outperformed 6-year-olds but not 7-year-olds, and how this might relate to cognitive development in children. Due to potential motor difficulties with young children, we decided to use a conservative lower age of 6-years-old, and due to potentially wider levels of experience in older children, we elected an upper age of 10-years-old. Parents/guardians were invited to participate in a study investigating flexible novel problem solving in children aged 6–10; they were informed that their child would be asked to play an online game not unlike “Minecraft” and that parents/guardians would be asked to fill out a short questionnaire. They were also informed that they would be reimbursed with £5 for their and their child’s time. Parents/guardians who had signed up were then contacted with details on how to engage in the study. Partial questionnaire and game-play data were collected for 107 children. 59 of those children completed all 40 tasks. Applying the same threshold as for the AIs, a final sample size of 52 children was used in the study, eliminating 7 participants who did not pass 2 out of 4 tasks in L1. This eliminated participants who might have experienced a glitch or a browser crash at the start of the game, which happened to some, according to a post-study questionnaire. The median age of the sample was 8 (mean age = 8.096). The final dataset included: 7 six-year-olds, 10 seven-year-olds, 16 eight-year-olds, 9 nine-year-olds, and 10 ten-year-olds.

### Procedure

Parents/guardians were provided with a link to an online Qualtrics survey (Qualtrics, 2019), which they filled in with their child. Parents/guardians and child provided informed consent and then answered questions about their child’s age (6, 7, 8, 9, or 10 years old) and whether they were color-blind. They were also asked how often their children play computerized video games, what genre of games these tend to be, and what controls they use. The children were shown a two-minute video explaining the rules of the online game, before following a link to the game itself. The children were provided with some tutorial rounds within a set-up similar to the AIs’ “playground,” which they could attempt as many times as they wished. This allowed them to adapt to the controls, which involved using arrow keys on a keyboard to navigate. Participants could move forwards and backwards with the ↑↓ keys and orientate themselves in any direction with the ← → keys. The test then began, in the level ordering presented in Table 1 (see Supplementary Material for task orderings). Participants were given twice as long as the AIs to complete each task because of differences in reaction times and visual acuity (due to it being presented on computer screens and online *via* internet browsers).^3^ We did not counterbalance the order of the tasks because of concerns about the sample size in each of the counterbalanced groups.

Since the total points and pass marks varied greatly between tasks due to differences in the number of available “fruit,” total points and pass marks were converted into “accuracy” values. These values correspond to the proportion of the maximum possible points for each task achieved by each agent or required to pass each task (for pass marks). Maximum points values were defined as the maximum number of points achievable before the end of the first time-step (an impossible value to achieve, in practice). Minimum points values were corrected for rounding errors (see Supplementary Material for details). Accuracy values are reported on a scale from 0 to 1. A value of 0.5 for agent accuracy means that the agent achieved half of the possible points available for that task. A value of 0.5 for a pass mark means that an agent needs to obtain half of the maximum available points for a task to be deemed as having passed it.

### Hypotheses and Statistical Analyses

We used Neyman-Pearson Null Hypothesis Significance Testing to examine various hypotheses relating to whether human children and AIs performed differently, using the conventional significance level of 0.05. We performed parallel analyses with and without outliers (using the 1.5xIQR rule) because of the online, unsupervised nature of the study. We reasoned that perhaps very high and very low results may have arisen due to software malfunction, given that participant were taking part from home with investigators on hand to help. Therefore, we wanted to check whether removing outlier results affected interpretation. Where the statistics literature is unclear as to which test is uniformly most powerful for the data and distributions at hand, several alternative methods are used. We could, therefore, transparently determine the robustness of our results under different statistical conditions (e.g., parametric vs. non-parametric assumptions). Two comparisons are made, (i) between all 52 children and all 30 AIs (*agent contrasts*) and (ii) between each age group and AIs (*age-group contrasts*). Correlation coefficients^4^ were used to examine whether there is a difference between what the AIs and the children found difficult by determining if there was a significant correlation between performance and level: That is, how does performance vary as tasks progress from L1 to L10? This indicates the relative general problem-solving ability of humans vs. AIs; if performance tends to decrease as level complexity increases, this suggests that the agent might exhibit general abilities applicable to a wide range of problems. In contrast, no correlation might suggest that that agent has more specific abilities suited to a subset of the levels and ill-suited to the others, perhaps suggesting a lack of generality.

A Two-Way Mixed ANOVA was used to examine whether AIs and children differ in their performance in the tasks (between-subjects factor) and across the 10 levels (within-subjects factor). Accuracy was averaged across the four tasks of each level. Main effects of Level (L1-L10) and Agent (AI:Children) and interaction effects of Level*Agent were calculated. The Aligned-Rank Transform (ART; Wobbrock et al., 2011; Kay and Wobbrock, 2020) was used to facilitate a nonparametric analysis. Generalized eta-squared ($\eta_{g}^{2}$) effect sizes were reported for the parametric ANOVA, with a $\eta_{g}^{2}$ of 0.2 or above considered to be a large effect size (Lakens, 2013). To examine whether the AIs and children differ on the individual levels, Mann–Whitney U Tests were used. Vargha and Delaney’s A was used as the measure of effect size for these tests. Welch’s Two-Sample t-test (and Cohen’s d) was used as the parametric alternative. To examine how individual age-groups compare with AIs, parametric and nonparametric Two-Way Mixed ANOVAs, were run and t-ratio contrast effects calculated (Lenth et al., 2019). These ANOVAs, Mann–Whitney U, and t-tests were used to help answer the question of how well the AI models explain the behaviour of the biological comparator (human children) across a range of age groups. If we failed to reject the null hypotheses, this suggests that the AI models perform similarly to the human children. If we rejected the null hypotheses, then the AI models do not perform similarly.

An extensive exploratory analysis was then conducted. A k-medoids clustering algorithm was used to examine how the data are grouped together. Two separate clustering analyses took place. First, overall accuracy data for each participant, without information about whether they were produced by a child or an AI, acted as input for the clustering algorithm, to determine whether grouping (child vs. AI) information could be extracted. The phi coefficient and Cramer’s V were used to determine whether these clusters were significantly associated with child vs. AI information (Lüdecke, 2019) This analysis was supplemented by a clustering analysis on each group (AIs vs. children) to determine whether they individually cluster into different groups, following Kosoy et al. (2020). Post-clustering analyses, using several correlation coefficients,^5^ were conducted to determine whether any clusters in the child data correlate with age or video-game experience. Cluster quality is defined by “average silhouette width,” meaning the average distance between each data point in one cluster and one of the other clusters, and is measured from −1 to 1, with 1 indicating high clustering of the data points and − 1 indicating that data points should be classified as being in different clusters. 0 indicates that data points are on average equidistant from all clusters, suggesting a non-clustered distribution (Rousseeuw, 1987). Strong clustering is suggested by an average silhouette width of at least 0.75, medium clustering by a width of at least 0.5, and weak clustering by a width of at least 0.25 (*ibid*.).

The k-medoids analysis was supplemented with a dimensionality reduction technique called Uniform Manifold Approximation and Projection (UMAP; McInnes et al., 2020). This allowed us to check the robustness of clustering results and to visualize how the AIs and children compared across all 40 tasks. The results of clustering, UMAP, and overall performance enabled the selection of two AIs, “Trrrrr” and “ironbar,” for further analysis and discussion. Both were individually compared to children first in terms of the percentiles they were in with respect to the children’s performances. Then they were compared using one-sample Hotelling’s T^2^ test across all 40 tasks, using the *χ*^2^-distribution and the F-distribution (Signorelli et al., 2019). Hallin and Paindaveine’s (2002a,b) Multivariate Signed-Rank Test (with Tyler Angles) was run as the nonparametric alternative (Nordhausen et al., 2018). Using the F-distribution for the Hotelling’s test enabled the computation of confidence intervals adjusted for family-wise error rate, for *post hoc* comparisons on a task-by-task basis. Correlation coefficients by level and by task were also generated for “Trrrrr” and “ironbar” individually. All analyses were conducted in R (R Core Team, 2020). Further details on statistical methods and results, along with further tables, a full description of the tasks, and R Scripts can be found in the Supplementary Material.

## Results

### AI Rankings

Across the 40 tasks used in this study, the rankings of the AIs differed slightly from those in the full competition. Table 2 presents the rankings for the top 10 agents in the sample of 30. See Supplementary Material for the full rankings.

**Table 2:** Rankings of 30 AI agents involved in this study compared to ranking in AAI Olympics 2019 Competition.

AI/Team Name	Total Average Accuracy (4 decimal places, d.p.)	Ranking	AAI Olympics Rank
Ironbar	0.4896	1	2
Trrrrr	0.4881	2	1
Sirius	0.4308	3	3
ARF-RL	0.4278	4	8
Sungbinchoi	0.4198	5	6
Melflo (oltau.ai)	0.4196	6	5
DeepFox	0.4095	7	7
Juramaia	0.3910	8	10
BronzeBlood	0.3906	9	4
mmIA	0.3900	10	12

The top 3 AIs are the same as in the overall AAI Olympics Competition. However, over these 40 tasks, “ironbar” was slightly more successful than the competition winner, “Trrrrr.”

### Agent Contrasts

#### General Statistics

Across all 40 tasks, children’s scores (median = 0.6276, mean = 0.6052, SD = 0.1476) were higher than those of the AI agents (median = 0.3529, mean = 0.3412, SD = 0.0809). 0% of AIs passed Levels 3 (static obstacles), 6 (generalization), 8 (object permanence and working memory), 9 (numerosity and advanced preferences) and 10 (causal reasoning), on average, where at least some children passed these levels, on average. In fact, at least 6 children (often several more) solved every task in the 40-task testbed. The Supplementary Material includes the percentage of each sample that passed each task, not averaged by level.

**Figure 2:**
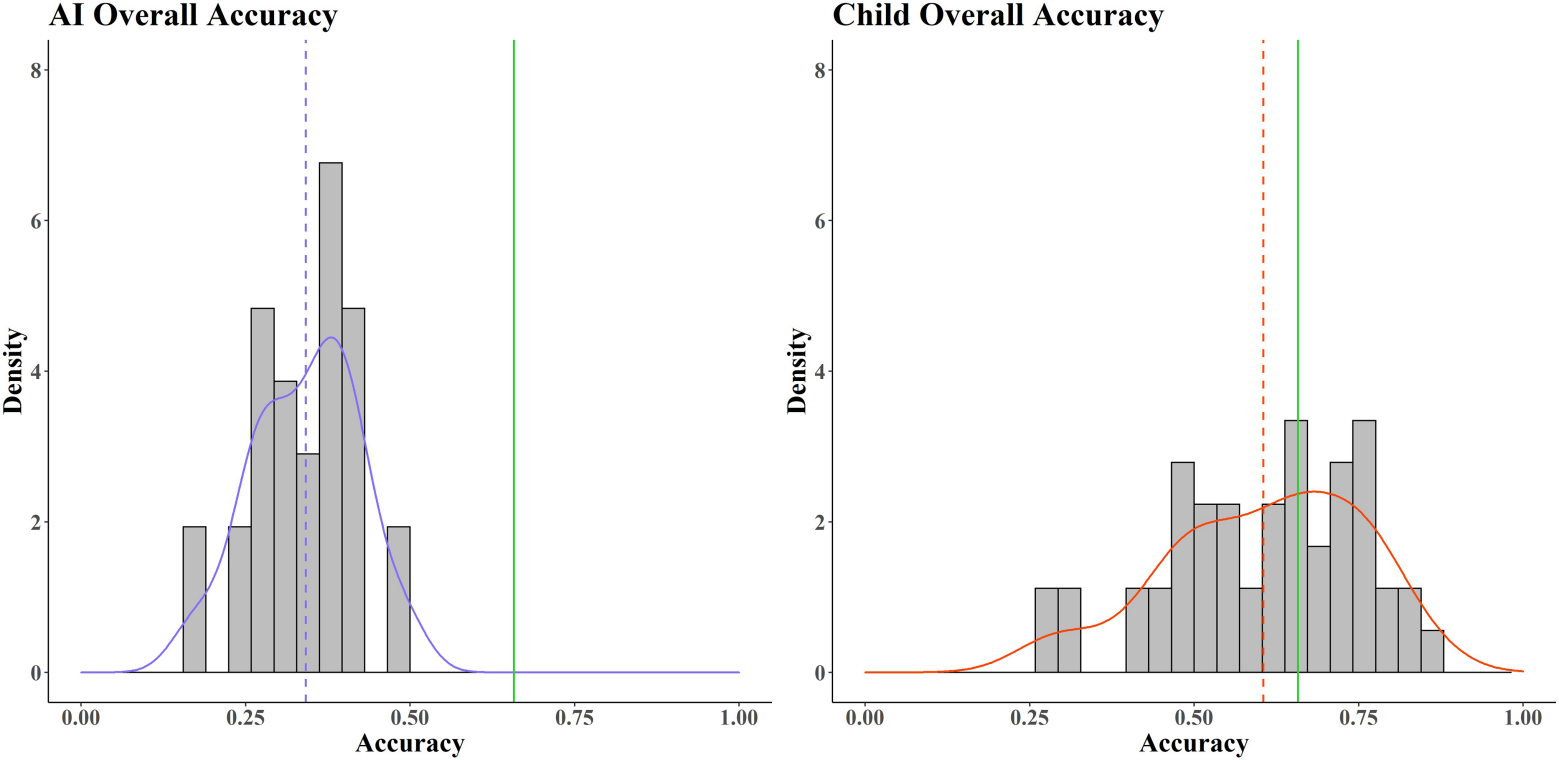
Histograms of accuracy averaged across 40 tasks. AIs (**left**, purple) and children (**right**, red). The average pass mark across the 40 tasks is shown by the green line. Red/purple solid lines show the probability densities. Red/purple dotted lines show average accuracy.

Figure 2 shows that the average score for AIs was well below the average pass mark/threshold value. The estimated probability of success (in terms of pass/fail) for an AI is very low, approaching zero. In contrast, whilst the average accuracy for the children is still below the average pass mark threshold, it is considerably closer, with a much higher probability of success for that group. This suggests that the children (the biological comparator) are generally more capable of solving this wide range of tasks than the AIs (the model). Notable also is the difference in spread, with the children showing much greater variance compared to the AIs. This might be because the children represent a more heterogenous population than the AIs (systematic variance), due to subgroup differences in age and experience with computer games, something we explore below. Alternatively, it may be because the children simply have a more variable pattern of performance (random/unexplained variance).

**Figure 3:**
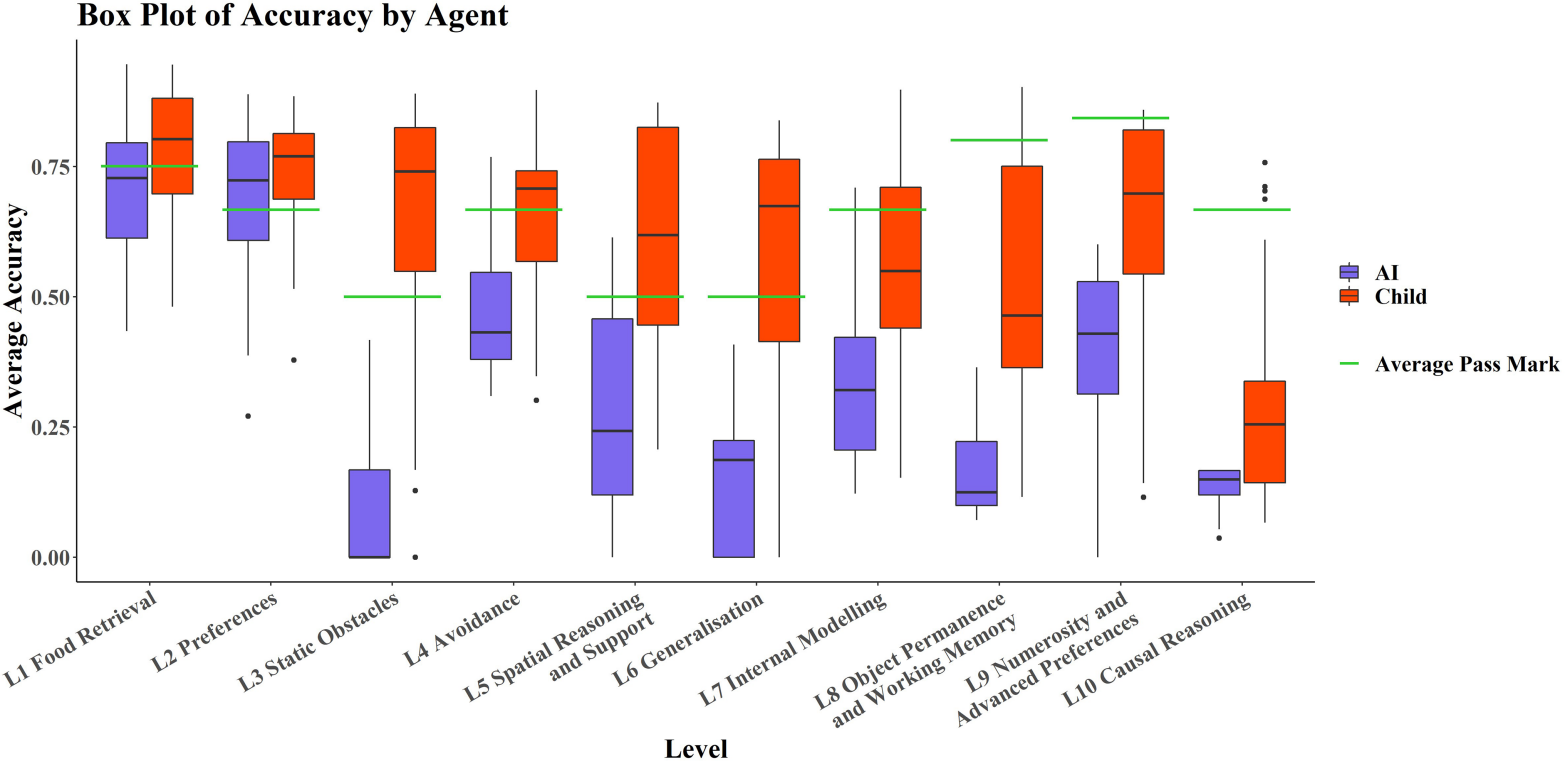
Boxplots by level and by agent. Levels are in ascending order on the x-axis, with AIs in purple (left hand boxplot of each pair) and children in red (right hand boxplot of each pair). Average pass marks for each level are shown in green.

Figure 3 shows that the AIs only perform comparably with the children in L1 and L2; these levels tested basic navigation toward rewarding stimuli in an open field and simple preference tasks, and only in Level 2 are most of the AIs successful. No significant correlation was detected between average score on each level and level number for the AIs (*r*_τ_ = −0.378, *p* = 0.1557). However, there was a significant weak correlation between individual task scores and level number (*r*_τ_ = −0.268, *p* ≈ 0.0185). The significance of these results was unaffected by method. There was a significant strongly negative correlation between average score on each level and level number for the children (*r*_τ_ = −0.8222, *p* < 0.001). There was also a significant moderate correlation between individual task scores and level number (*r*_τ_ = −0.4242, *p* ≈ 0.0002). The significance of these results was unaffected by method.

It is also noticeable in Figure 3 that AIs performed particularly poorly on Level 3 and Level 6 compared to Levels 2, 4, 5, and 7. Breaking these levels down into their respective tasks, tasks 6-11-1 and 6-12-2 are both Y-maze variants, much like 3-11-1, so poor ability in these L6 tasks may be partially explained by poor ability on this L3 task. *Post hoc* paired t-tests and Wilcoxon signed-rank tests were run to examine this. They showed that performance in 6-11-1 was not significantly different from performance on 3–11-1 [t(29) = 2.01, *p* = 0.05408], but that performance on 6-12-2 was significantly different from performance on 3–11-1 [t(29) = 2.26, *p* = 0.03146]. The significance of these results was not affected by the use of the Wilcoxon either (V = 20, *p* = 0.05917; V = 21, *p* = 0.03603, respectively). This is interesting in that the only difference between 6-11-1 and 6-12-2 was the positioning of the agent and reward relative to each other. An agent with the *ability* to solve detour tasks like these would be robust to these kinds of irrelevant changes (as children are), suggesting perhaps that the AI agents are using some “shortcuts” to solve these tasks. We also looked at performance on 6-9-1, which was a simple navigation task but in an oddly colored environment, and compared it to the analogous L1 task, 1–4-3 (without odd coloration of walls, floor, and ceiling). Performance on 6-9-1 was significantly different from performance on the analogous L1 task 1–4-3 [t(29) = 24.21, *p* < 0.0001]. The significance of this result was not affected when a Wilcoxon signed-rank test was used as a nonparametric alternative (V = 464, *p* < 0.0001). Similar reasoning can be applied here. The coloration of the walls, floor, and ceiling should be irrelevant to the task of navigating toward a reward. The fact that these irrelevant changes incur differences in performance suggests that the agents may be using task-irrelevant “shortcuts” to solve some of these tasks, unlike children whose performances are robust to these irrelevant changes.

#### Comparison of Group Means

The nonparametric ART ANOVA indicated that there was a significant main effect for the type of agent (AI vs. child; *F*(1,80) = 92.381, *p* < 0.00001) and a significant main effect for level [*F*(9, 720) = 143.434, *p* < 0.00001]. There was also a significant interaction effect between the type of agent and the level [*F*(9, 738) = 45.765, *p* < 0.00001]. These results suggest that when performance is averaged across the levels, children and AIs differ. The interaction effect suggests that the degree to which AIs and children differ is influenced by level. Running the analysis with and without outliers did not affect the significance of these results.^6^ Since the normality of the data is unclear (see Supplementary Material), a parametric variant was also run. The significance of these results is unaffected when a parametric Two-Way Mixed ANOVA^7^ was run: significant for type of agent [AI vs. child; F(1,80) = 81.512, *p* < 0.00001, $\eta_{g}^{2}$ = 0.368] and a significant main effect for level [*F*(7.36, 588.53) = 159.875, *p* < 0.00001, $\eta_{g}^{2}$ = 0.462]. These are large effect sizes. There was also a significant interaction effect between the type of agent and the level [F(7.36, 588.53) = 40.008, *p* < 0.00001, $\eta_{g}^{2}$ = 0.177]. These are medium effect sizes.

Looking at the individual levels, children and AIs did not differ significantly in L1 and L2, which tested food retrieval and preferences, respectively, but they differed significantly for all other levels (Table 3). The effect sizes were fairly small for L1 and L2, but large for the other levels, suggesting that performance was very different on these. Outliers did not affect the significance of these results. Parametric versions of these statistical methods (Welch’s t-test) did not affect the significance of these results.

**Table 3:** Mann–Whitney U-test statistics and Vargha-Delaney’s A comparing AIs and children on each level.

Level Num.	Level Name	W-statistic	Vargha-Delaney’s A
L1	Food Retrieval	560	0.349
L2	Preferences	670	0.429
L3	Static Obstacles	52^***^	0.033
L4	Avoidance	267^***^	0.171
L5	Spatial Reasoning and Support	201^***^	0.129
L6	Generalisation	153^***^	0.098
L7	Internal Modelling	303^***^	0.194
L8	Object Permanence and Working Memory	73^***^	0.047
L9	Numerosity and Advanced Preferences	219^***^	0.140
L10	Causal Reasoning	395^**^	0.253

N_AI_ = 30, N_children_ = 52. Bonferroni correction applied to significance levels. *p < 0.005, **p < 0.001, ***p < 0.0001.

### Age-Group Contrasts

#### General Statistics

Across all 40 tasks, all age groups scored more highly on average than AIs and showed greater variance (Table 4).

**Table 4:** Measures of central tendency and deviation by age group/agent type.

Age Group/Agent	Mean	Median	Standard Deviation
6 (N = 7)	0.5823	0.5993	0.1355
7 (*N* = 10)	0.6081	0.6295	0.1330
8 (*N* = 16)	0.5823	0.5993	0.1355
9 (*N* = 9)	0.6539	0.6843	0.1548
10 (*N* = 10)	0.6081	0.6294	0.1330
AI (*N* = 30)	0.3412	0.3529	0.0809

**Figure 4:**
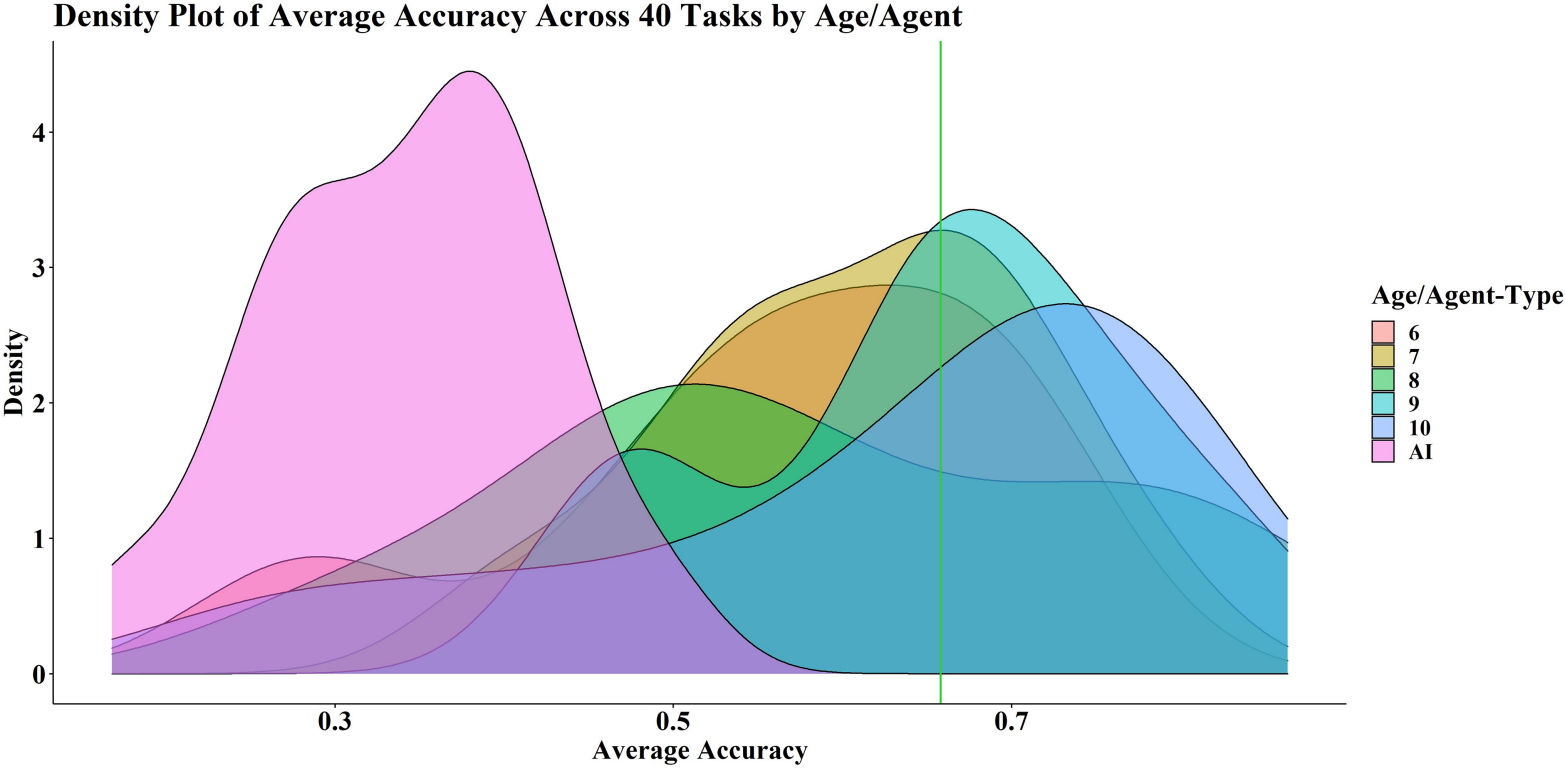
Density of plot of average score across 40 tasks, by age/agent type. The green line shows the average pass mark across 40 levels.

Figure 4 shows the probability density distribution of the average accuracy for each age group and the AIs. The age groups are grouped together distinct from the AIs. Ages 7, 9 and 10 appear to be mostly successful in that the probability density peak is on or above the average pass mark across the 40 tasks, which serves as a rough indicator of good performance. The probability density peaks for ages 6 and 8 are below the average pass mark, but the tails of the distributions are far into the successful zone.

Performance on a level-by-level basis by six- and ten-year-olds is significantly strongly correlated with level complexity, whereas there is no such significant correlation for the remaining age groups and AIs (Table 5). However, performance on a task-by-task basis by all age groups is significantly correlated with level complexity, but not so for AIs. The significance of these results (at *α* = 0.05) is unaffected by method.

**Table 5:** Kendall’s Tau by age group/agent type, with Bonferroni correction.

Age Group/Agent Type	*r*_τ_ (level average accuracy)	*r*_τ_ (task accuracy)
6	−0.7778^*^	−0.4777^***^
7	−0.6889(*p* = 0.0056)	−0.4030^**^
8	−0.7778^*^	−0.4377^**^
9	−0.6000 (*p* = 0.0157)	−0.3950^**^
10	−0.6889 (*p* = 0.0056)	−0.3710^*^
AI	−0.3778 (*p* = 0.1557)	−0.2684 (*p* ≈ 0.0185)

^*^p < 0.005, ^**^p < 0.001, ^***^p < 0.0001.

**Figure 5:**
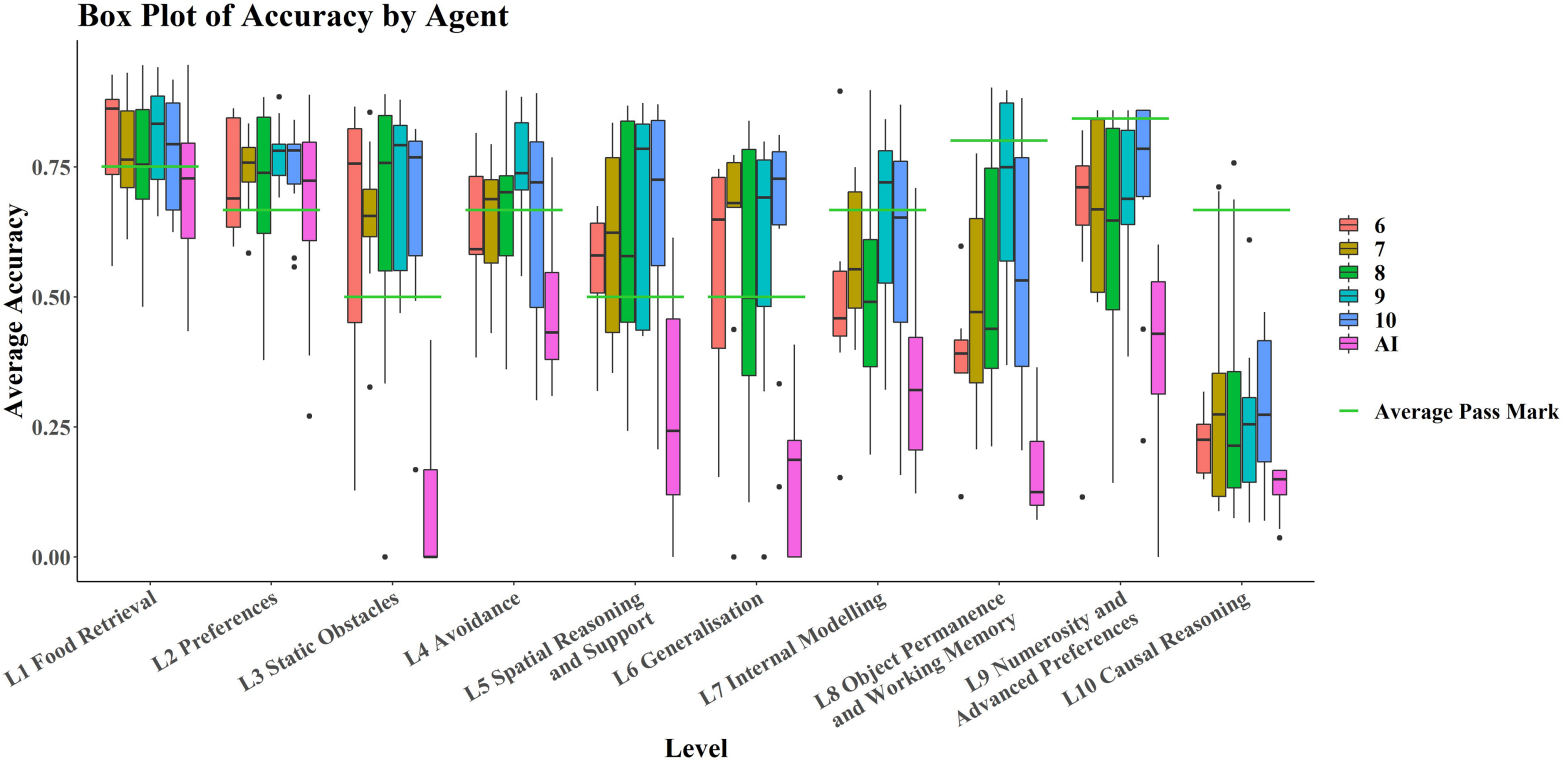
Boxplots of average accuracy on each level, by age/agent type. The left hand 5 boxplots for each level are the age groups 6–10 respectively, with the rightmost boxplot being the AI group. The green bars show the average pass mark for each level.

Age groups perform similarly on each task, performing comparable with the AIs in L1 and L2 (Figure 5). While the AIs perform considerably worse in later levels, there is no clear pattern emerging for how the different age groups perform with respect to one another.

#### Comparison of Group Means

Using the nonparametric ART ANOVA, we detected a significant main effect of age group/AI [*F*(5, 76) = 18.702, *p* < 0.001], and a significant main effect for level [*F*(9, 684) = 120.838, *p* < 0.001]. There was also a significant interaction effect between age group/AI and level [*F*(45, 684) = 10.017, *p* < 0.001]. The ANOVA was repeated with outliers removed from each age/agent type category. Removing the outliers did not affect the significance of these results.^8^ The significance of these results is unaffected when a parametric Two-Way Mixed ANOVA was run: significant for age/agent type [*F*(5,76) =16.71, *p* < 0.00001, $\eta_{g}^{2}$ = 0.387] and a significant main effect for level [*F*(7.42, 564.24) = 103.83, *p* < 0.00001, $\eta_{g}^{2}$ = 0.368]. There was also a significant interaction effect between age/agent type and the level [*F*(37.12, 564.24) = 8.886, *p* < 0.00001, $\eta_{g}^{2}$ = 0.199]. The effect sizes for all three effects are large.

Contrast effects for age/agent type, averaged over all levels are shown in Table 6. Age groups do not differ significantly from one another, but they each differ significantly from AIs. The significance of these was not affected when t-ratio was calculated on non-transformed data (the equivalent parametric tests), except for a significant effect between nine-year olds and six-year olds, and nine-year olds and eight-year olds (Figure 5).

**Table 6:** T-ratio for pairwise comparisons (contrast effects) between age groups/AIs on Aligned Rank data. All DFs 76.

Age/AI	6	7	8	9	10
7	−0.730 (−0.399)				
8	−0.349 (−0.125)	0.613 (0.247)			
9	−1.446 (−0.809)	−0.804 (−0.410)	−1.479 (−0.684)		
10	−1.053 (−0.576)	−0.356 (−0.177)	−1.008 (−0.451)	0.457 (0.233)	
AI	−4.401^***^(2.050)	6.045^***^ (2.449)	6.332^***^ (2.175)	6.779^***^ (2.859)	6.481^***^ (2.626)

Values of *p* adjusted by Tukey Method. Effect sizes (Cohen’s d) shown in brackets. ^*^p < 0.05, ^**^p < 0.01, ^***^p < 0.001.

### Exploratory Data Analysis

#### K-Medoids Clustering

Accuracy data from all 40 tasks were used in an exploratory cluster analysis, specifically k-medoids clustering. Average silhouette width was used as a metric for quality of clustering. The children weakly cluster into two groups, rather than four age groups (optimal average silhouette width: (*k* = 2) =0.2924). These clusters are significantly associated with experience playing joystick-controlled video games (*φ* = 0.5261 *p* < 0.001), meaning that children who frequently played joystick-controlled games were more likely to be in cluster 2. The clusters are also significantly associated with experience playing keyboard-based games (*φ* = 0.0468, *p* < 0.05), but this does not survive Bonferroni correction for multiple comparisons. All other associations were non-significant (see Supplementary Material for all phi statistics and a description of analysis). The AIs cluster very weakly into 2, 3 or 4 clusters (average silhouette width (*k* = 2) = 0.143; (*k* = 3) = 0.134; (*k* = 4) = 0.136). This clustering is so weak as to suggest that the AIs can be broadly treated as members of a single distribution, considering the magnitudes of accuracy and not their relative proportions. Overall, the k-medoids algorithm optimally clusters the entire dataset into two weakly clustered groups (average silhouette width (*k* = 2) = 0.356). The output of the clustering analysis suggests that there is one AI agent that clusters with the children, namely “ironbar,” which came second in the Animal-AI Olympics Competition and won the prize for the most biologically inspired entry (sponsored by The Whole Brain Architecture Initiative).^9^ However, the fact that “ironbar” clusters with children is perhaps explainable by the fact that this agent was the most successful AI across these 40 tasks (see Table 2). All other AI agents are clustered together, along with ten children, representing all age groups (two 6-year-olds, one 7-year-old, four 8-year-olds, one 9-year-olds, two 10-year-olds). The AI vs. children distinction is significantly and strongly associated with the cluster 1 vs. cluster 2 distinction (*φ* = 0.7469, *p* < 0.001), suggesting that children are highly likely to be clustered into cluster 1 and AIs are highly likely to be clustered into cluster 2. The significance of this result was unaffected when age-data was used (Cramer’s V = 0.7553, *p* < 0.001).

#### Dimensionality Reduction: Uniform Manifold Approximation and Projection

**Figure 6:**
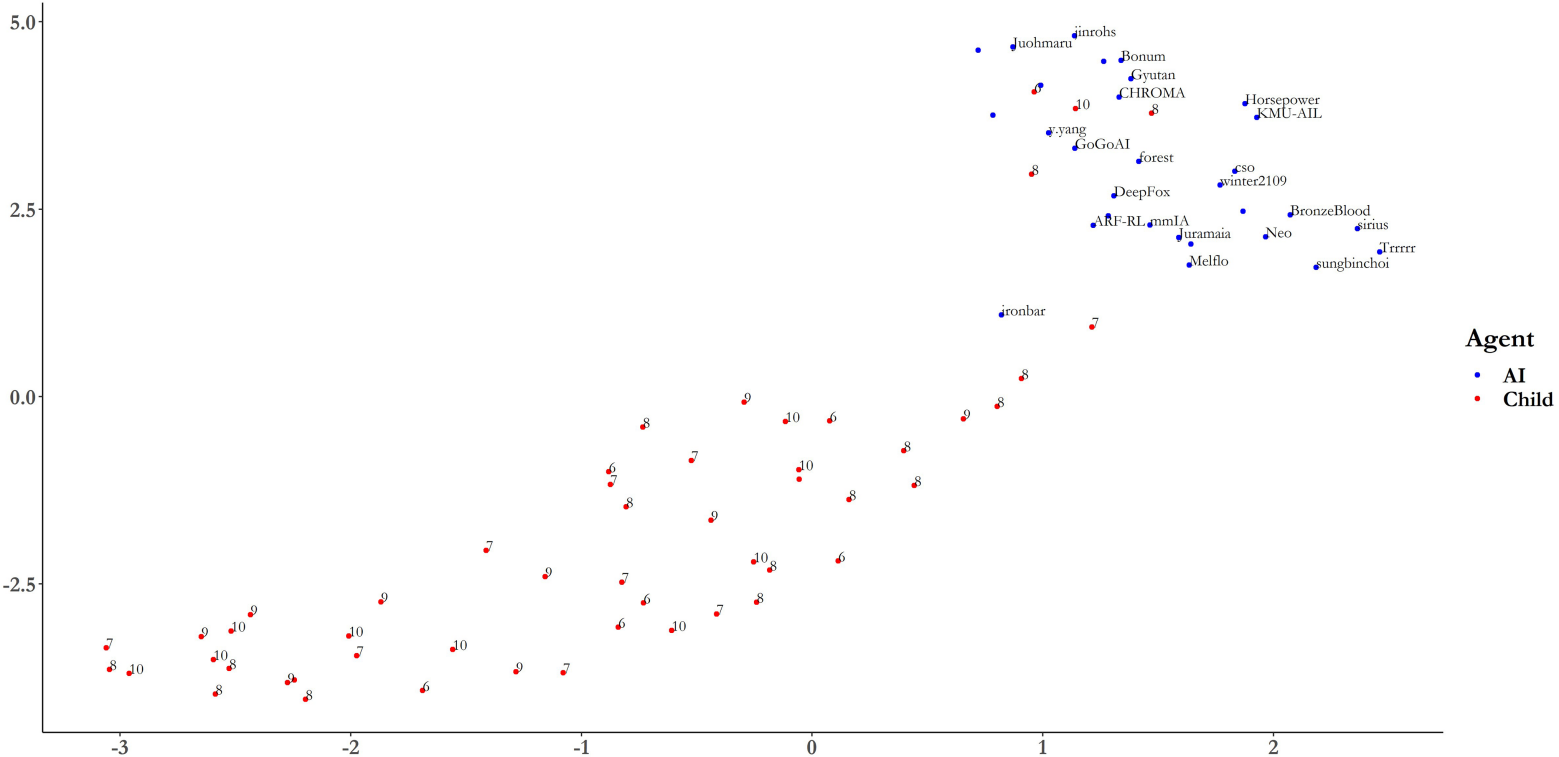
UMAP projection onto 2-dimensions using default values of N = 15 and min-dist = 0.1. The labels for AIs correspond to the algorithm name. Age labels are included for children. See the RShinyDash app provided in the Supplementary Material for different parameter settings.

Uniform manifold approximation and projection was then used as a more robust method to visualize the clustering of AIs and children. UMAP takes four hyperparameters, of which two are important for visualizing the global and local structure of the dataset: number of nearest neighbors, n, controls for the number of neighboring points considered in the local metric, with larger values preserving global structure at the loss of local structure; and minimum distance (min-dist) which determines how closely points can be grouped in low-dimensional representations, with smaller values assisting in the visualization of global structure at the loss of local structure (McInnes et al., 2020; pp. 22–3). Figure 6 shows a 2D mapping of the 40-dimensional dataset for both children (red) and AIs (blue). With the default parameters of *n* = 15 and min-dist = 0.1, the dataset clusters into two defined groups. Indeed, applying the same k-medoids clustering algorithm as above, we find a much higher average silhouette width for *k* = 2 clusters, of 0.6612. It is reasonable to assume that this marked improvement in strength of the clustering is due to the removal of strong multicollinearity problems by the UMAP dimensionality reduction. “Ironbar” no longer clusters with the children, but there are 4 children that clearly cluster with the AIs. It is particularly noticeable how the top two scoring AIs, “ironbar” and “Trrrrr” are quite differently distributed. “Ironbar” is much closer to the child cluster than “Trrrrr,” despite there being only a 0.2% difference in overall accuracy (see Table 2). This suggests that these agents may have a different pattern of performance which has a highly similar outcome in terms of gross success, with ‘ironbar’s pattern of performance aligning more closely to children than ‘Trrrrr’s. This is the subject of the next section. The UMAP dimensionality reduction can be viewed in the associated RShiny Dashboard App provided in the Supplementary Material, where the parameters of nearest neighbor and minimum distance can be toggled for examination of the local and global structure of the dataset. Having determined earlier that the ages of the children are not related with performance, these data have been omitted from the following analyses.

### Comparing Children to the Top Two AIs

This exploratory analysis has highlighted that whilst the AIs and children differ significantly at the group level, there is evidence to suggest that “ironbar” is perhaps the “most similar” in its behavior to the children, given its proximity on the UMAP manifold. Although absolute distances on this output cannot be diagnostic of actual similarities in higher-dimensional vector space, there is merit in a more precise comparison between some of the AIs and the children. “Ironbar” and “Trrrrr” differ in their overall score by less than 0.2% (Table 1), with both agents being in the 22^nd^ percentile of child performance. Yet “ironbar” appears to be “more like” the children than “Trrrrr” does. This might suggest that “ironbar” shows a similar pattern of performance to the children where “Trrrrr” does not, despite a similar outcome in overall accuracy. Table 7 presents the percentile each agent is in with respect to children’s performances, by level. Both agents are in very different percentiles in all levels except L9, supporting the conclusion that their patterns of performance are distinct.

**Table 7:** Percentile for “ironbar” and “Trrrrr” with respect to children’s performances.

Level	Percentile	
	Ironbar	Trrrrr
Overall	22nd	22nd
1	97th	46th
2	38th	72nd
3	12th	4th
4	79th	75th
5	33rd	48th
6	1st	18th
7	62nd	74th
8	25th	0th
9	11th	11th
10	32nd	21st

Both agents showed the exact same correlation coefficient relating level average accuracy and level,^10^ namely a significant moderately negative correlation (*r*_τ_ = −0.5111, *p* = 0.0466), in contrast to all 30 AIs as a sample. “Trrrrr” showed a significant modest correlation between task accuracy and level number (*r*_τ_ = −0.3347, *p* ≈ 0.0034), as did “ironbar” (*r*_τ_ = −0.3418, *p* ≈ 0.0030). The significance of these results was not affected when Spearman’s Rho was used. Pearson’s PMCC failed to reject the null hypothesis for “Trrrrr” on the task score/level number correlation. It rejected the null hypothesis for “ironbar” in this case. Recall that the children showed a significant strongly negative correlation between average level accuracy and level complexity (*r*_τ_ = −0.8222, *p* < 0.001), and a significant moderate correlation between task accuracy and level number, too (*r*_τ_ = −0.4243, *p* ≈ 0.0002). These results suggest that both “ironbar” and “Trrrrr” resemble children in that they are sensitive to level complexity as we defined it *a priori*.

One-sample Hotelling’s T^2^ using the χ^2^-distribution indicated that there was a significant difference between “ironbar” and the children [T^2^(40) = 22,242, *p* < 0.0001] and a significant difference between “Trrrrr” and the children [T^2^(40) = 40,254, *p* < 0.0001]. Hotelling’s T^2^ was also conducted using the F-distribution, and once again there was a significant difference between “ironbar” and the children [T^2^(40, 14) = 129.1, *p* < 0.0001], and between “Trrrrr” and the children [T^2^(40, 14) = 258.49, *p* < 0.0001]. Hallin and Paindaveine’s (2002a,b) Signed-Rank Location Test was used as a non-parametric alternative to Hotelling’s T^2^. The Tyler Angles Rank, Sign, and van der Waerden tests with approximated *p*-values showed non-significant differences between the two agents [Trrrrr: Q.W(40) = 48.715, *p* ≈ 0.1624, Q.S(40) = 51.316, *p* ≈ 0.1084; Q.N(40) = 50.848, *p* ≈ 0.1168; Ironbar: Q.W(40) = 48.955, *p* ≈ 0.1567, Q.S(40) = 51.393, *p* ≈ 0.1070; Q.N(40) = 50.941, *p* ≈ 0.1151]. However, the equivalent tests with bootstrapped p-values (permutations = 1,000), indicated significant differences, as with Hotelling’s [Trrrrr: Q.W(40) = 48.715, *p* = 0.004, Q.S(40) = 51.316, *p* < 0.0001; Q.N(40) = 50.848, *p* < 0.0001; ironbar: Q.W(40) = 48.955, *p* = 0.001, Q.S(40) = 51.393, *p* < 0.0001; Q.N(40) = 50.941, *p* < 0.001]. The significance of the difference between these agents can, therefore, be cautiously taken to be acceptable, since three out of four of the analyses from the analytic multiverse showed significance. To compare these agents to children on a task-by-task basis, we can use confidence intervals. However, this requires us to assume normality in the child dataset, which is not obviously the case (see Supplementary Material). While MANOVAs are robust to normality violations (e.g., Finch 2005), these data must nevertheless be interpreted with caution.

**Figure 7:**
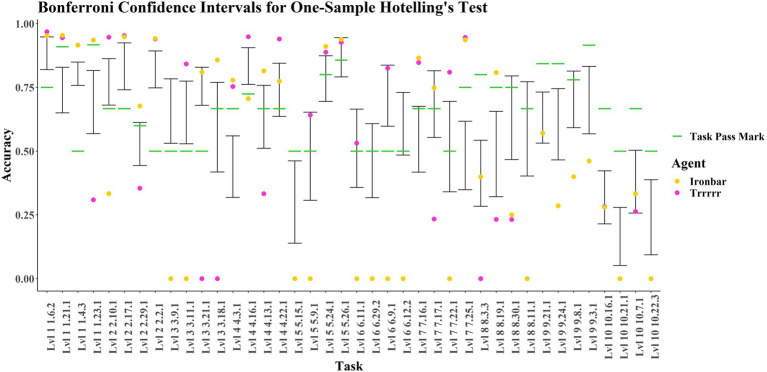
Bonferroni confidence intervals for children’s data at alpha = 0.05 with ‘ironbar’ and ‘Trrrrr’ results and pass marks overlayed.

Figure 7 presents the confidence intervals defined by the children’s performances on each task, with the results for “ironbar” and “Trrrrr” overlaid. Both these agents outperformed the average child on the simple navigation tasks in level 1 (1-21-1, 1-4-3, 1-6-2). However, “ironbar” performs considerably better than the average child, who performs considerably better than “Trrrrr,” on the more complex navigational level 1 task (1-23-1) in which 10 yellow ‘fruit’ must be obtained whilst avoiding 10 red ‘fruit’. In level 2, both agents outperform the average child on the forced- and free-choice Y-mazes (2-17-1 and 2-2-1 respectively). Task 2–10-1 was a forced choice between a red “fruit” on the left and a green “fruit” on the right. The average child was successful, with “Trrrrr” performing considerably better, and “ironbar” considerably worse. The reverse is true for the delayed gratification level 2 task (2-29-1), in which one green then two yellow “fruit” fall off a ramp and maximum points are obtained if the agent waits to obtain the yellow “fruit” before obtaining the green “fruit.” The average child performed poorly, as did “Trrrrr,” whereas “ironbar” was successful. Level 3 showed some differing results for each agent. Both agents performed worse than the average child on Task 3-9-1, forced choice detour around a transparent barrier. In contrast, “Trrrrr,” like the average child, performed well on the transparent inverted Y-maze of Task 3-11-1 where “ironbar” performed poorly. “Ironbar” performed successfully on tasks containing ramps (3-21-1 and 3-18-1) where “Trrrrr” was not successful. In level 4, which tested an ability to reason about and navigate around red “lava” and “hot zones,” at least one of the agents performed well on each task, but performance was highly inconsistent. In task 4-3-1, involving “zigzag” navigation around two “lava” zones, both agents were successful, performing considerably better than the average child, who performed worse. In task 4-16-1, involving cost–benefit analysis (choice between a large reward reached over a hot zone and a small reward directly accessible), “Trrrrr” and the average child were successful, where “ironbar” performed poorly. In task 4-13-1, a free choice “lava” T-maze, “ironbar” was successful where “Trrrrr” was not and the average child straddled the pass mark. In task 4-22-1, involving navigation across a “bridge” to avoid red “lava,” both agents were successful, where the average child overlapped with the pass mark. In level 5, both agents were successful in tasks 5-24-1 and 5-26-1, the 4- and 6-arm Radial Arm Mazes, where the average child straddled the pass mark. “Trrrrr” performed successfully in 5-9-1, a forced choice spatial elimination task (where the reward is not initially visible, but there is only one possible occluder), where “ironbar” performed poorly, and neither the agents nor the average child performed well in task 5-15-1, involving pushing a box to knock a green “fruit” off a post. In Level 6, testing generalization across varied surface features (e.g., differently colored walls), “ironbar” performed poorly on all tasks. “Trrrrr” performed successfully and similar to the average child in tasks 6-11-1 and 6-9-1, which were an inverted Y-maze variant using a ‘fence’ rather than the previously seen opaque block, and color-switched simple navigation task, respectively. “Trrrrr” performed very poorly on tasks 6-29-2 and 6-12-2, where the average child overlapped with the pass mark. These tasks were an escape maze with differently colored walls, and an inverted Y-maze with a “fence,” respectively. Comparing 6-11-1 and 6-12-2, it is interesting that “Trrrrr” performed well on the former but not the latter, when the only difference is the relative positioning of agent and goal. In 6-11-1 (good performance), the “fruit” was inside the Y-shape with the agent facing the apex. In 6-12-2, the agent was inside the Y-shape, and the goal was at the apex. In Level 7, in which visual input was removed at periodic intervals (‘lights out’), both agents were successful and outperformed the average child in tasks 7-16-1 and 7-25-1, which involved navigation around a small area of red “lava” and the obtaining of three yellow “fruit,” respectively. “Ironbar” was successful in 7-17-1 where “Trrrrr” was not, and vice versa for 7-22-1; these tasks involved navigating around a large area of red “lava,” and delayed gratification, respectively. In level 8, involving four tasks testing various aspects of object permanence understanding, both agents were outperformed by the average child in tasks 8-30-1 and 8-11-1 (both involving choice between multiple occluders). In task 8-3-3 (in which two rewards are seen moving behind occluders and must both be retrieved), both agents performed poorly, but “ironbar” performed like the average child where “Trrrrr” did not. In task 8–19–1 (in which a reward rolls out of a red zone and behind an occluder), both “Trrrrr” and the average child performed poorly and were outperformed by the successful “ironbar.” In level 9, testing advanced object permanence and numerosity, both agents performed identically, and neither agents nor children were generally successful. In tasks 9-24-1 (3 vs. 1 occluded rewards) and 9-3-1 (forced choice, 3 vs. 6 rewards), they performed worse than the average child, who was also unsuccessful. In task 9-8-1 (involving a forced choice between 1 and 2 rewards), the case is similar except that the average child’s score overlaps with the pass mark. In task 9-21-1 (3 occluded vs. 2 visible rewards), both agents performed like the average child, unsuccessfully. In level 10, testing causal cognition, both agents and the average child performed poorly, with the agents performing like the average child on the ‘string-pulling/hook’ tasks 10-16-1 and 10-7-1, and worse than the average child in 10-21-1 and 10-22-3, which were variants of classic tool-use tasks (pushing blocks to attain out of reach rewards).

Across all these tasks, it should be noted that differences in performance between children and these two agents may be confounded with differences in reaction time and ‘embodiment’ in the AAI environment. Similarities in performance may also be confounded with the fact that only certain accuracy values are possible in certain tasks, due to the number of ‘fruit’ and the time cap available.

## Results and Discussion

### Comparison on Overall Performance

This study demonstrated how direct human-AI comparison using comparative cognition tasks could be done using the Animal-AI Testbed. Here, a sample of deep RL systems and a sample of children aged 6–10 was compared across a subset of 40 tasks from the AAI test battery. Across all 40 tasks, children performed more successfully than the AIs, and this difference was significant. Each age group also performed significantly better than the AIs, but no age group performed significantly better than any other. This suggests that the children were generally better than the AIs and that age did not affect this, indicating that the performance exhibited by the children is not necessarily a function of age-modulated psychological development. Indeed, a clustering analysis over the children’s performances suggested that variance in their success might be better explained through experience with keyboard-controlled video games. We already know that many of the experimental paradigms used in the current 40-task study have been solved by children from a range of age groups in real-life laboratory set-ups, although a detailed review of how performance on these tasks compares to similar real-life tasks is beyond the scope of this paper, which is intended as an overview. It appears that the additional demand of solving these tasks on a computer game introduced, as expected, variation based on computer-experience. A clustering analysis was also performed to examine whether the groupings of “AI” vs. “child” were inferable from the variance in the dataset. It was found that weak clusters emerge that correlate strongly with “AI” vs. “child.” Only one AI clustered with the children, “ironbar,” the agent that scored the highest in the 40 tasks used in this study, and that came second overall in the Animal-AI Olympics Competition. This agent also won the prize for being most biologically inspired. However, further analysis using a method more robust to outliers and nonlinear clustering found that AIs and children clustered strongly into two groups, and that “ironbar” did not ultimately cluster with the children, although some children did cluster with the AIs.

At the broadest level of comparison, these results suggest that all the tasks are solvable by human children (to varying degrees), whilst only a subset of the tasks (the easier ones) are solvable by at least some AIs. There was a significant main effect of level, but crucially, there was also a significant interaction effect between agent type (i.e., AI/children) and level suggesting that the children and AIs differed in the way that performance was influenced by type of task. Indeed, the children’s performances were significantly, strongly, and negatively correlated with the level number, suggesting that they found the tasks more difficult on average as they progressed through the game. In contrast, the AIs performances were not correlated with level number, suggesting no relation between performance and the pre-defined notion of task-complexity. It should be noted that this was despite the fact that, unlike the AIs, children were able to learn from level to level, and continue to grow accustomed to the game environment and controls. Overall, these results suggest that the AAI Testbed and 2019 Olympics competition constitute hard but potentially achievable benchmarks for the next generation of artificial systems. No tested AI showed problem-solving abilities comparable to those demonstrated by human children.

### Comparing Performance on Specific Cognitive Abilities

**Figure 8:**
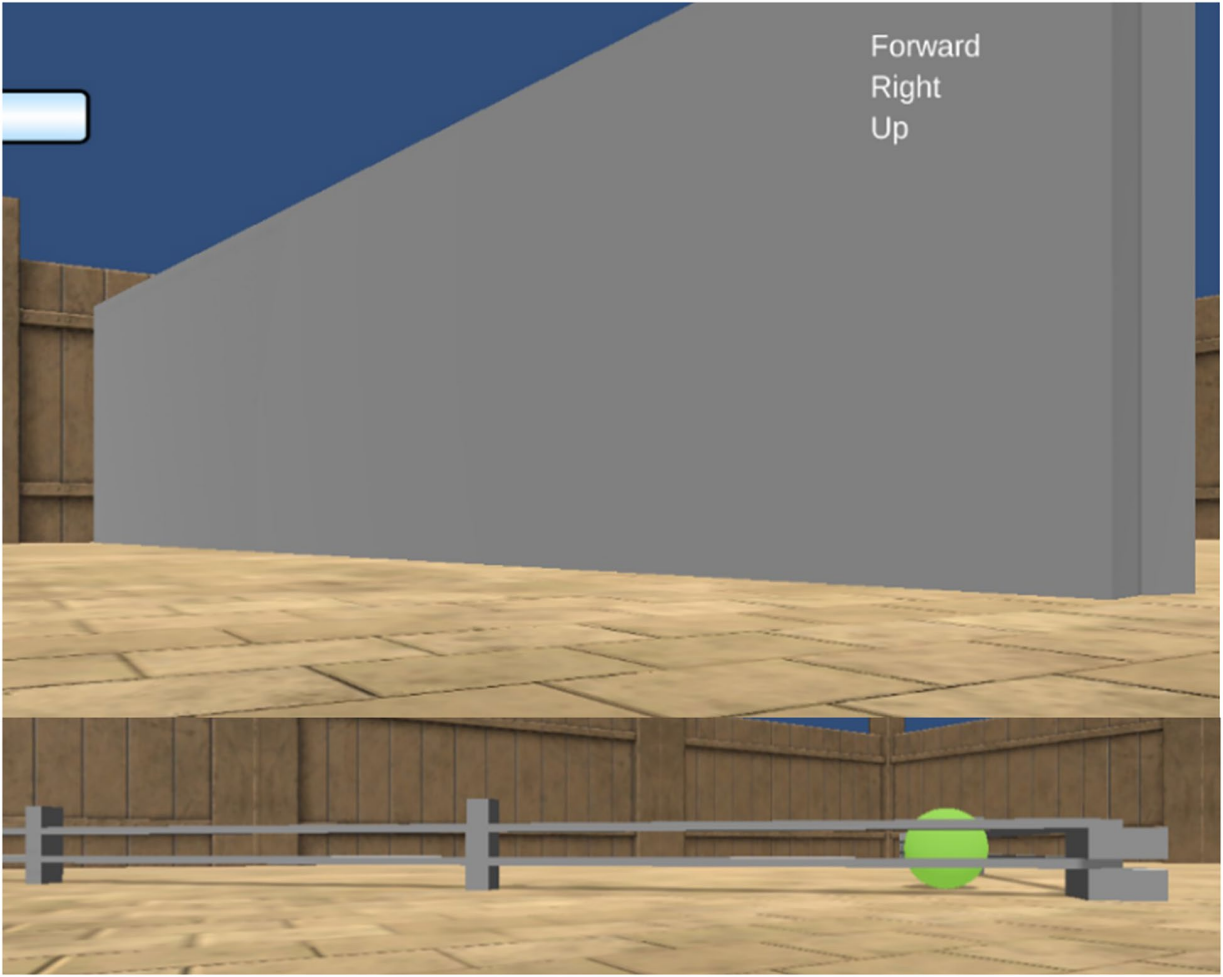
Different static obstacles in the AAI Testbed. Cuboidal blocks in L1-L5 (Top). Fence-like structures in L6 (bottom). Images of the Animal-AI Environment and Testbed are licensed under Apache License, Version 2.0 (http://www.apache.org/licenses/LICENSE-2.0).

The AIs and children performed comparably on L1 and L2, which tested food retrieval and preferences, with no significant difference between the two groups, indicating that current AI systems perform at the human level when it comes to basic navigation and food retrieval. However, in all other levels, the two groups perform significantly differently, with children always outperforming AIs on average. Noticeable are the poor performances by the AIs in L3 and L6 compared to the children (see Figure 3). L3 tested the ability to navigate around and over static objects, with two tasks (3-11-1 and 3-9-1) being detour task variants of tasks in L2 (2-10-1 and 2-17-1) and the other two tasks (3-21-1 and 3-18-1) being navigation tasks requiring movement in the third dimension up ramps (i.e., 3-D versions of L1 tasks). L6 tested generalization by using previous task configurations from L1-L5 but altering colors and structures. Concretely, 6-12-2 and 6-11-1 were detour tasks of the format used in L3 (e.g., 3-11-1) but with static obstacles of a different shape to what had been provided in the AAI training environment, namely that they were fence-shaped rather than cuboidal blocks (see Figure 8).

6-29-2 was a variant of L1 basic navigation tasks but with colorings of the walls, roof, and floor altered. A suggestion for why the AIs performed considerably worse than the children in the L6 tasks, and compared to their performances in comparable levels (e.g., L1, L2, and L4) is that the AIs used perceptual cues of color or shape to perform successfully, whereas children are capable of abstracting beyond those cues to more features pertaining to objecthood (see Dubey et al., 2018; Shanahan et al., 2020). However, *post hoc* analyses showed that performance on 6-29-2 and the analogous task 1-4-3 were significantly different, as were performances on 3-11-1 compared to 6-12-2. Performance on 6-11-1 was not significantly different from performance on 3-11-1, however. This is interesting since the only difference between 6-11-1 and 6-12-2 is the positioning of agent and reward relative to the obstacle to be navigated around. This suggests that the way we as human observers judge the difficulty of these cognitive tasks is not necessarily aligned with the difficulties that AIs face in solving them. It further demonstrates that AI ability is “brittle” and struggles to generalize to new, but similar, problems, unlike in the human case.

### Comparing Human Performance to the Two Top-Scoring AIs

The results of the clustering analysis, along with the overall accuracy value, suggested that two agents were suited to individual comparisons: “ironbar” and “Trrrrr.” Despite outperforming the other AIs, overall, these agents still performed significantly differently to children. However, a task-by-task analysis raises some interesting observations. In several of the simple search tasks, the children were outperformed by these two AIs, although this could be explained by differences in reaction/processing times. More interesting is these agents’ ability on the “lights out” navigation tasks in Level 7, in which visual input is withheld for periodic intervals. The two top agents outperformed children here, even though these tasks are more complex than some of the earlier tasks that these agents were unsuccessful at. However, this phenomenon could be explained by the AIs’ direct access to velocity data, while children would have to attend and interpret the visual display presenting this information, all while solving these tasks under time pressure. In other words, children are subject to attentional and motivational constraints that are not necessarily present for the AIs. Turning to some of the concrete comparative cognition tasks used in this study, there are some notable observations. Tasks 2-29-1 and 7-22-1 were delayed gratification tasks, in which the goal(s) rolled down a high ramp onto the floor, with the latter involving “lights out.” Forgoing immediate smaller or worse rewards for larger or better later rewards has been studied extensively in developmental psychology, most notably in the “marshmallow task” (Shoda et al., 1990; Watts et al., 2018). Delayed gratification has been used to assess self-control and future planning in both humans and nonhuman animals (Koepke et al., 2015), as well as in DRL agents (Bontrager et al., 2019). Watts et al. (2018) demonstrate that some children struggle with delayed gratification tasks, although the explanation for this, and its correlation with intelligence, is debated (Duckworth et al., 2013). Both agents showed ability here, with “ironbar” outperforming children and “Trrrrr” in 2-29-1, and “Trrrrr” outperforming children and “ironbar” in 7-22-1. While, on average, children appeared to struggle with these two tasks fairly evenly, both artificial agents show opposite ability on each task. This is perhaps suggestive that neither agent possesses an *ability* to solve delayed gratification tasks *per se*, but rather that they were able to solve these specific tasks due to some other non-cognitive factor playing a role (relating perhaps to the exact configuration of each task). Further research will investigate these findings in more detail, using the full range of delayed gratification tasks present in the AAI Testbed, as well as developing new tasks based on laboratory research. On tasks involving ramps, specifically in Level 3 (3-21-1, 3-18-1) “ironbar” showed superior performance to “Trrrrr,” despite the latter agent having been programmed to find vertical velocity rewarding (Crosby et al., 2020), suggesting that this policy is not necessarily conducive to success with ramp-based tasks. Indeed, in task 8-11-1, “Trrrrr” is observed circling around on the large ramp rather than searching for an occluded reward (which had been dropped into a ‘hole’; it should be noted that “ironbar” was equally unsuccessful on this task, however). Level 3 also contained a task involving navigating around a transparent obstacle to obtain a reward that was directly observable through and under it. Both agents performed considerably worse than children on this task and are observed crashing into the transparent block and perseverating in their forward movement to try and get to the reward that is directly in front of them. This relates to the previous suggestion that these systems are sensitive to pixel-level representations of the environment, rather than larger object-centric and affordance-centric ones. It appears both agents and children had trouble with use of pushable objects. Task 5-15-1 and tasks in Level 10 that involved these objects were, however, solved by at least some children, suggesting that the affordances of pushable objects was learnt and understood in at least some cases. Including these tasks in the testbed is, therefore, not an instance of anthropofabulation, or the inflation of human ability in comparative contexts (Buckner, 2013). However, these tasks are clearly complex, and well beyond the scope of the current state-of-the-art systems instantiated by “Trrrrr” and “ironbar.”

### Limitations

There are several limitations to this study that are worth highlighting. First, the data on children were collected online through the distribution of hyperlinks to the study page. This meant that an investigator was not present at the time of testing to determine: (a) whether the child participant was receiving help from parents/guardians/older siblings, (b) whether that child had already participated in the study using another username/email address and was, therefore, not playing the tasks for the first time, (c) whether the child fully understood the rules/controls of the game, or had been provided with adequate time to play and engage with the tutorial levels, and (d) whether technological or connection issues limited performance. For these reasons, any conclusions drawn must be tentative. Second, no data were collected on the genders, sex, or ethnicities of the participants, because these were not directly related to our research questions. This meant that we were unable to assess the representativeness of our sample. Future research should ensure that basic demographic information is collected such that results are generalizable. Third, because we do not know the details of how the AIs were coded or their training curricula (i.e., what experience they had been given), what we can infer about the AIs is limited. This means that the relatively poor performance of the AI agents studied here does not necessarily generalize to DRL agents as a class. Moreover, DRL methods are not the only approach that could be leveraged to solve the set of novel problems we analyze here. The field of computational creativity, for example, has developed approaches based on analogical reasoning and conceptual blending that could be adapted to create novel solutions to the AAI Testbed (see Veale and Cardoso, 2019, for a review). While we have not evaluated these methods ourselves, the Testbed and our dataset of human performances offers a valuable way to do so. A fourth limitation is that the scoring system is a crude metric when assessing task performance: With strict time limits, it is impossible to distinguish between slow-and-careful (but ultimately on-target) behavior and fast but pseudo-random movement, both of which would be low-scoring. Future analysis including more subtle indicators of behavior (such as path analysis) may add sufficient resolution for meaningful analysis of this type. Finally, our decision to conduct this study as a broad overview of multiple tasks, rather than a detailed investigation of specific tasks, limits the degree of inference that can be made on any given cognitive process. Future work should focus on specific cognitive processes to properly explore how current AIs are able to model performance of human children. Despite these difficulties, this study represents an important next stage in the use and implementation of the AAI Environment.

## General Discussion: The Five Reasons

This study has compared humans and AIs in the AAI Environment for the first time, using a well-grounded cognitive science approach and draws on powerful and comprehensive statistical methods and approaches, including dimensionality reduction, and clustering. This study is important, we have argued, for the following reasons:

1. Tests the assumption that the tasks used in the AAI Olympics are solvable by humans.
2. Provides direct data of how a biological agent solves each task.
3. Provides a stepping-stone toward direct comparison with non-human animals.
4. Facilitates a reciprocal dialogue between cognitive science and AI.
5. Offers a new experimental resource for comparative and developmental psychology.

The results clearly demonstrate that the tasks are solvable by human children aged 6-10, thus affirming the hypotheses made in Beyret et al. (2019) and Crosby et al. (2020; Reason 1). However, the tasks were not necessarily ‘easy’ for the human participants, especially the later levels. For example, Level 10 included two horizontal string-pulling/hook tasks (10-7-1, 10-16-1), requiring a block to be pushed to tug a green “fruit” out of a red death zone. Children performed poorly on average in both. This is notable since Redshaw (1978) showed that horizontal string-pulling tasks are solvable by human and gorilla infants at around 42-weeks and 26.5-weeks old, respectively. Since the children who participated in this study are considerably older than this, it is notable that they were unsuccessful in these tasks. This is perhaps due to the fact that the “hooks” in question are so large relative to the avatar in the game that one cannot observe the whole scene at once. Indeed, since there was no “pull” action, these tasks involved either turning away from the reward and pushing the “handle” or reversing into the handle to push the “hook” while maintaining visual access to the reward. Unlike many hook/string tasks, this does not reliably result in the reward moving incrementally closer to the agent, since the agent is either turned away or moving backwards as the reward moves (see Taylor et al., 2010; Cheke et al., 2011 for discussion on how incremental approach of reward may be important in performance in such tasks). Further research should explore contexts that more closely match the conditions of “real life” tasks, as well as exploring the relative influence of different types of information and feedback in detail.

The results presented here also provide an overview of which tasks children found hard or easy, with difficulty correlated with level number. It appears that AIs and children are sensitive to different difficulty metrics, with AIs having particular problems, compared to children, with Levels 3 and 6. This direct comparison has thus highlighted where AI research could benefit greatly from examining more deeply how children solve these particular sets of problems. Ongoing research is exploring more sophisticated comparisons using this dataset, by analyzing the paths taken by children and AI in solving these problems. This will facilitate an analysis into how children *approach* these kinds of problems, and how we might develop AIs that do the same. Future studies will also use eye-tracking or mouse-tracking software to provide information as to which objects are being attended to by human players, permitting inferences about the kinds of representations that human players use to solve tasks (see Yesiltepe et al., 2020 for the use of eye-tracking software with the spatial navigation game Sea Hero Quest). Further to this kind of study, direct comparisons using the AAI Environment will also require a more objective measure of task complexity (Hernández-Orallo, 2017a,b, 2020) in place of the somewhat arbitrary and anthropocentric level divisions used in this study, since this will allow for fairer and more objective direct comparisons (Firestone, 2020). So, while the current study only offers a broad-level direct comparison, the AAI Environment is rich and well-specified enough to facilitate sophisticated analyses that will engender cognitive modelling in AI research (Reason 2).

This study also develops the AAI environment into a *game*, with a simple player-computer interface, well-defined goals, and incremental increases in difficulty. With simple changes to the controls, the environment is ripe for use by both human and non-human animals, with this study a first implementation of how this can be done, including data on how to develop and implement tutorial levels (see Supplementary Material; Reasons 3 and 5). The capability of the AAI Environment as a dynamic research programme which facilitates dialogue between comparative psychology, cognitive science, and AI is therefore strengthened, since the AAI platform can be used for all kinds of agent comparisons, including:

- AI/human comparisons.
- AI/AI comparisons.
- Human/human comparisons.
- Non-human-animal/non-human-animal comparisons.
- Non-human-animal/human comparisons.
- Non-human-animal/AI comparisons.

The environment is therefore pregnant with possibilities for academic research across a wide range of disciplines, enabling close and collaborative dialogue (Reason 4). In summary, while the conclusions to be drawn from this study are limited for several reasons, this study serves as an important proof-of-concept demonstration of the value of the Animal-AI Environment across multiple fields.

## Conclusion

AI research has seen remarkable progress in recent years. However, many commentators and researchers have highlighted the problems with AI benchmarking and the use of independent-and-identically-distributed (i.i.d.) data, which means “shortcut” learning often appears as an inferior surrogate of true intelligence. An AI system can appear to be performing “intelligently” on a test set when that test set was drawn from the same distribution as the training data. However, when testing on some sample drawn from another distribution, it starts to breakdown in unpredictable ways. The Animal-AI Environment offers a new benchmark in which o.o.d. testing is facilitated by using “building blocks” from the training environment to create a unique permutation that is homologous with a specific cognitive task. Such an approach promotes the development of robust, general AI systems. This paper has extended the Animal-AI Environment such that it is not just a benchmark, but also the platform for an interdisciplinary research programme that brings together AI, comparative psychology, and developmental psychology. This means that the development of robust, general AI systems can run in tandem to progress in cognitive science, with both sides benefitting.

## Data Availability Statement

The datasets presented in this study can be found in online repositories. The names of the repository/repositories and accession number(s) can be found at: https://osf.io/g8u26/.

## Ethics Statement

The studies involving human participants were reviewed and approved by Cambridge Psychology Research Ethics Committee, Department of Psychology, University of Cambridge (PRE.2020.024). Written informed consent to participate in this study was provided by the participants’ legal guardian/next of kin.

## Author Contributions

LGC, MC, MH, and MS: Concept. KV and LGC: Design. MC and BB: Resources. KV, MC, and BB: Material. KV: Data collection and/or processing. KV and LGC: Analysis and/or interpretation. KV: Literature Research. KV: Writing. LGC, MC, JH-O, and MH: Critical review. All authors contributed to the article and approved the submitted version.

## Funding

This work was supported by ESRC DTP funding to KV, ESRC award reference: ES/P000738/1. Research was conducted as a project within the Kinds of Intelligence Program at the Leverhulme Centre for the Future of Intelligence, award number: G108086, and the US DARPA HR00112120007 (RECoG-AI) Grant.

## Conflict of Interest

MC, BB and MS are employed by DeepMind Technologies Limited.

The remaining authors declare that the research was conducted in the absence of any commercial or financial relationships that could be construed as a potential conflict of interest.

## Publisher’s Note

All claims expressed in this article are solely those of the authors and do not necessarily represent those of their affiliated organizations, or those of the publisher, the editors and the reviewers. Any product that may be evaluated in this article, or claim that may be made by its manufacturer, is not guaranteed or endorsed by the publisher.

## Footnotes

^1^Including details for how the reader can play the tasks presented to children in this study.

^2^These can be viewed and played here: http://animalaiolympics.com/AAI/

^3^This doubling of time limit was determined using a pilot study involving five adults, who found that they were unable to solve several tasks when provided with the same time limits as the AIs. A post-pilot survey suggested that this was because they were still learning the controls and the physics of the game. It was deemed that since human agents were not ‘embodied’ in the game environment in the same way as the AIs, giving them twice the time limit would be optimal for allowing them to think about and solve the problems, without giving them too much time such that it was not a fair comparison.

^4^Specifically Kendall’s Tau, Spearman’s Rho, and Pearson’s Product Moment Correlation Coefficient (PMCC).

^5^Phi Coefficient and Cramer’s V for binomial data. Kendall’s Tau, Spearman’s Rho, and Pearson’s PMCC for continuous data.

^6^2 outliers for AIs (N_with_ = 30; N_without_ = 28). 8 outliers for children (N_with_ = 52; N_without_ = 44).

^7^Levene’s Test shows that homogeneity of variance was present in all levels except for L3, L6, L8, and L9. Box’s M-Test showed that there was homogeneity of covariance (see Supplementary Material).

^8^When outliers are removed, sample sizes change by: 4 for 6 year olds (N_with_ = 7; N_without_ = 3); 3 for 7 year olds (N_with_ = 10; N_without_ = 7); 1 for 8 year olds (N_with_ = 16; N_without_ = 15); 1 for 9 year olds (N_with_ = 9; N_without_ = 8); 3 for ten year olds (N_with_ = 10; N_without_ = 7). AIs as earlier (N_with_ = 30; N_without_ = 28).

^9^This was awarded to the algorithm that was deemed to be most ‘biologically-plausible’ by independent judges.

^10^We have no explanation for how this identical correlation coefficient has come to be.
