# Supplementary material for: Direct Human-AI Comparison in the Animal-AI Environment
Source: Front Psychol. 2022 May 24;13:711821. doi: 10.3389/fpsyg.2022.711821 (PMC9172850; doi:10.3389/fpsyg.2022.711821)
Supplement: Supplementary file 1 [file Data_Sheet_1.pdf]

Supplementary Material

1 Supplementary tables

Table 1 - Rankings of 30 AI agents involved in this study compared to ranking in AAI Olympics 2019 Competition

AI/Team Name	Total Average Accuracy (4 decimal places, d.p.)	Ranking	AAI Olympics Rank
**ironbar**	0.4896	1	2
**Trrrrr**	0.4881	2	1
**sirius**	0.4308	3	3
**ARF-RL**	0.4278	4	8
**sungbinchoi**	0.4198	5	6
**Melflo = oltau.ai**	0.4196	6	5*
**DeepFox**	0.4095	7	7
**Juramaia**	0.3910	8	10
**BronzeBlood**	0.3906	9	4
**mmIA**	0.3900	10	12
**Neo = oltau.ai**	0.3815	11	5*
**oreleus = oltau.ai**	0.3758	12	5*
**UniboTeam**	0.3733	13	9
**CUMIN**	0.3649	14	16
**cso**	0.3538	15	11
**winter2109**	0.3519	16	14
**Gyutan**	0.3423	17	13
**forest**	0.3200	18	15
**GoGoAI**	0.3103	19	22
**y.yang**	0.3092	20	19
**KMU-AIL**	0.2958	21	25
**Horsepower**	0.2878	22	32
**Koozyt_Hiperdyne**	0.2734	23	31
**animalAI-challenge**	0.2654	24	21
**Bonum**	0.2629	25	23
**doot**	0.2602	26	24
**CHROMA**	0.2578	27	27
**Jinrohs**	0.2407	28	28
**Octopus**	0.1853	29	59
**Juohmsaru**	0.1680	30	37

* The team oltau.ai submitted 3 separate agents, Melflo, Neo, and oreleus. The team ranked 5th overall based on the performance of their best agent over the Olympics.

Table 2 - Percentages of AI (N=30) and children (N=52) who were successful on each task. 0% success rates are highlighted in purple.

Task	Short Description	Percentage of AIs Successful (2 d.p.)	Percentage of Children Successful (2 d.p.)
**1-6-2**	Navigation to 1 large green	100.00	98.08
**1-21-1**	Navigation to 10 yellows	56.67	36.54
**1-4-3**	Navigation to 1 small green	96.67	100.00
**1-23-1**	Navigation to 10 yellows, around 10 reds	6.67	34.62
**2-10-1**	Forced choice: red/green	73.33	84.62
**2-17-1**	Forced choice Y-maze: big/small green	70.00	84.62
**2-29-1**	Delayed gratification	36.67	51.92
**2-2-1**	Free choice Y-maze: green/nothing	96.67	98.08
**3-9-1**	Forced choice + transparent barrier: green/nothing	6.67	86.54
**3-11-1**	Detour around transparent blocks	20.00	86.54
**3-21-1**	Triple ramps	6.67	94.23
**3-18-1**	Single ramp plus navigation on narrow platform	3.33	69.23
**4-3-1**	Zig-zag detour around lava zones	10.00	34.62
**4-16-1**	Forced choice cost-benefit: big green + hot zone (correct) vs. small green	56.67	73.08
**4-13-1**	T-maze around lava zones	13.33	65.38
**4-22-1**	Navigation across ‘bridge’ over lava	20.00	78.85
**5-15-1**	Use of box to knock green off support	0.00	42.31
**5-9-1**	Forced choice spatial elimination	26.67	61.54
**5-24-1**	4-arm radial arm maze	23.33	77.85
**5-26-1**	6-arm radial arm maze	33.33	88.46
**6-11-1**	Detour task with fence variant	10.00	73.08
**6-29-2**	Colour switched escape task	0.00	71.15
**6-9-1**	Colour switched navigation to green	63.33	90.38
**6-12-2**	Detour task with fence variant	6.67	84.62
**7-16-1**	Detour task around lava with ‘lights out’	23.33	50.00
**7-17-1**	Detour task around lava with ‘lights out’	13.33	67.31
**7-22-1**	Delayed gratification with ‘lights out’	23.33	63.46
**7-25-1**	Navigation to 3 yellows with ‘lights out’	20.00	21.15

**8-3-3**	Free-choice object permanence	0.00	23.08
**8-19-1**	Forced choice object permanence	6.67	42.31
**8-30-1**	Forced choice object permanence	3.33	61.54
**8-11-1**	Forced choice object permanence	6.67	69.23
**9-21-1**	Forced choice numerosity + object permanence: 3 yellow/2 yellow	3.33	32.69
**9-24-1**	Forced choice numerosity + object permanence: 3 yellow/2 yellow	3.33	51.92
**9-8-1**	Forced choice numerosity: 2 yellow/1 yellow	46.67	80.77
**9-3-1**	Forced choice numerosity: 6 yellow/3 yellow	10.00	46.15
**10-16-1**	Forced choice string-pulling/hook task: broken/unbroken ‘string’/’hook’	0.00	11.54
**10-21-1**	Forced choice tool selection: cardboard box (correct)/static block	0.00	15.38
**10-7-1**	Forced choice string-pulling/hook task: ‘string’/’hook’/no tool	0.00	19.23
**10-22-3**	Box-and-banana task	0.00	36.54

Table 3 - Percentages of AI (N=30) and children (N=52) who were successful on each level. 0% success rates are highlighted in purple.

**Level Num.**	**Level Name**	**Percentage of AIs Successful (2 d.p.)**	**Percentage of Children successful (2 d.p.)**
L1	Food Retrieval	36.67	59.61
L2	Preferences	63.33	79.92
L3	Static Obstacles	0.00	80.77
L4	Avoidance	6.67	59.62
L5	Spatial Reasoning and Support	20.00	65.38
L6	Generalisation	0.00	65.38
L7	Internal Modelling	6.67	30.77
L8	Object Permanence and Working Memory	0.00	15.38
L9	Numerosity and Advanced Preferences	0.00	21.15
L10	Causal Reasoning	0.00	7.69

2 Supplementary Statistical Detail, Figures and Results

2.1 Correlation coefficients

Kendall's Tau, Spearman's Rho, and Pearson's Product Moment Correlation Coefficient (PMCC). Croux and Dehon (2010) argue that each of these are uniformly most powerful under different distributional assumptions, so all three are used within the multiverse approach.

2.2 ANOVAs

A Mixed ANOVA was used to examine whether AIs and children differ in their performance in the tasks (between-subjects factor) and across the 10 levels (within-subjects factor). Accuracy was averaged across the four tasks of each level. Normality was checked with Schapiro-Wilk Tests and evaluated visually using QQ-plots (see Appendix I). Homogeneity of Variance was tested with Levene's test, and Homogeneity of Covariance was tested with Box's M-Test. Sphericity was checked with Mauchly's test and corrected for using the Greenhouse-Geisser correction. Main effects of Level (L1-L10) and Agent (AI:Children) and interaction effects of Level*Agent were calculated. Generalised eta-squared (η_g^2) effect sizes were reported, with a η_g^2 of 0.2 or above considered to be a large effect size (Lakens, 2013). The Aligned-Rank Transform (ART; Wobbrock et al. 2011; Kay and Wobbrock, 2020) was used facilitate a non-parametric analysis. This permits a Mixed ANOVA, specifically Type III Wald F tests with Kenward-Roger degrees of freedom, to be performed whilst avoiding distributional assumptions of Normality and Sphericity. The equivalent effect size metric (η_g^2 or omega squared ω^2; Olejnik and Algina, 2003) was not available for ART transforms.

2.2.1 Normality checks

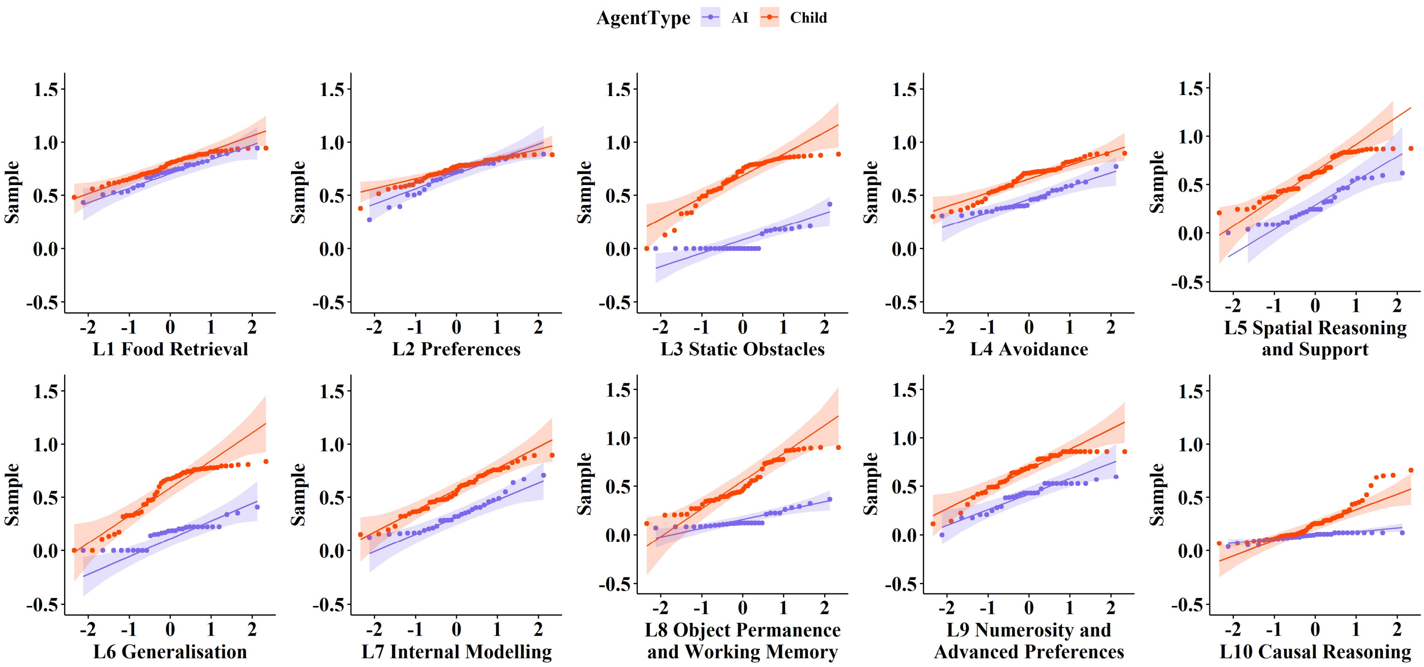

Fig. 1 - QQ-Plots by agent and by level. Top Left-Right: L1-L5. Bottom Left-Right: L6-L10. x-axis is the theoretical gaussian distribution.

Generally, the distributions for each level by agent are ambiguous in terms of Normality, with a diverse and heterogenous set of distributions. Shapiro-Wilk Tests for Normality was also performed, testing the null hypothesis that each of the distributions of average accuracy by level are normal. The results are in Table 9.

Table 4 - Shapiro-Wilk tests of Normality by level.

**Child**			**AI**	
**Level**	**Statistic**	**P-value**	**Statistic**	**P-value**
**L1**	0.952	0.0347*	0.972	0.607
**L2**	0.931	0.00506**	0.912	0.0168*
**L3**	0.863	0.0000256***	0.664	0.000000485***
**L4**	0.948	0.0245*	0.919	0.0246*
**L5**	0.921	0.00206**	0.926	0.0382*
**L6**	0.859	0.0000194***	0.867	0.00144**
**L7**	0.975	0.346	0.934	0.0618
**L8**	0.932	0.00550**	0.806	0.0000855***
**L9**	0.880	0.0000852***	0.895	0.00630**

**L10**	0.880	0.0000815***	0.812	0.000109***

***p<.05, **p<.01, ***p<.001**

Most of the distributions are significant departures from Normality. However, not all are, so multiversing with both parametric and non-parametric testing is justified. Normality checks were not multiversed for outliers.

2.2.2 Levene's Tests for Homogeneity of Variance

Levene's test tests the null hypothesis that there is homogeneity of variance between the two samples (between-subjects factor). If p<0.05, then we reject the null hypothesis, meaning that there is not homogeneity of variance. This violates the homoscedasticity assumption of ANOVAs.

Table 5 – Levene's tests of Homogeneity of Variance by level.

Level	Statistic for Agent Contrast	Statistic for Age-group contrast
**L1**	0.159	0.362
**L2**	3.22	2.01
**L3**	8.32**	1.95
**L4**	0.805	0.534
**L5**	0.475	0.842
**L6**	7.37**	1.61
**L7**	2.39	0.307
**L8**	25.2***	4.97***
**L9**	2.60	0.558
**L10**	22.5***	6.28***

***p<.05, **p<.01, ***p<.001**

Significance was affected by outliers. For agent contrasts, L2 became significant. For age group contrasts, then L2 and L6 also become significant.

2.2.3 Box's M-test for Homogeneity of Covariance

Box's M-test tests the null hypothesis that there is homogeneity of covariance between the two samples (between-subjects factor). Box's M-test is highly sensitive. Tabachnick and Fidell (2001) suggest that heterogeneity of covariance should only be acknowledged and corrected for if we have unequal sample sizes *and* p<0.001. For the mixed anova for agent-contrasts, M(1)=4.09, p=0.0431. For the mixed anova for age-contrasts, M(5)=7.57, p=0.181. When outliers were removed, the significance for the mixed anova for agent-contrasts was unaffected (M(1)=5.44, p=0.0197). However, when outliers were removed for the age-contrasts, significance was affected (M(5)=21.1, p=0.000762). Paired with unequal sample sizes, this means that the age-contrast mixed ANOVA without outliers is not robust.

2.2.4 Sphericity and Greenhouse-Geisser correction

Sphericity was automatically tested for and corrected by the Greenhouse-Geisser method in the anova function in R.

2.3 Two-sample Comparisons

To examine whether the AIs and children differ on the individual levels, Mann-Whitney U Tests were used for the data on each level, using the Bonferroni correction for multiple comparisons (i.e., using an α of 0.05/10 = 0.005 for each test). Vargha and Delaney's A was used as the measure of effect size for these tests. Welch's Two-Sample t-test (and Cohen's d) was used as the parametric alternative. VDA is given on a scale of 0 to 1, with 0.5 meaning that both groups are equal (have equal stochastic dominance on each other). Closer to 1 means that sample 1 has more stochastic dominance over sample 2, and vice versa as VDA approaches 0. To examine how individual age groups compare with AIs, parametric and non-parametric Two-Way Mixed ANOVAs, were run and t-ratio contrast effects calculated, using Kenward-Roger degrees-of freedom and the Tukey correction for multiple comparisons ([emmeans] R package, Lenth et al. 2020).

2.4 Clustering analysis

Partitioning Around Medoids algorithm (PAM; Kaufman and Rousseeuw, 1990) was used. This method finds the optimal number of clusters, k, by minimising distances between median values and the real data values. The distance metric can either be in terms of root sum-of-squares differences (Euclidean), or in terms of the sum of absolute distances (Manhattan). The Manhattan method is more robust to outliers and so will be used here. The average silhouette method is used to estimate the optimal number of clusters in a dataset, by computing PAM for various values of k (clusters) and determining the quality of those clusters in terms of the distance metric. Quality is defined by 'average silhouette width', meaning the average distance between each data point in one cluster and one of the other clusters, and is measured from -1 to 1, with 1 indicating high clustering of the data points and -1 indicating that data points should be classified as being in different clusters. 0 indicates that data points are on average equidistant from all clusters, suggesting a non-clustered distribution (Rousseeuw, 1987). Values are reported from 0 to 1 since PAM does not generate negative silhouette widths. Strong clustering is suggested by an average silhouette width of at least 0.75, medium clustering by a width of at least 0.5, and weak clustering by a width of at least 0.25 (*ibid.*).

Table 6 – Average Silhouette Width for PAM by cluster, for child dataset, AI dataset, and combined dataset.

Clusters	Children	AIs	Overall
**1**	0.00	0.00	0.00
**2**	0.29	0.14	0.36
**3**	0.26	0.13	0.22
**4**	0.108	0.14	0.12
**5**	0.10	0.09	0.12
**6**	0.07	0.08	0.14
**7**	0.07	0.04	0.13
**8**	0.07	0.01	0.13
**9**	0.08	0.01	0.12
**10**	0.08	0.02	0.09

Data was collected about how many hours of video gaming the children engaged in per week, which kinds of videogames these were, and what controllers were used. Kendall's tau was used to determine correlation between number of hours played and the output of the k-medoids clustering analysis.

There was no significant correlation between number of hours played and the clusters (r_τ = -0.1807, z=-1.4495, p=0.1472). This was not multiversed.

The phi coefficient, implemented using the [sjstats] R package (Lüdecke, 2020), was used to determine the association between clusters and binary responses on questions about game-type and controller-type. Fisher's Exact Test was used to calculate confidence values of coefficients. Significance levels are applied after Bonferroni correction. The results are presented in Table 9.

Table 7 - Phi coefficients for each of the questions regarding gameplay habits in participants.

**Type of Videogame**	**φ**
First-person/Third-person perspective games (e.g. Minecraft, Fortnite)	0.0073
Strategy/Puzzle games (e.g. Chess, Civilization)	0.2183
Sports-based games (e.g. Fifa, Wii Sports)	0.0986
Simulator games (e.g. Flight simulator, NASCAR)	0.1128
Mobile games (e.g. Angry Birds, Candy Crush Saga, Doodle Jump)	0.1338
Other	0.1264
None (child does not play videogames)	0.2160
**Type of Controller**	**φ**
Keyboard	0.0468
Joystick	0.5261**
Games console controller (e.g., Xbox, Playstation, Wii)	0.2594
Handheld non-mobile non-tablet device (e.g. Nintendo, Gameboy)	0.0242
Tablet/smartphone with touchscreen	0.0423
Other	0
None (child does not play videogames)	0.1512

***p<.005, **p<.001, ***p<.0001**

2.5 Dimensionality Reduction

The k-medoids analysis was supplemented with a dimensionality reduction technique called Uniform Manifold Approximation and Projection (UMAP; McInnes et al. 2020). This technique provides a means by which to visualise high-dimensional data on two dimensions whilst preserving more of the global structure than such dimensionality reduction techniques as PCA or t-SNE (Sánchez-Rico & Alvardo, 2020; although see Kobak & Linderman, 2019, for caveats). This allowed us to check the robustness of clustering results and to visualise how the AIs and children compared across all 40 tasks.

Table 8 – Average Silhouette Width for PAM on UMAP output for combined dataset.

Cluster	Average silhouette width post-UMAP
**1**	0.00
**2**	0.66
**3**	0.62
**4**	0.54
**5**	0.46
**6**	0.47
**7**	0.42
**8**	0.37
**9**	0.37
**10**	0.37

2.6 MANOVAs with 'ironbar' and 'Trrrrr'

Both 'ironbar' and 'Trrrrr' were individually compared to children first in terms of the percentiles they were in with respect to the children's performances (see main text). Then they were compared using one-sample Hotelling's T^2 test across all 40 tasks, using the χ^2-distribution and the F-distribution ([desctools] R package, Signorelli et al., 2020). A non-parametric equivalent, Hallin and Paindaveine's (2002a, b) Multivariate Signed-Rank Test (with Tyler Angles) was run, using various settings for the pseudo mahalanobis distance and the p-value computation method. Specifically, the settings for pseudo mahalanobis distance were 'rank', 'sign', or 'normal'. The p-value computation techniques were approximation and bootstrapping (with 1000 permutations) ([ICSNP] R package, Nordhausan, Sirkia, Oja, and Tyler, 2018). Using the F-distribution for the Hotelling's test enabled the computation of Bonferroni and simultaneous confidence intervals adjusted for family-wise error rate, for post hoc comparisons on a task-by-task basis. Simultaneous confidence intervals were too conservative, resulting in impossible values being included in the 95% confidence interval, so Bonferroni confidence intervals were used. The assumption of Normality was violated in most of these cases by the child sample, but there is evidence to suggest that there is some robustness to this when using this kind of analysis (Finch, 2005). Correlation coefficients by level and by task were also generated for 'Trrrrr' and 'Ironbar' individually. All analyses were conducted in R. All plots were generated using [ggplot2] R package (Wickham, 2016) unless otherwise stated. Clustering was performed using the [cluster] (Maechler et al. 2019) and [factoextra] R packages (Kassambara and Mundt, 2020). UMAP was performed with the [umap] R package (Konopka, 2020), and the ShinyApp created using [shiny] and [shinydash] packages (Chang, Cheng, et al. 2020; Chang, Borges Ribeiro, et al., 2020).

3 References

Chang, W., B. Borges Ribeiro, RStudio, Almasaeed Studio, and Adobe Systems Incorporated. 2020. "ShinyDashboard: Create Dashboards with 'Shiny'." *CRAN.*

Chang, W., J. Allaire, J. J. Cheng, Y. Xie, J. McPherson, ..., and R Core Team. 2020. "Shiny: Web Application Framework for R." *CRAN.*

Croux, C., and C. Dehon. 2010. "Influence predictions of the Spearman and Kendall correlation measures." *Statistical Methods and Applications, 19* 497-515.

D., Lakens. 2013. "Calculating and reporting effect sizes to facilitate cumulative science: a practical primer for t-tests and ANOVAs." *Frontiers in Psychology: Cognition.*

Finch, H. 2005. "Comparison of the Performance of Nonparametric and Parametric MANOVA Test Statistics when Assumptions Are Violated." *Methodology, 1 (1)* 27-38. doi:DOI 10.1027/1614-1881.1.1.27.

Hallin, M., and D. Paindaveine. 2002b. "Optimal procedures based on interdirections and pseudo-Mahalanobis ranks for testing multivariate elliptic white noise against ARMA dependence." *Bernoulli 8* 787-815.

Hallin, M., and D. Paindaveine. 2002a. "Optimal tests for multivariate location based on interdirections and pseudo-Mahalanobis ranks." *Ann. Statist. 30* 1103-1133.

Kassambara, A., and F. Mundt. 2020. "factoextra: Extra and Visualize the Results of Multivariate Data Analyses." *R packages, version 1.0.7.*

Kaufman, L., and P. Rousseeuw. 1990. *Finding Groups in Data: An Introduction to Cluster Analysis.* New York: John Wiley & Sons.

Kay, M., and J. Wobbrock. n.d. "ARTool: Aligned Rank Transform for Nonparametric Factorial ANOVAs." *R Package version 0.10.7.* doi:10.5281/zenodo.594511.

Kobak, D., and G. C. Linderman. 2019 [preprint]. "UMAP does not preserve global structure any better than t-SNE when using the same initialisation." *bioRxiv.* doi:https://doi.org/10.1101/2019.12.19.877522.

Konopka, T. 2020. "UMAP: Uniform Manifold Approximation and Projection." *CRAN.*

Lenth, R., H. Singmann, J. Love, P. Buerkner, and M. Herve. 2020. "emmeans: Estimated Marginal Means, a.k.a Least-Squares Means." *CRAN R-Project.*

Lüdecke, D. 2020. "sjstats: Statistical Functions for Regression Models." doi:10.5281/zenodo.1284472.

Maechler, M., P. Rousseeuw, A. Struyf, M. Hubert, and K. Hornik. 2019. "cluster: Cluster Analysis Basics and Extensions." *R package version 2.1.0.*

McInnes, L., J. Healy, and J. Melville. 2020 [preprint]. "UMAP: Uniform Manifold Approximation and Projection for Dimension Reduction." *arXiv.*

Nordhausen, K., S. Sirkia, H. Oja, and D. E. Tyler. 2018. "ICSNP: Tools for Multivariate Nonparametrics." *CRAN.*

Olejnik, S., and J. Algina. 2003. "Generalised Eta and Omega Squared Statistics: Measures of Effect SIze for Some Common Research Designs." *Psychological Methods, 8 (4)* 434-447.

Rousseeuw, P. J. 1987. "Silhouettes: a graphical aid to the interpretation and validation of cluster analysis." *Journal of Computational and Applied Mathematics 20* 53-67.

Sánchez-Rico, M., and J. M. Alvardo. 2020. "Dimensionality reduction techniques as a preliminary step to cluster analysis: A comparison between PCA, t-SNE and UMAP." *9th European Congress of Methodology.*

Signorelli, A., K. Aho, A. Alfons, N. Anderegg, T. Aragon, C. Arachchige, A. Arppe, et al. 2020. "DescTools: Tools for Descriptive Statistics." *CRAN.*

Tabachnick, B. G., and L. S. Fidell. 2001. *Using Multivariate Statistics.* 4th. Needham Heights, MA: Allyn and Bacon.

Wickham, H. 2016. *ggplot2: Elegant Graphics for Data Analysis.* New York: Springer-Verlag.

Wobbrock, J. O., L. Findlater, D. Gergle, and J. J. Higgins. 2011. "The Aligned Rank Transform for Nonparametric Factorial Analyses Using Only ANOVA Procedures." *Proceedings of the ACM Conference on Human Factors in Computing Systems (CHI 2011).* Vancouver, BC, Canada. 143-146. doi:10.1145/1978942.1978963.

4 Outline of 'Get the Fruit!' game

All images of the Animal-AI Environment and Testbed below are licensed under Apache License, Version 2.0 (http://www.apache.org/licenses/LICENSE-2.0)

Dashboard information and classes of objects in game:

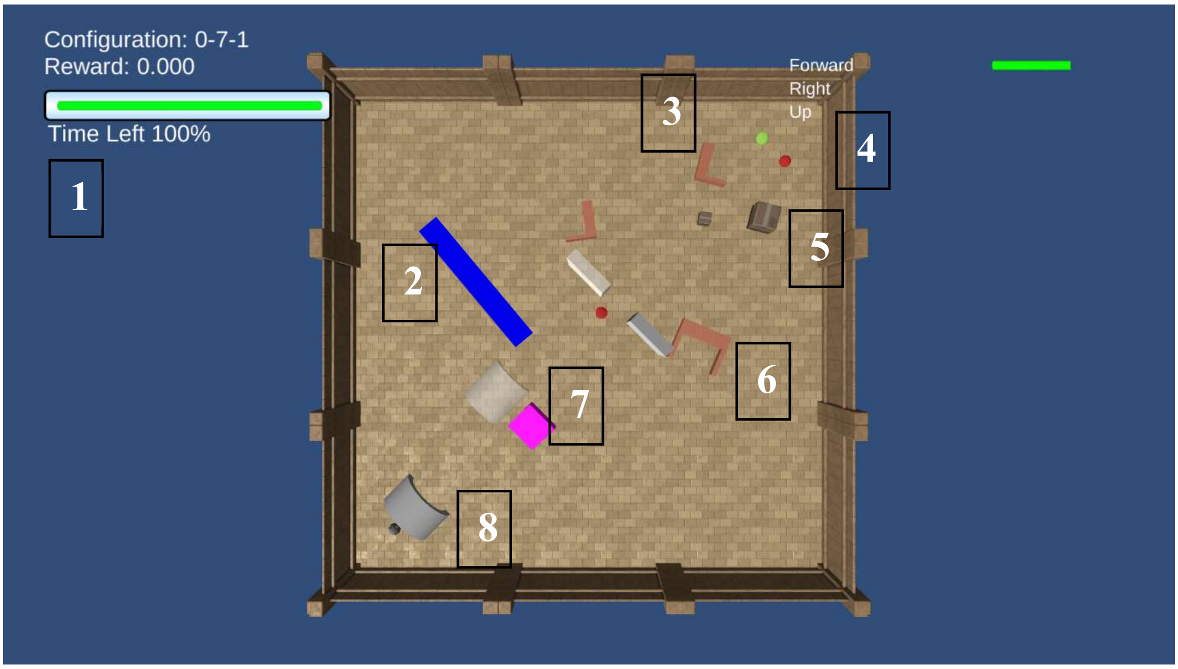

1. Configuration = task code. Reward = number of 'points' accrued. Starts at 0 and goes down with time, shown by green time bar and percentage. This turns red when 20% of the time or less is left, acting as an exogenous cue. Points go up by 1 when a yellow 'fruit' (not shown, see below) is retrieved.
2. Blue platforms. There is no way to ascend on to these, unless a ramp is available (7). In some tasks, the participant begins on a blue platform; once they move of it, they cannot go back, enabling forced choice tasks to be generated.
3. Green 'fruit' is the target of the level. It needs to be retrieved within the time limit and after all yellow 'fruit' have been retrieved in order to pass. All fruit can be stationary OR moving, at varying directions, speeds, and accelerations.
4. Red 'fruit' should be avoided. It is 'poisonous'. Touching it causes immediate failure of the level.
5. These are 'cardboard boxes', they are pushable and move around easily.
6. These are pushable blocks. They take more effort to push.
7. Ramps allow movement in the third dimension. They are always pink. They cannot be moved
8. Other obstacles are opaque or transparent, and can be of varying sizes and shapes, including tunnels.

Follow this link for the tutorial video presented to the children:
https://www.youtube.com/watch?v=oA9WHMPAONM

You may also play the full game presented to the children by contacting the corresponding author.

Levels and pass requirements:

There are two stages to the game: Tutorial levels (involving 15 tasks – humans only) and Test levels (involving 40 tasks for both humans and AIs). Tutorial tasks were created to introduce the rules and parameters of the game. Test tasks were randomly selected from the 900 available in the Animal-AI Olympics test battery. 4 tasks for each level were randomly selected, and then for the human participants, one of the three variants was selected. The numbering system below refers to the configuration. For example, '2-29-1' refers to the second level, the 29th task, and its 1st variant in the AAI test battery.

Tutorial 0-1-1

This is an empty arena so participants can practice the controls (arrow keys/ASWD). All participants automatically pass.

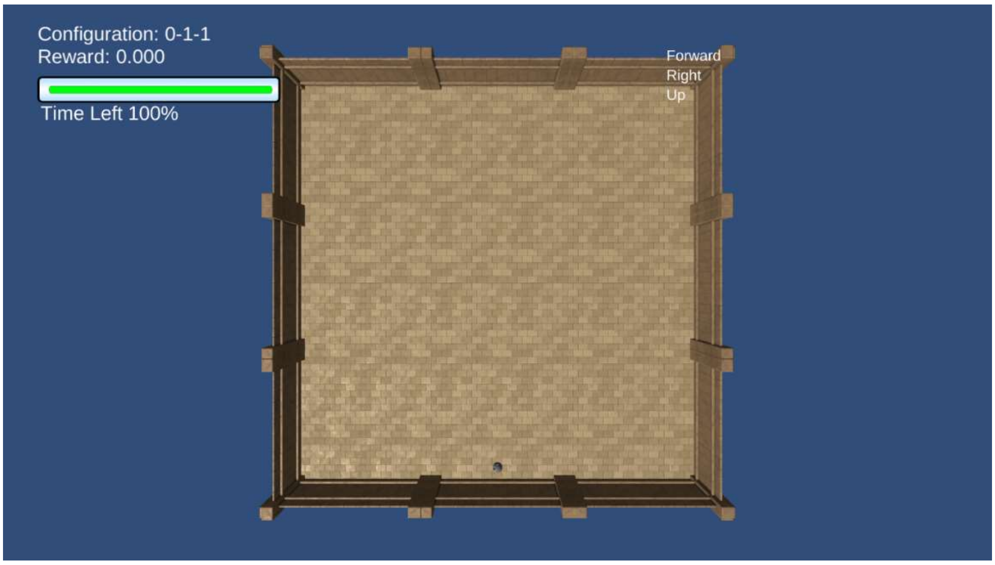

Tutorial 0-2-1

Simple retrieval of green 'fruit' from simple starting position. Participant passes if they successfully get it within the time limit.

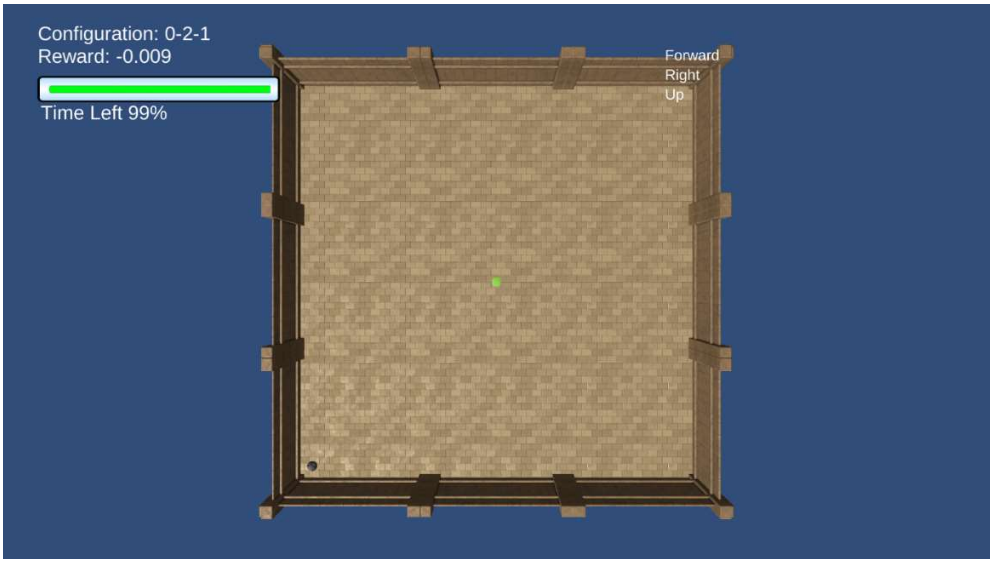

Tutorial 0-2-2

This level introduces yellow ‘fruit’ which give you 1 point each. It also explains that if there is no green ‘fruit’ available, collect as many yellow ‘fruit as you can. Participants pass if they collect both ‘fruit’ within the time limit.

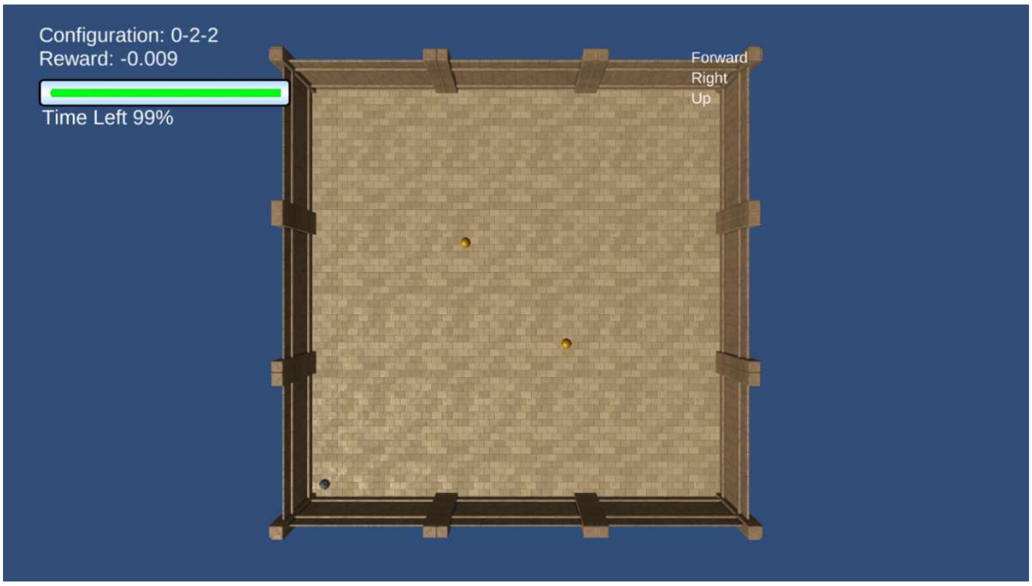

Tutorial 0-2-3

This level introduces ‘fruit’ of different sizes, explaining that larger ‘fruit’ are more preferable. Participants only pass if they retrieve the larger green ‘fruit’.

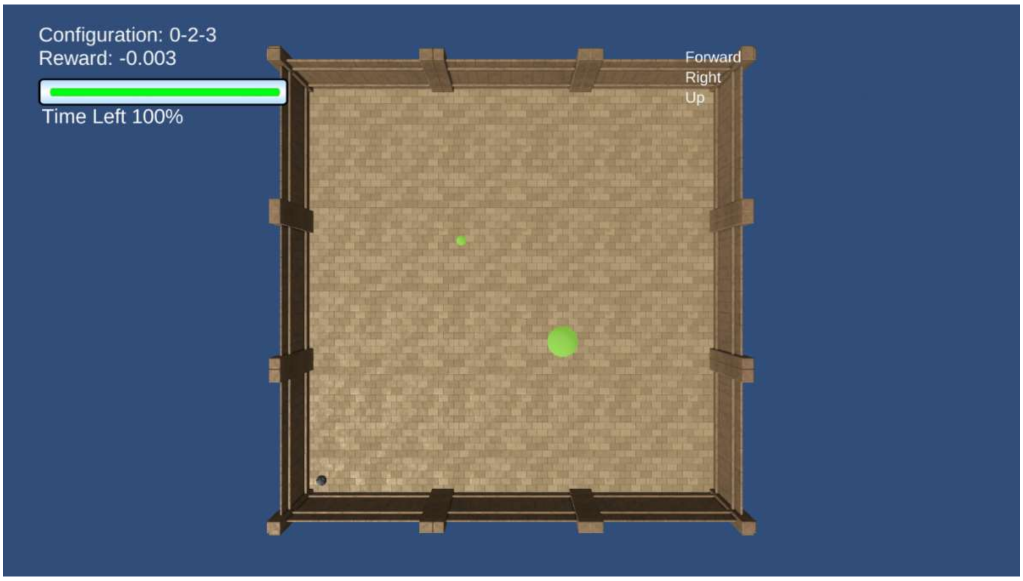

Tutorial 0-2-4

This level synthesises the previous two, explaining that to pass the participant must retrieve the maximum number of points possible, in this case, collecting the two yellow ‘fruit’ **before** the large green one.

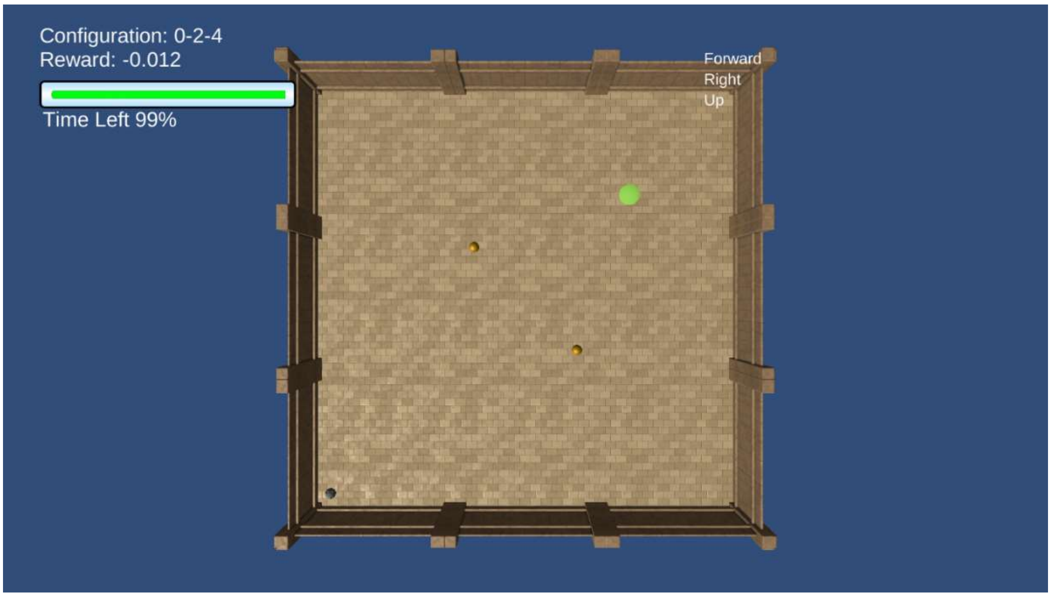

Tutorial 0-3-1

This level describes that the aim is to get the maximum points possible. Here, there are no green ‘fruit’ so success is determined by selecting the side containing 2 yellow ‘fruit’. Also outlines the role of blue platforms. These cannot be re-ascended (unless there is a pink ramp), resulting in forced-choice tasks like this one.

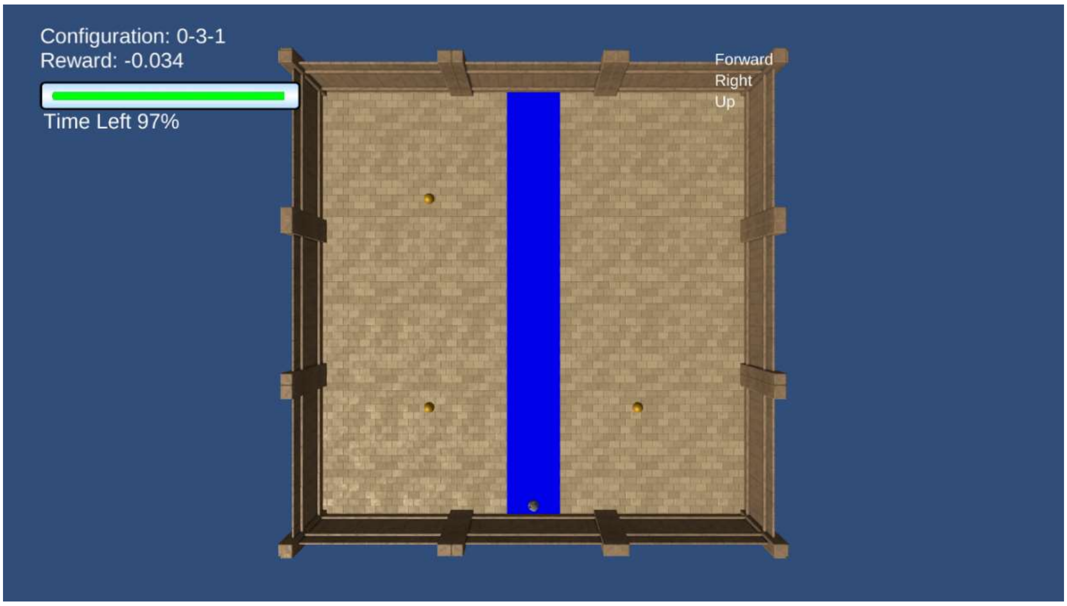

Tutorial 0-4-1

This level allows the participant to explore the different kinds of stationary objects they might encounter, including pink ramps of different heights and opaque vs. transparent obstacles of varying sizes. The ‘lights go out’ periodically, to expose participants to this possibility, in which there is no visual feedback for a few timesteps. All participants automatically pass.

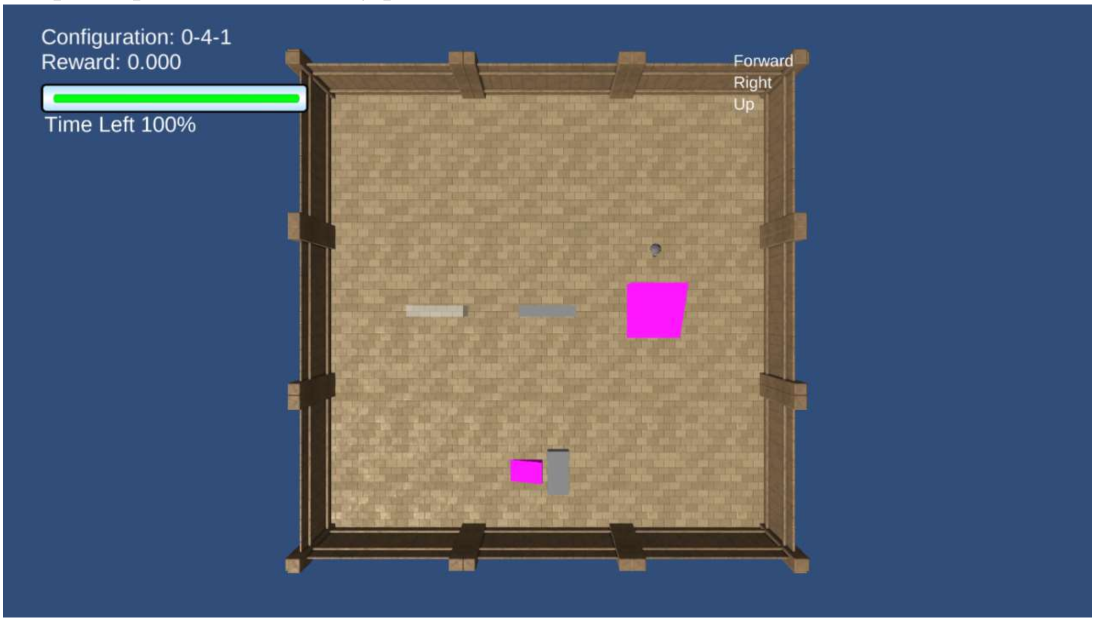

Tutorial 0-5-1

This level allows the participant to explore the ‘dangers’ of the game. Orange ‘hot zones’ accelerate point decrement per time step so shouldn’t be stepped on for very long. Red ‘lava zones’ and ‘poisonous red fruit’ cause immediate failure if they are touched. Participants pass if they do not touch the red objects and don’t spend too long in the orange zones.

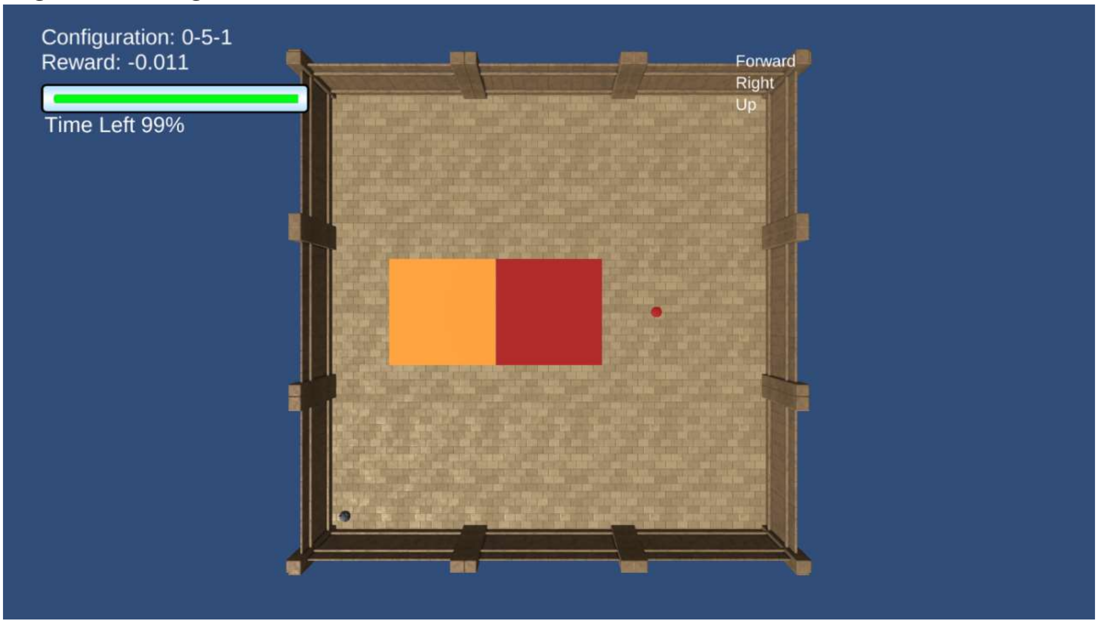

Tutorial 0-6-1

This level allows the participant to explore the different kinds of pushable object, namely the 'boxes' and the pushable blocks, helping them generate an understanding of their affordances. All participants automatically pass.

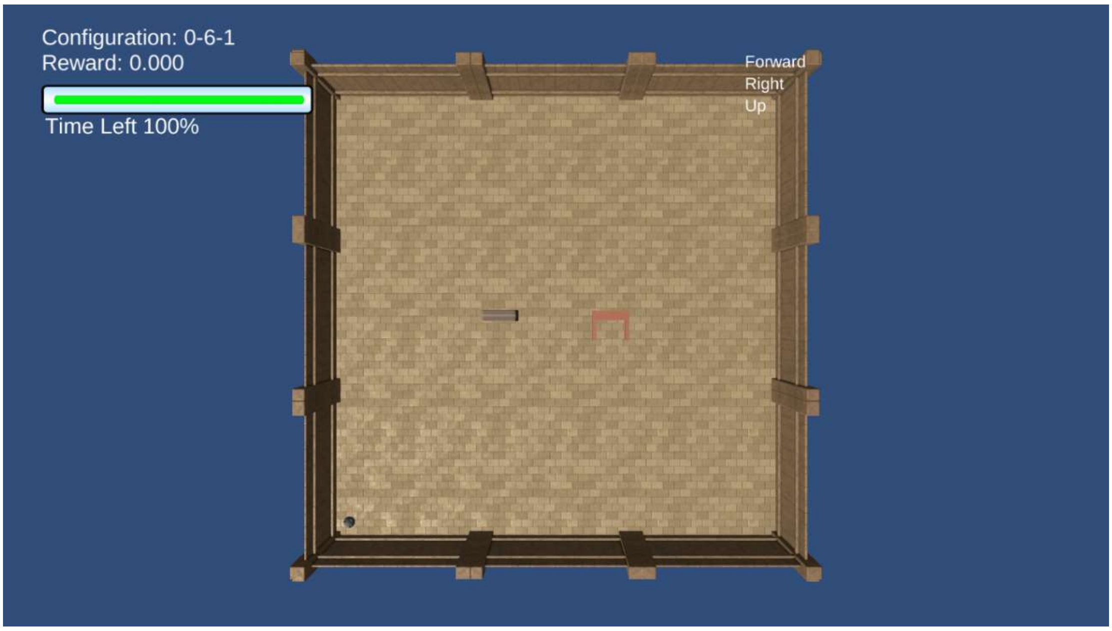

Tutorial 0-7-1

This level includes a random arrangement of the objects previously introduced. The random generator is seeded so every participant views the same random arrangement. The participant passes as long as they do not touch the red 'fruit'.

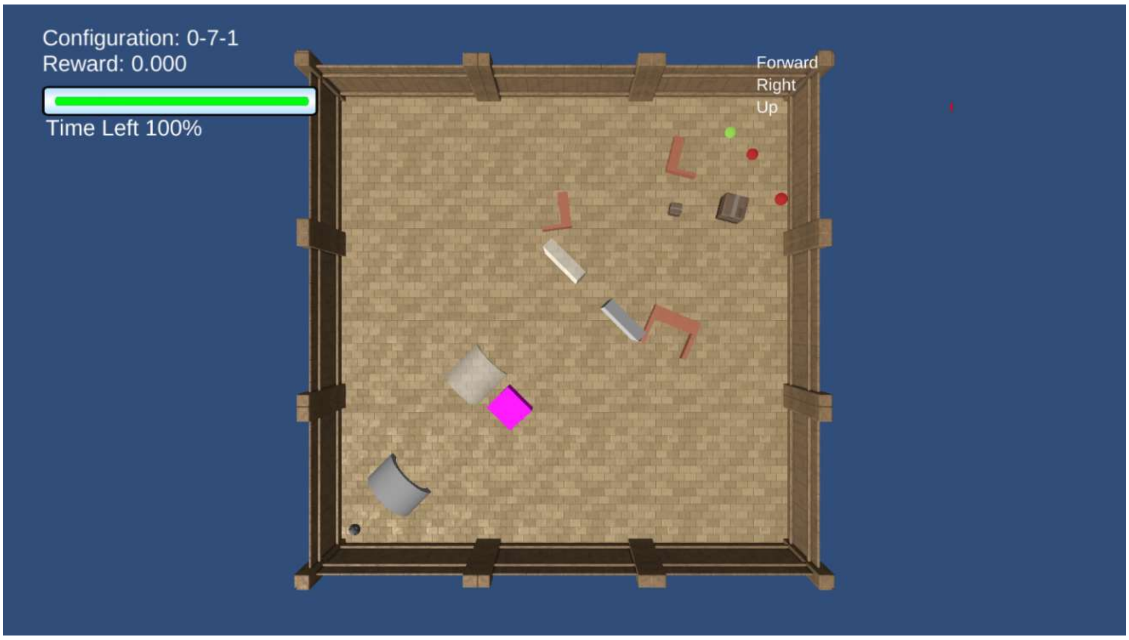

Test 1-6-2

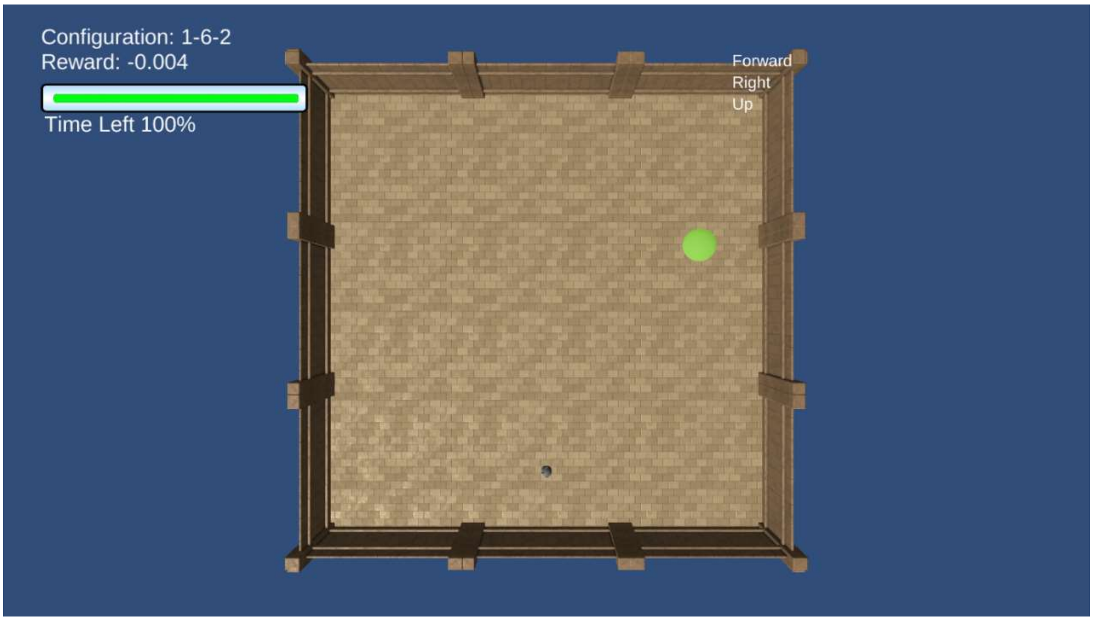

Pass criterion: retrieve green ‘fruit’ within time limit.

Test 1-21-1

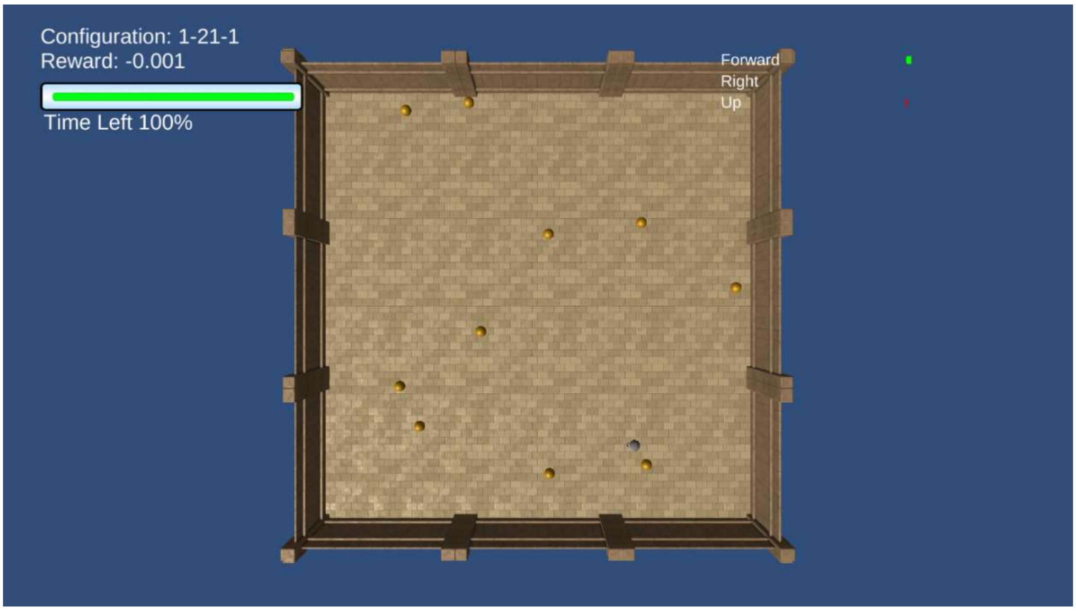

Pass criterion: retrieve all yellow ‘fruit’ within time limit.

Test 1-4-3

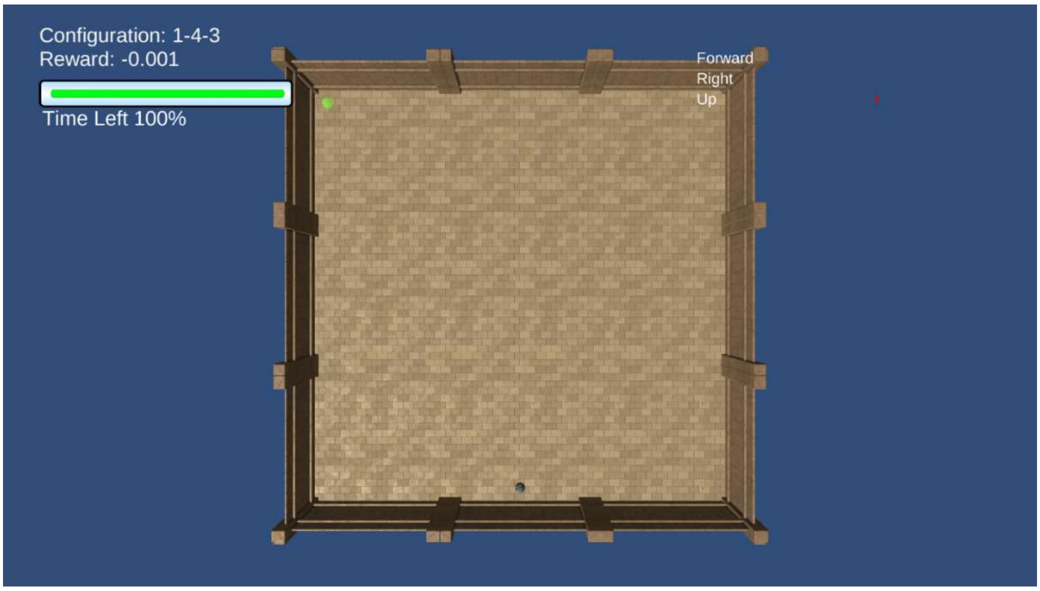

Pass criterion: retrieve green ‘fruit’ within time limit.

Test 1-23-1

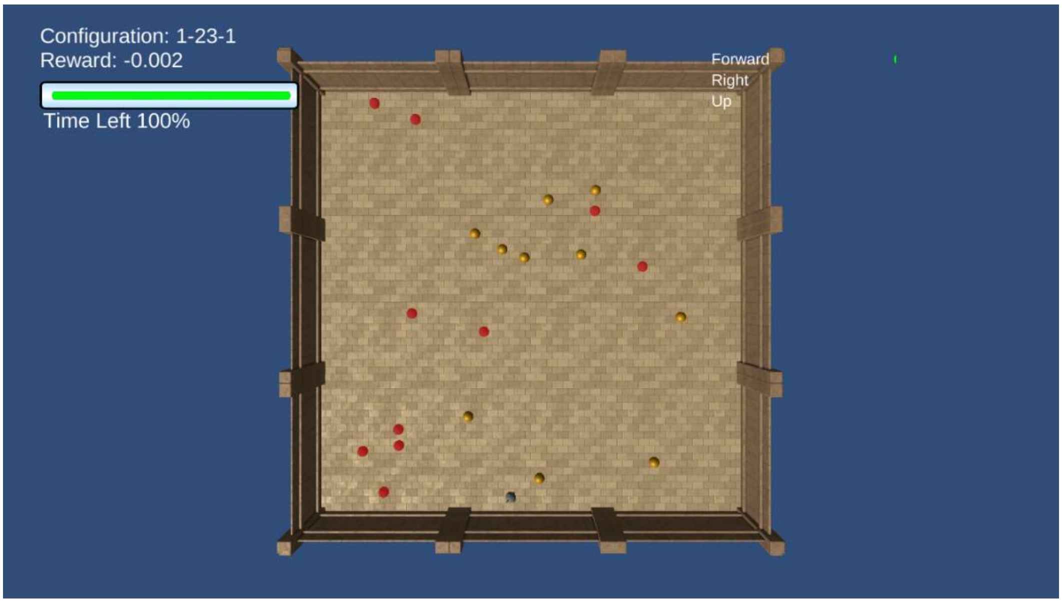

Pass criterion: retrieve all yellow ‘fruit’ without touching the red ‘fruit’ within the time limit.

Test 2-10-1

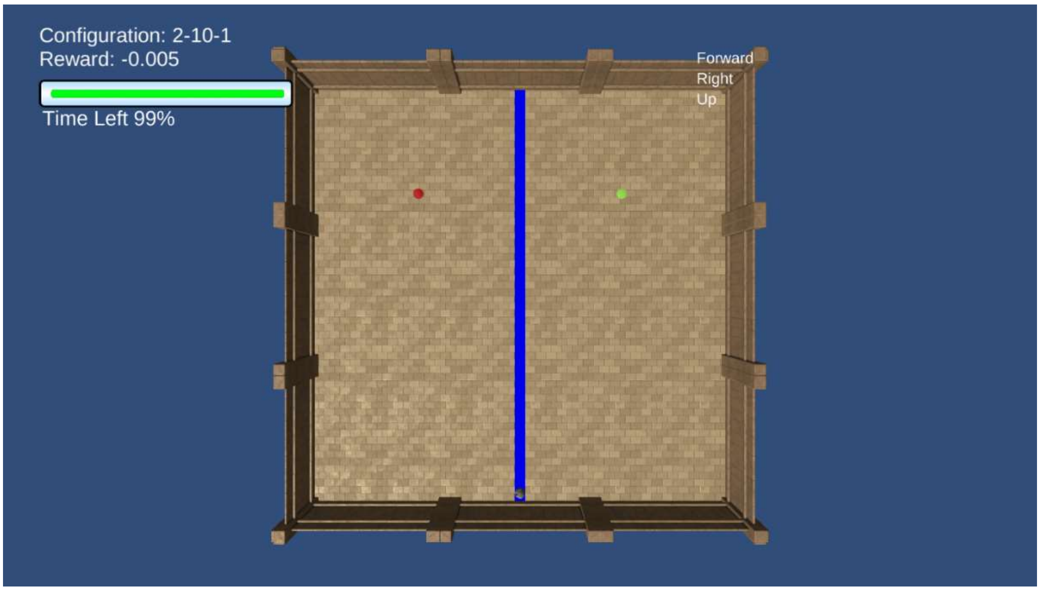

Pass criterion: forced-choice task, must select right side and retrieve green ‘fruit’ in order to pass.

Test 2-17-1

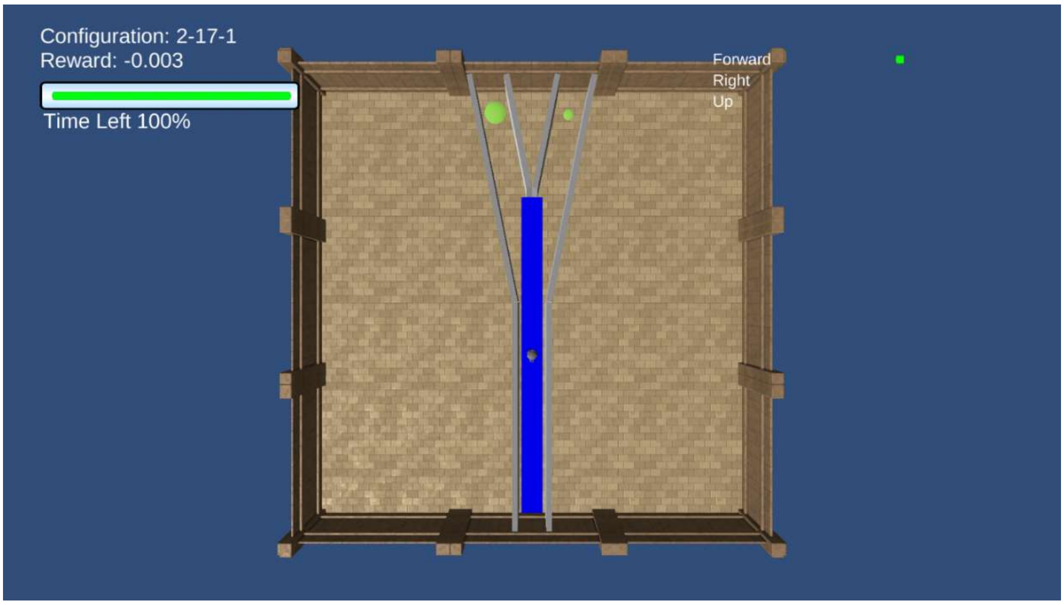

Pass criterion: Forced-choice Y-maze task, requires selection of left side to retrieve larger green ‘fruit’ within the time limit.

Test 2-29-1

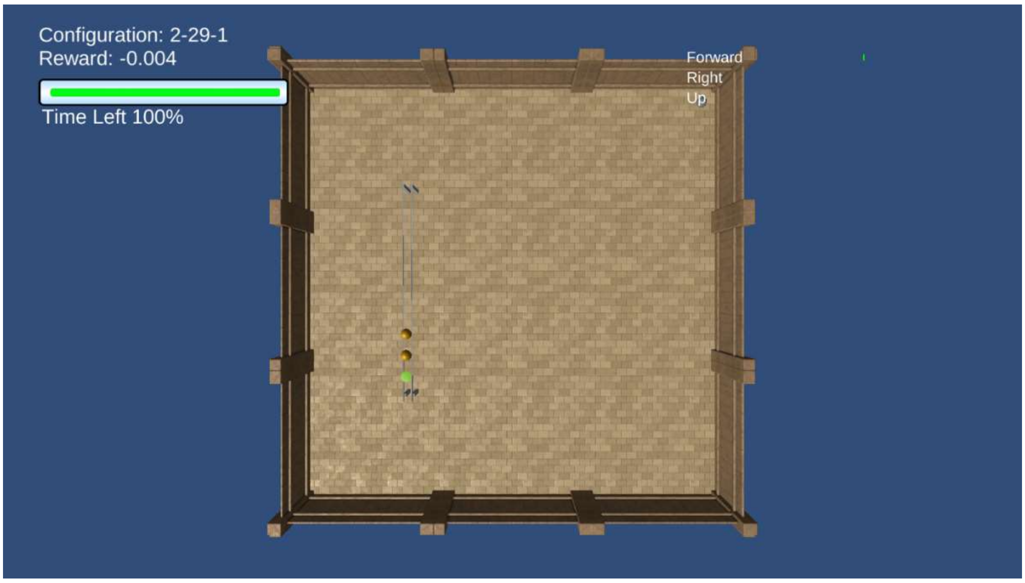

Pass criterion: the 'fruit' roll off a runway, to pass the participant must collect **both** yellow 'fruit' **before** retrieving the green 'fruit', within the time limit.

Test 2-2-1

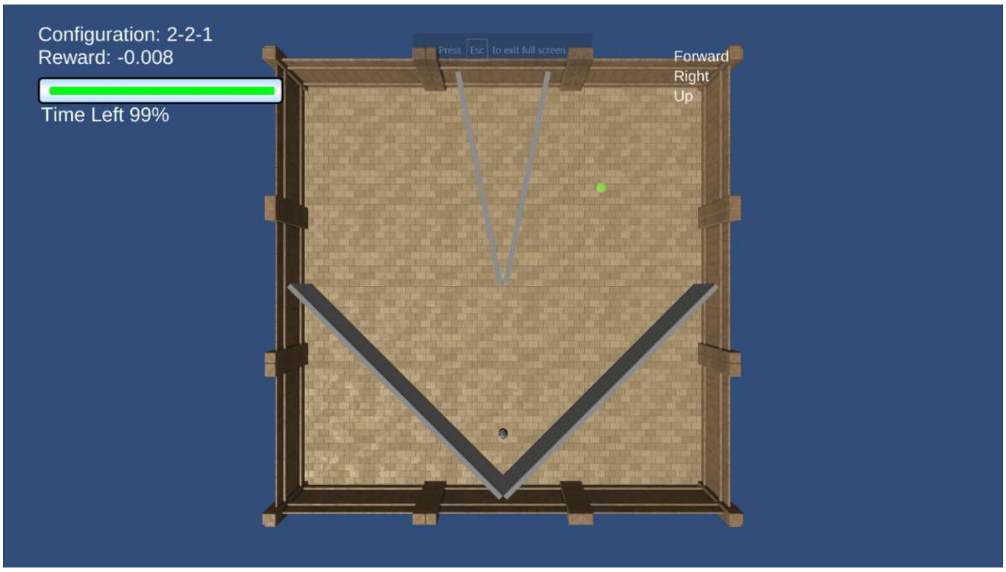

Pass criterion: free-choice Y-maze task, the participant must retrieve the green 'fruit' within the time limit.

Test 3-9-1

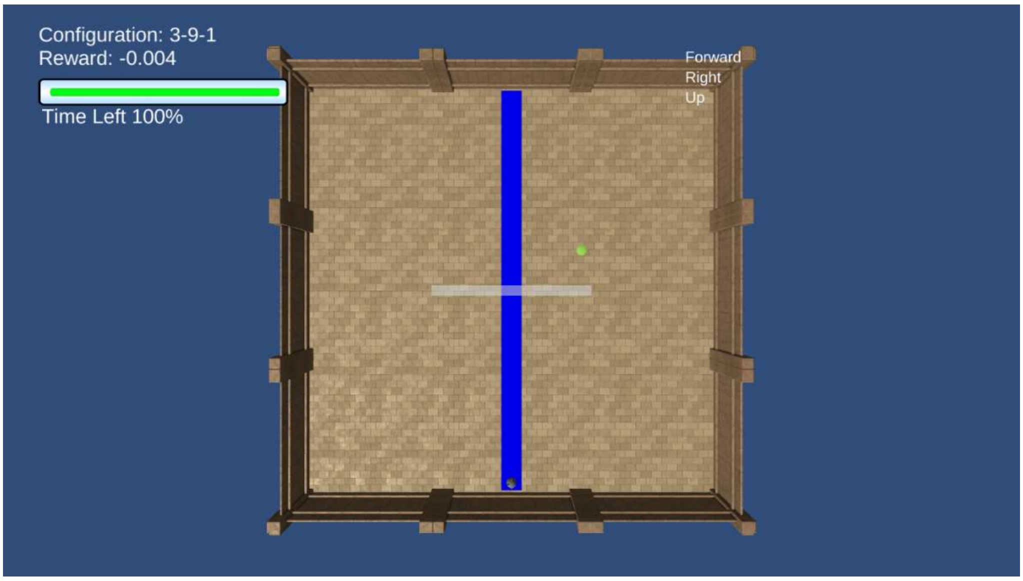

Pass criterion: forced-choice task, the participant must choose the right side and retrieve the green ‘fruit’ partly obscured by the transparent barrier, within the time limit.

Test 3-11-1

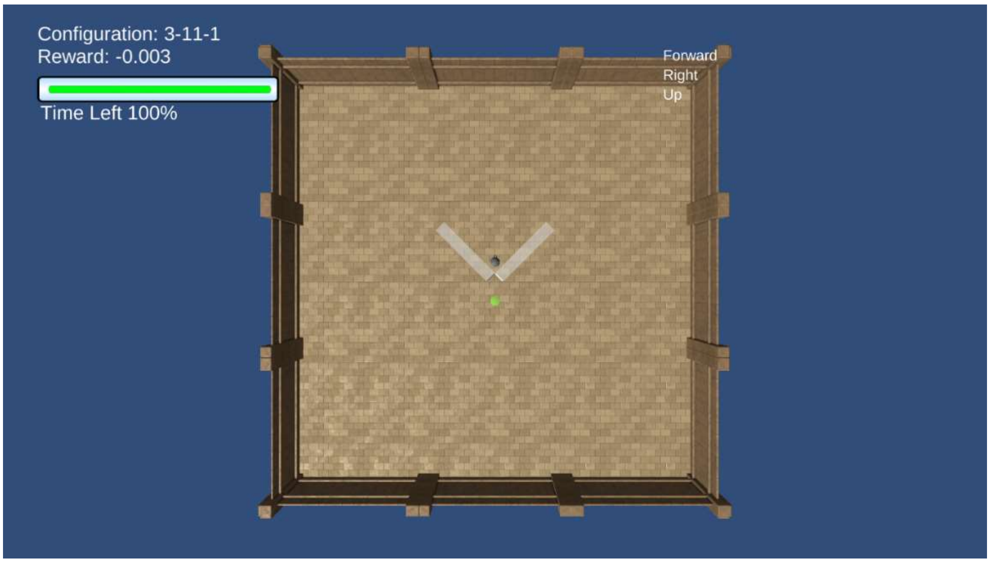

Pass criterion: inverted Y-maze task, to pass the participant must navigate around the Y-maze to obtain the green fruit that is partially obscured by the transparent barrier, within the time limit.

Test 3-21-1

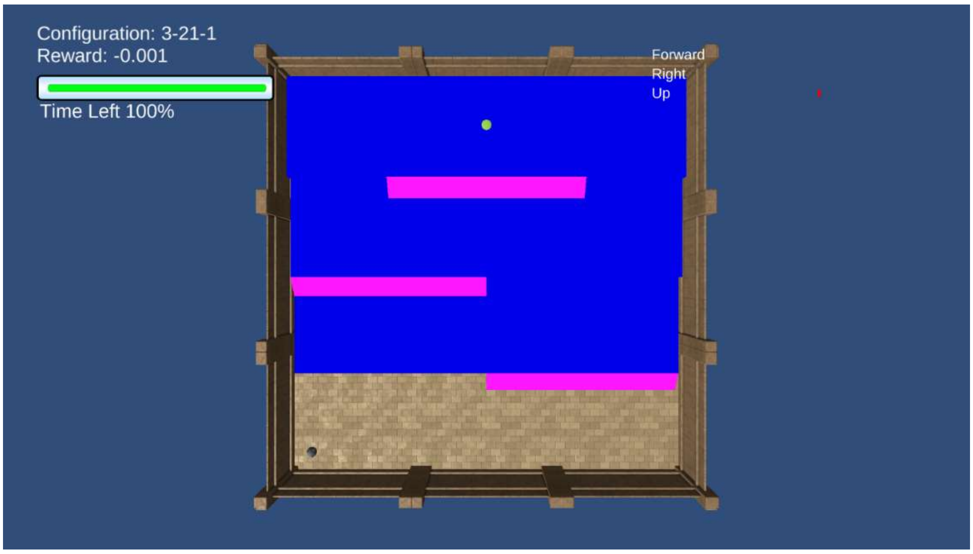

Pass criterion: to pass the participant must ascend the ramps in order to obtain the partly visible green 'fruit' on the third platform, within the time limit.

Test 3-18-1

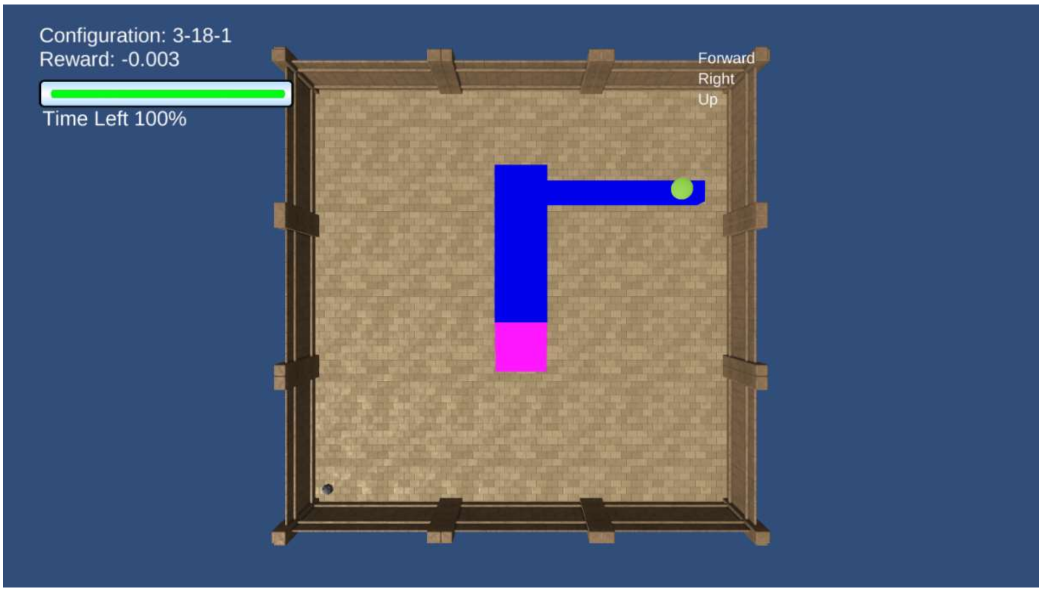

Pass criterion: to pass the participant must ascend the ramp and navigate across the platform in order to obtain the green 'fruit' within the time limit.

Test 4-3-1

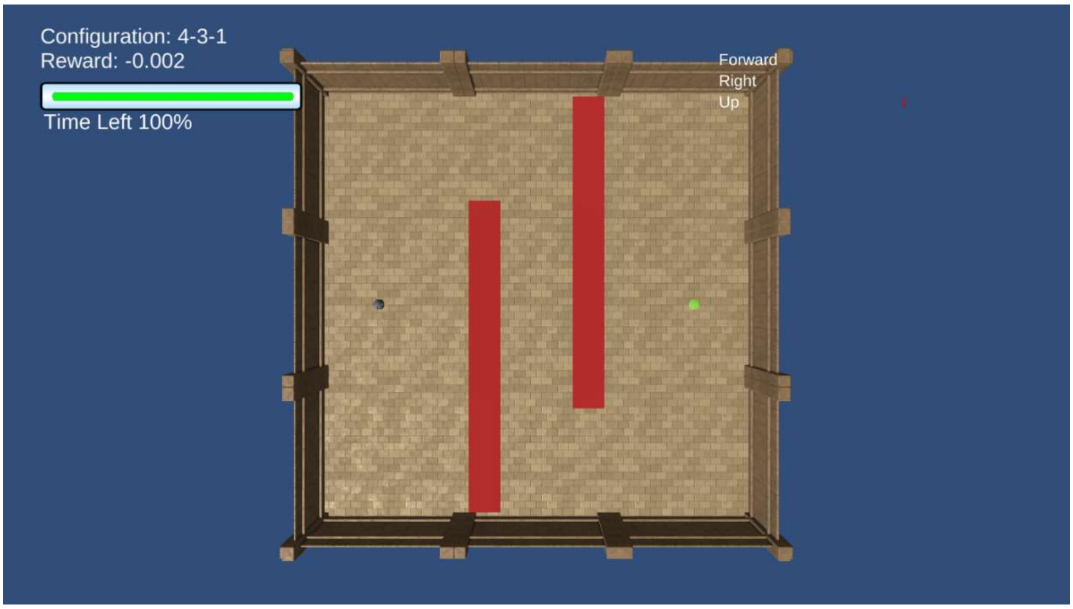

Pass criterion: to pass the participant must navigate around the ‘lava zones’ and obtain the green ‘fruit’ within the time limit.

Test 4-16-1

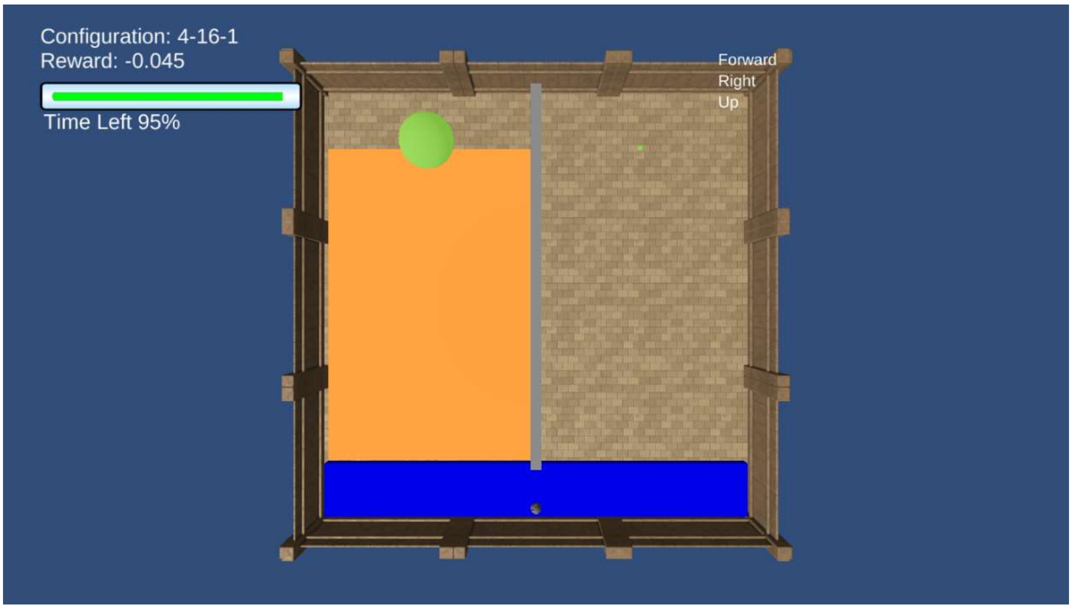

Pass criterion: Forced-choice cost-benefit analysis. To pass the participant must choose the left side and quickly cross the ‘hot zone’ to retrieve the much larger green ‘fruit’, within the time limit.

Test 4-13-1

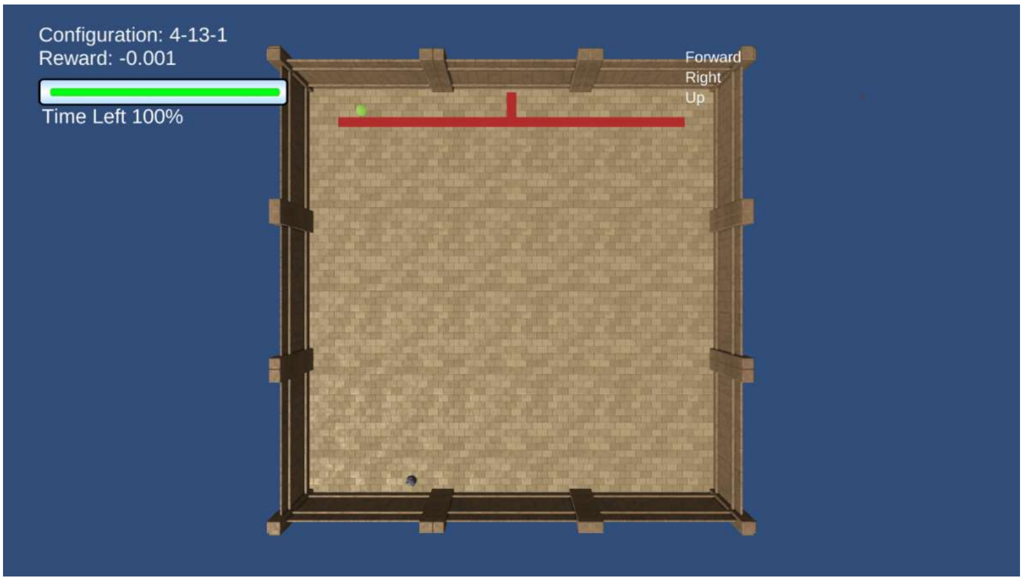

Pass criterion: free-choice T-maze. To pass the participant must navigate around the ‘lava zone’ and retrieve the green ‘fruit’ within the time limit.

Test 4-22-1

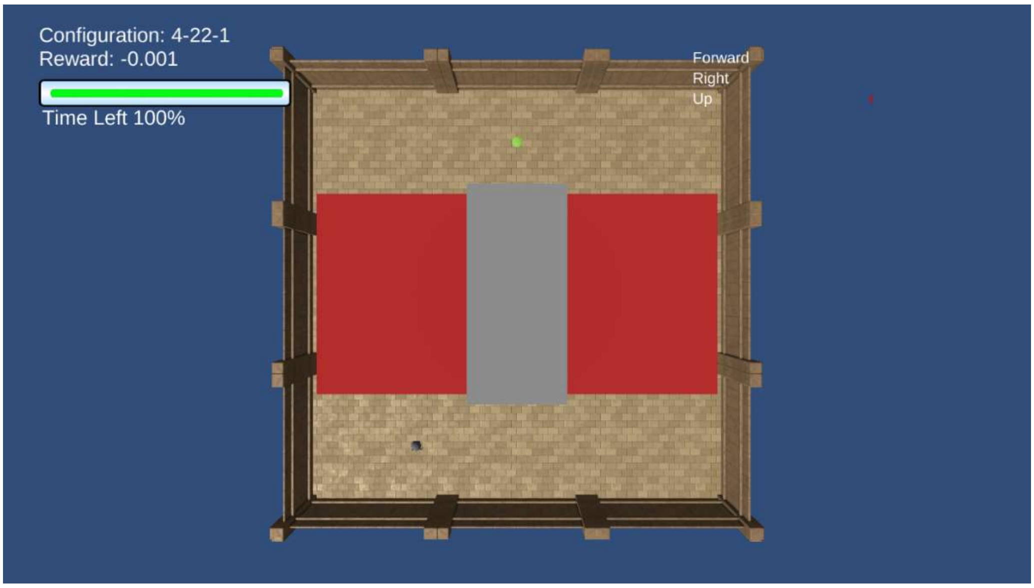

Pass criterion: to pass the participant must navigate across the ‘bridge’ to retrieve the green ‘fruit’ within the time limit.

Test 5-15-1

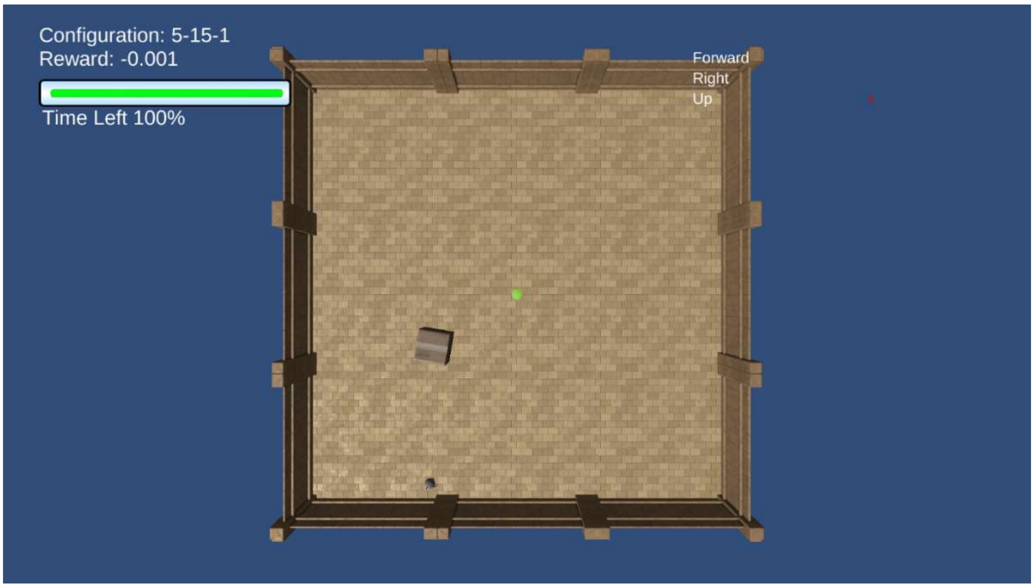

Pass criterion: the green ‘fruit’ is perched on a pole and is unreachable. The participant must push the ‘box’ towards it to knock the ‘fruit’ off so that they can retrieve it, within the time limit.

Test 5-9-1

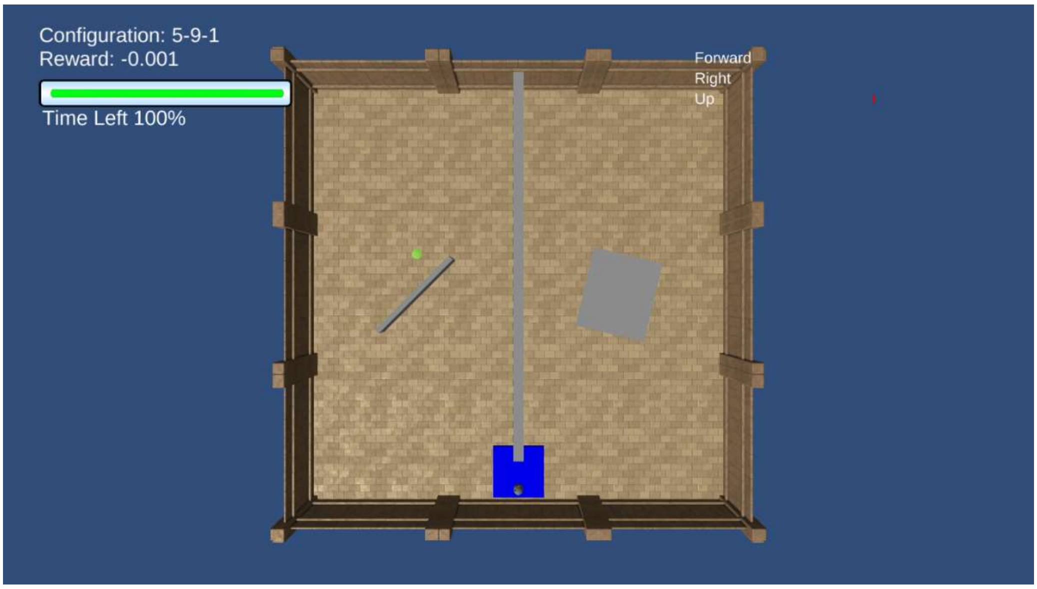

Pass criterion: forced-choice spatial elimination task. The participant must select the right side and navigate behind the barrier to obtain the green ‘fruit’ within the time limit.

Test 5-24-1

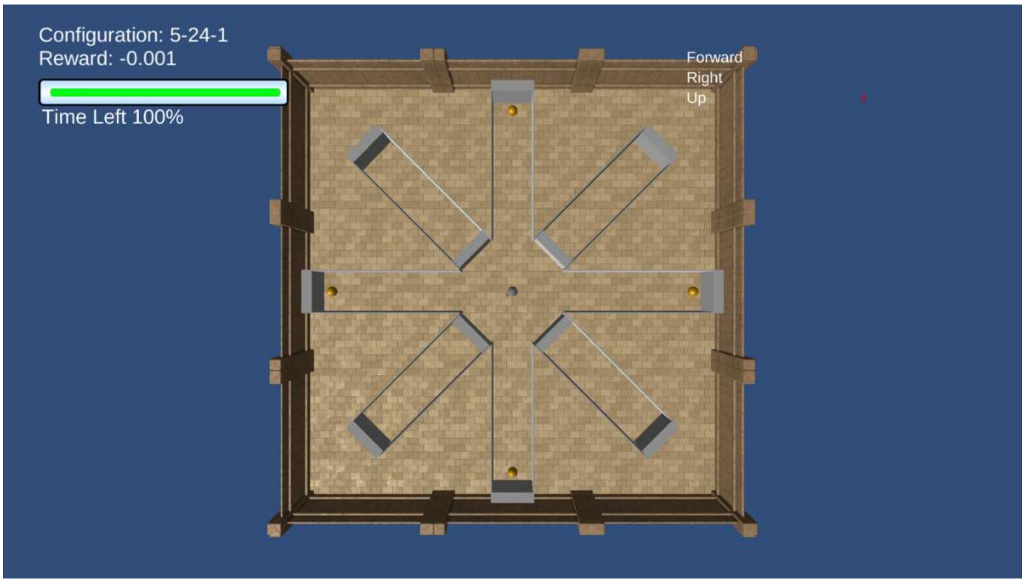

Pass criterion: 4-arm radial arm maze. To pass the participant must retrieve all 4 yellow ‘fruit’ within the time limit.

Test 5-26-1

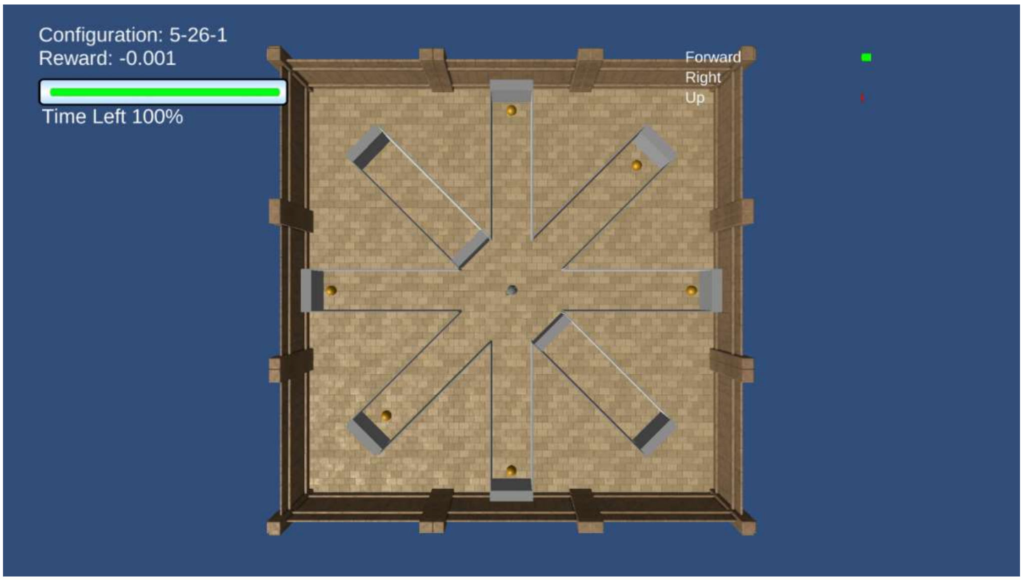

Pass criterion: 6-arm radial arm maze. To pass the participant must retrieve all 6 yellow ‘fruit’ within the time limit.

Test 6-11-1

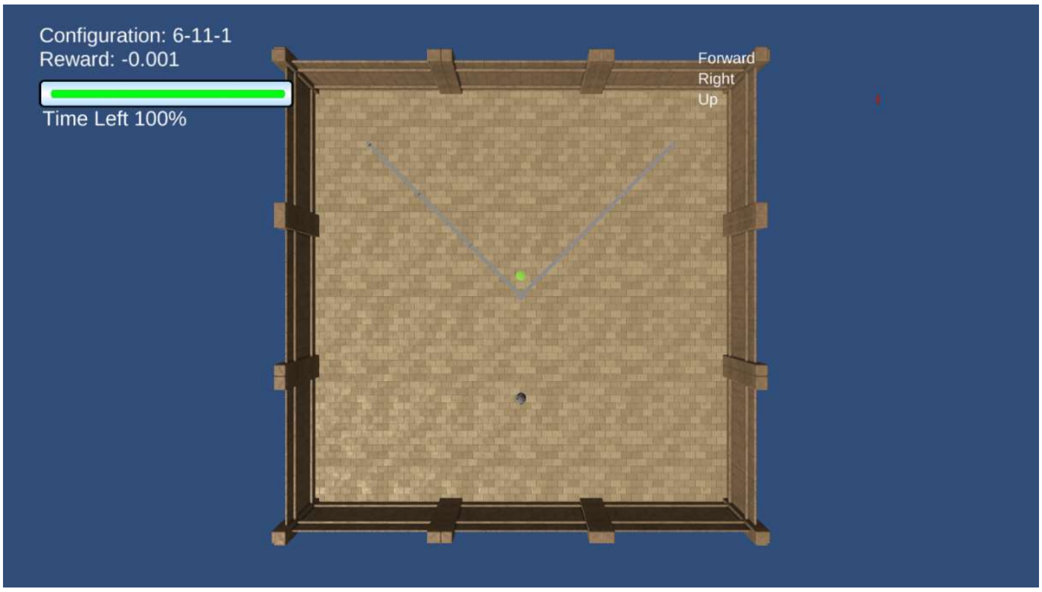

Pass criterion: Inverted Y-maze variant. To pass the participant must navigate around the barrier (shaped like a fence so that the goal is visible through it), to obtain the green 'fruit' within the time limit.

Test 6-29-2

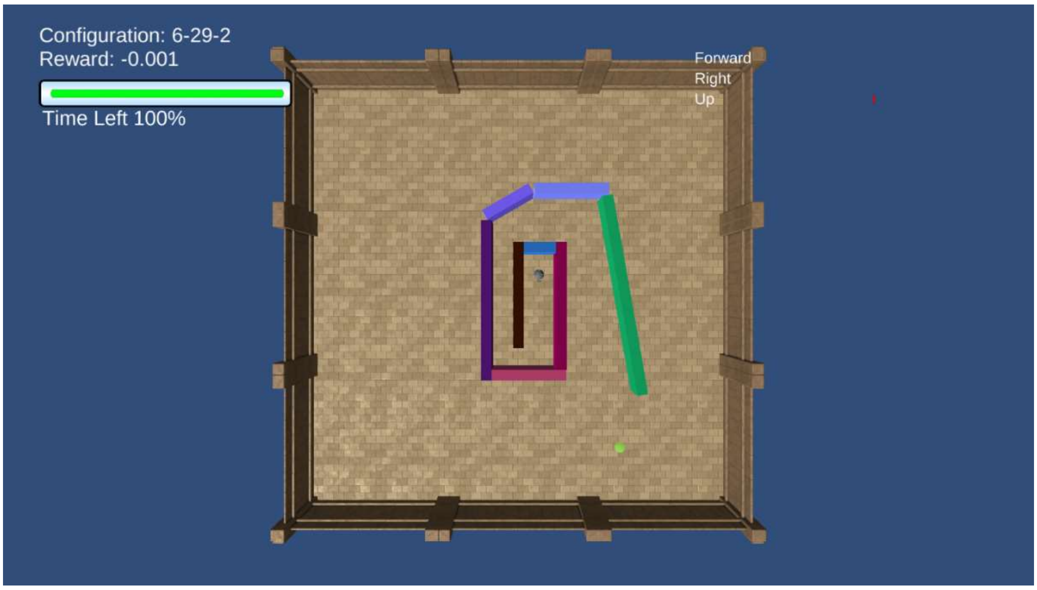

Pass criterion: The participant must navigate out of the simple maze and retrieve the green 'fruit' within the time limit.

Test 6-9-1

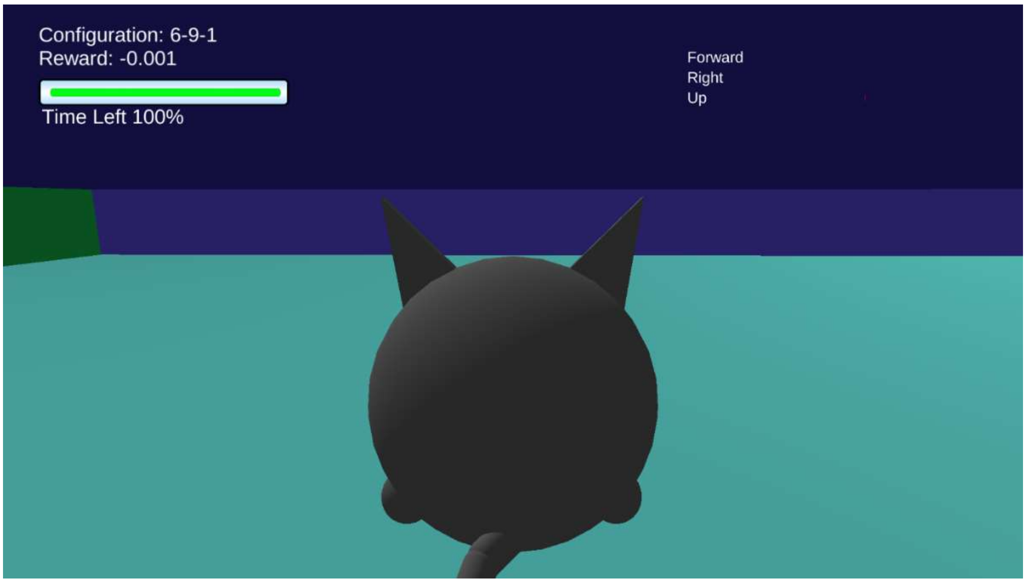

Pass criterion: participant must navigate within the oddly coloured obstacle-less environment to retrieve the green ‘fruit’ (not visible) within the time limit.

Test 6-12-2

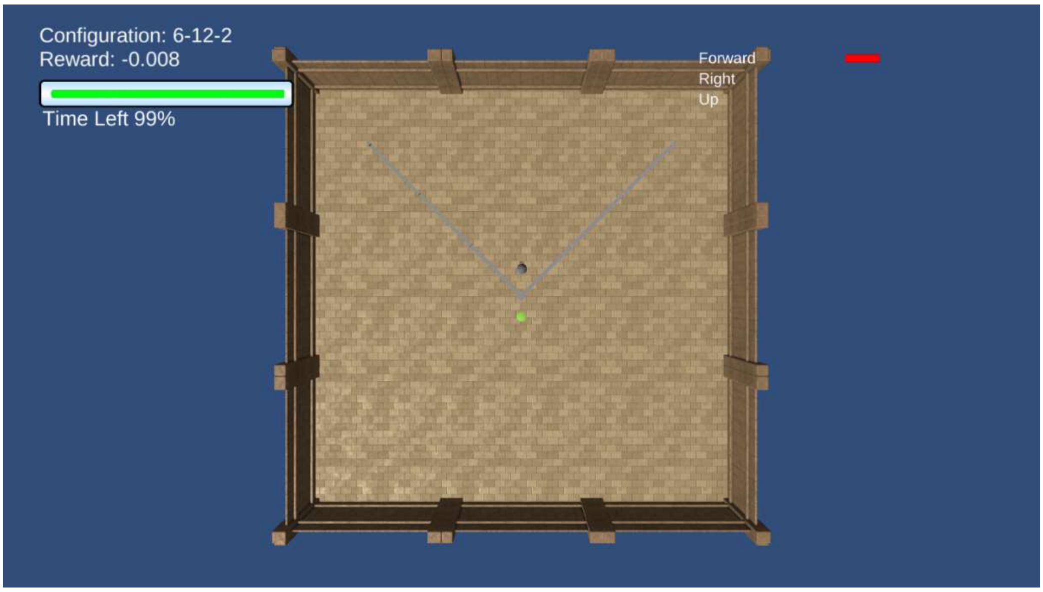

Pass criterion: Inverted Y-maze variant. To pass the participant must navigate around the barrier (shaped like a fence so that the goal is visible through it), to obtain the green ‘fruit’ within the time limit.

Test 7-16-1

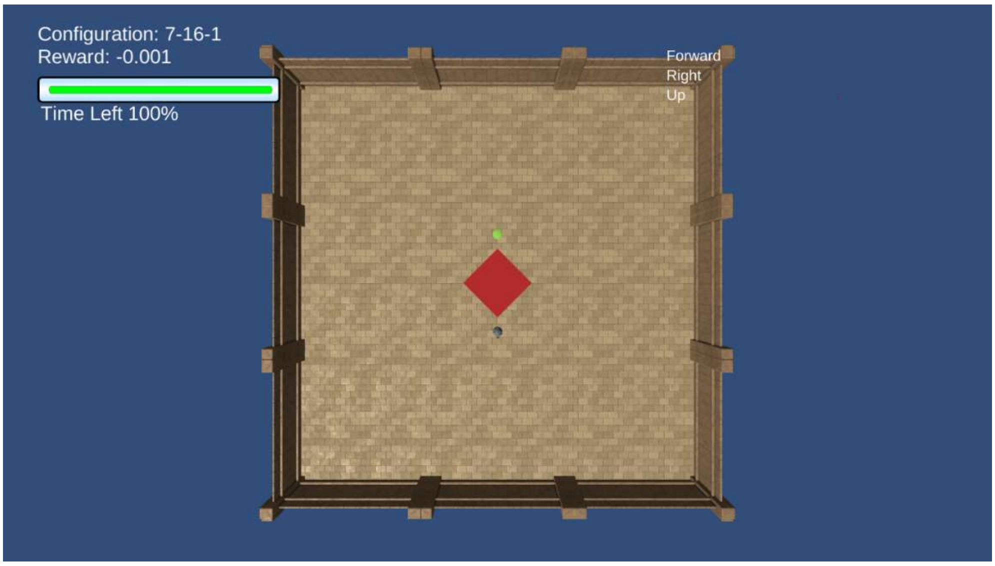

Pass criterion: participant must navigate around the ‘lava zone’ to retrieve the green ‘fruit’ within the time limit. The ‘lights go out’, meaning all visual feedback is withheld, periodically for a few timesteps.

Test 7-17-1

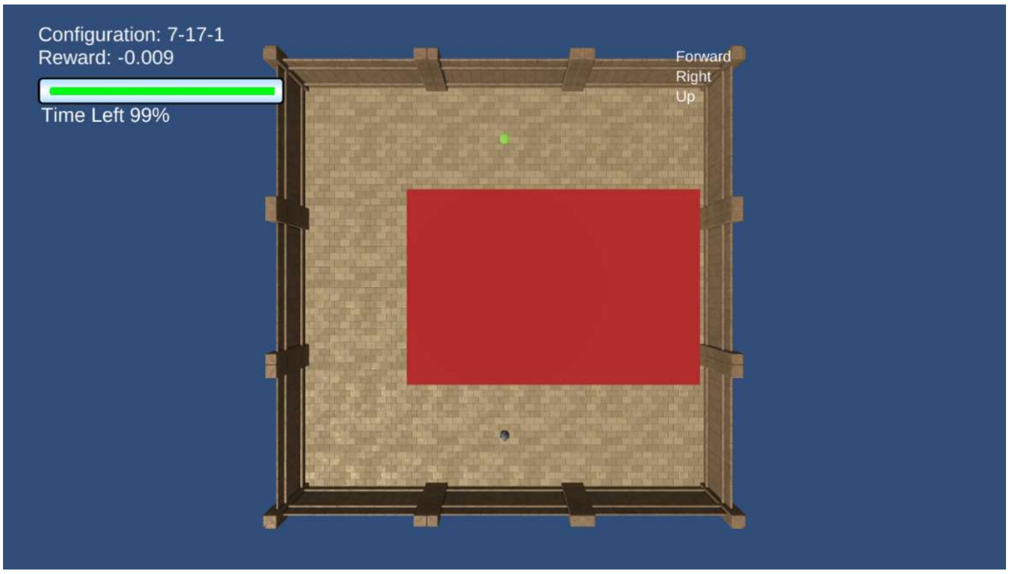

Pass criterion: participant must navigate around the ‘lava zone’ to retrieve the green ‘fruit’ within the time limit. The ‘lights go out’, meaning all visual feedback is withheld, periodically for a few timesteps.

Test 7-22-1

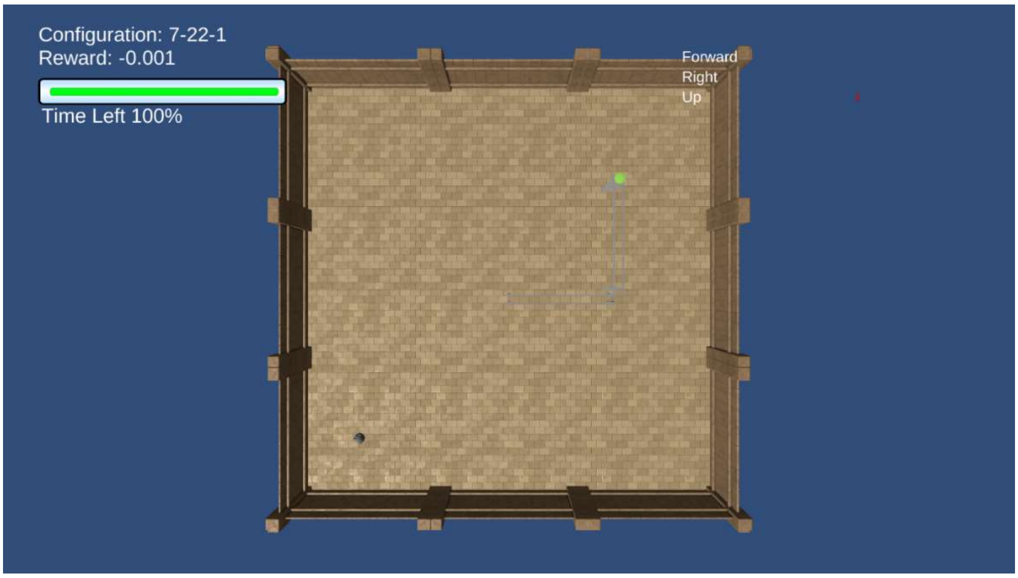

Pass criterion: the participant must obtain the green ‘fruit’ that falls down the runway, which includes a right-angled kink, within the time limit.

Test 7-25-1

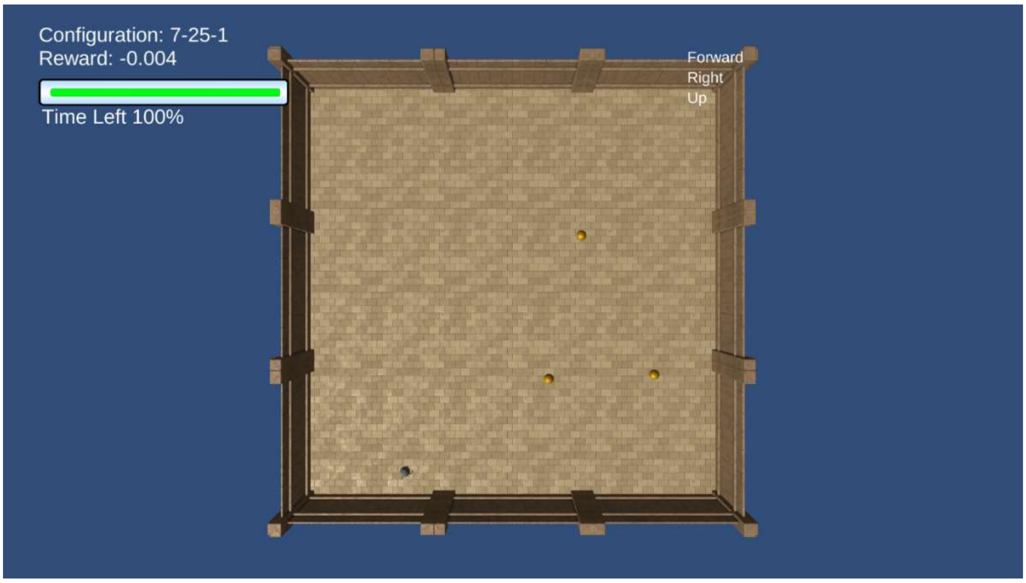

Pass criterion: the participant must obtain all yellow ‘fruit’ within the time limit. The ‘light go out’ periodically and remain off towards the end of the level.

Test 8-3-3

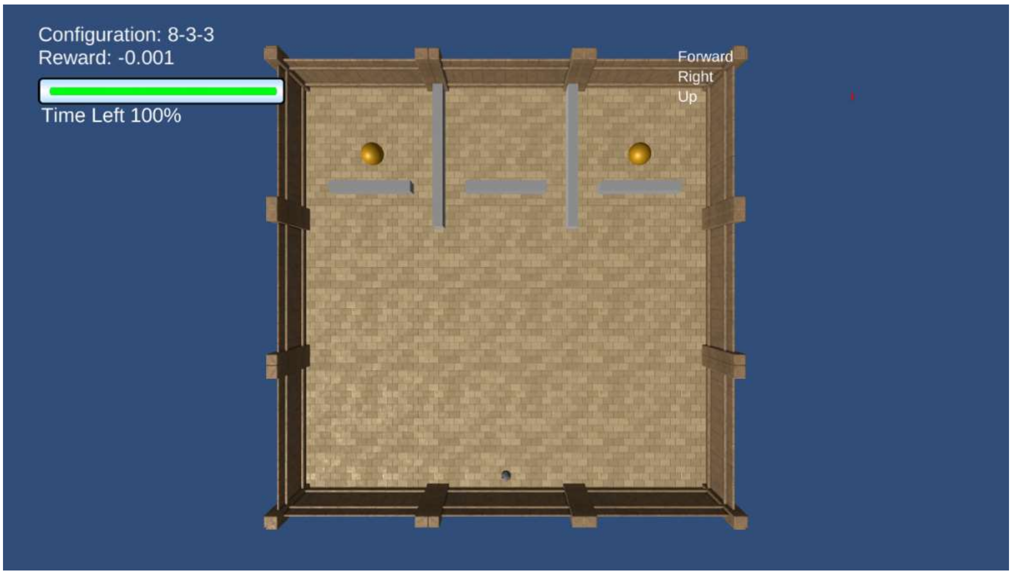

Pass criterion: free-choice object permanence test. To pass the participant must obtain both yellow 'fruit' within the time limit. The yellow 'fruit' are visible initially as they drop behind the barriers from a height.

Test 8-19-1

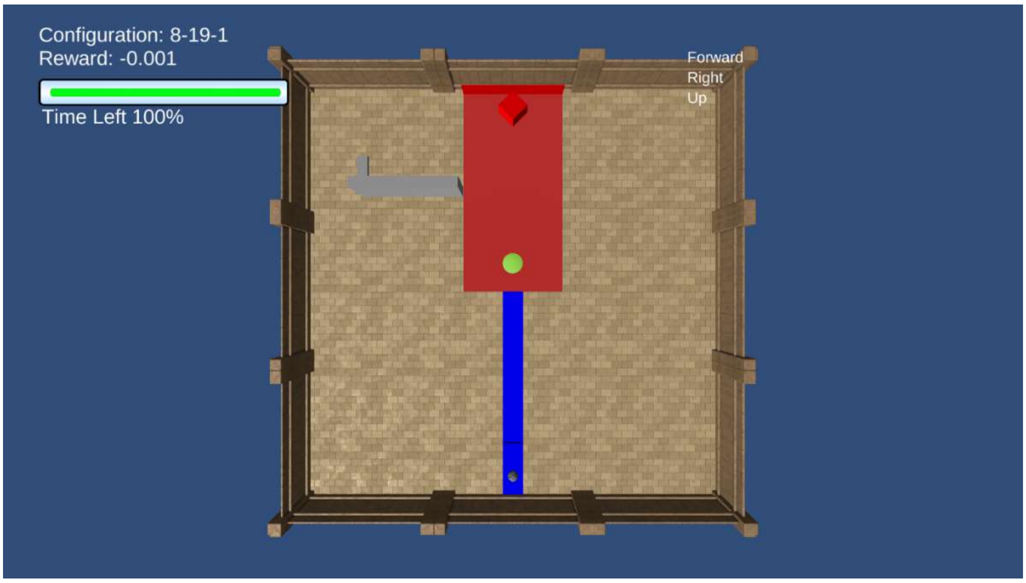

Explanation and pass criterion: forced-choice object permanence/spatial elimination test. The green 'fruit' rolls away in view of the participant. The 'light go out' as it approaches the red block, and come back on with the green 'fruit' not visible. To pass the participant must choose the left side and retrieve the green 'fruit' now behind the opaque barrier, within the time limit.

Test 8-30-1

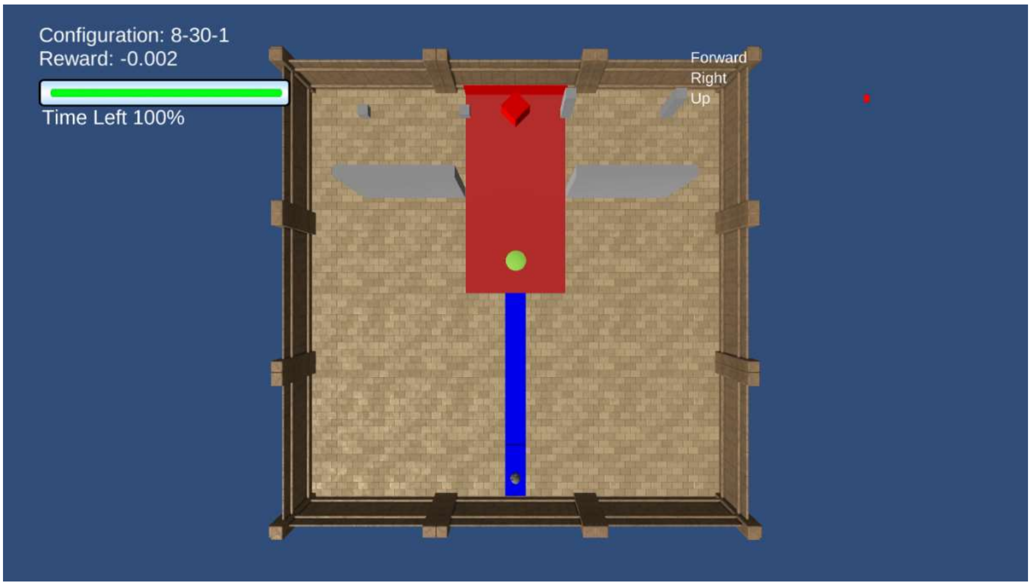

Explanation and pass criterion: similarly to 8-19-1, however, as the 'lights go out', the opaque barriers on both sides start to drop. When they come back on, the green 'fruit' is not visible, the left barrier is resting on the shorter blocks and is visibly not far enough from the arena floor to be hiding a green 'fruit', and the right barrier is resting on the taller blocks and *is* far enough from the arena floor. To pass, the participant must choose the right side and navigate around the barrier to retrieve the green 'fruit', within the time limit.

Test 8-11-1

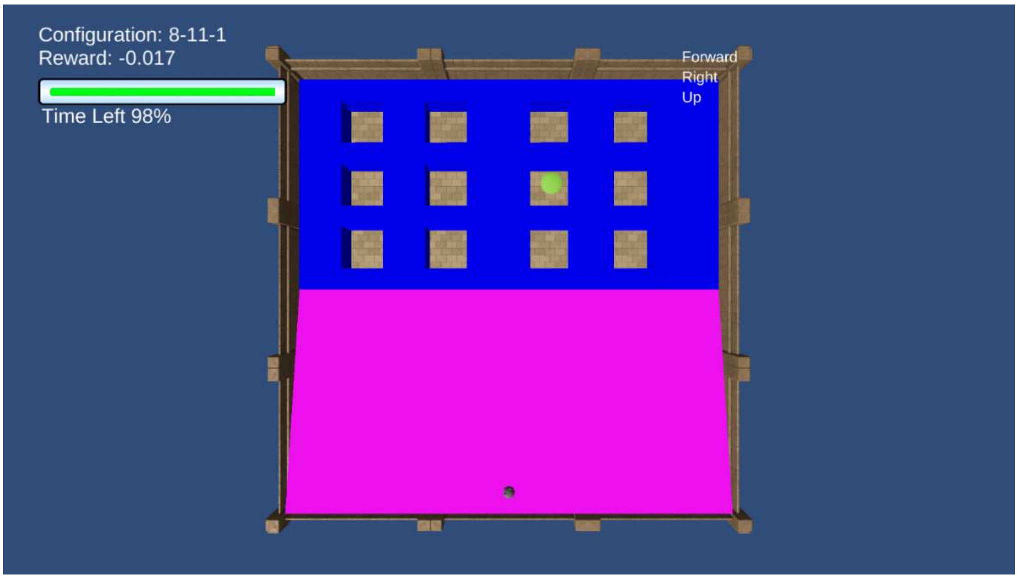

Pass criterion: the green 'fruit' visibly drops into the hole pictured. The participant must navigate to the correct hole and drop down to obtain the green 'fruit' within the time limit.

Test 9-21-1

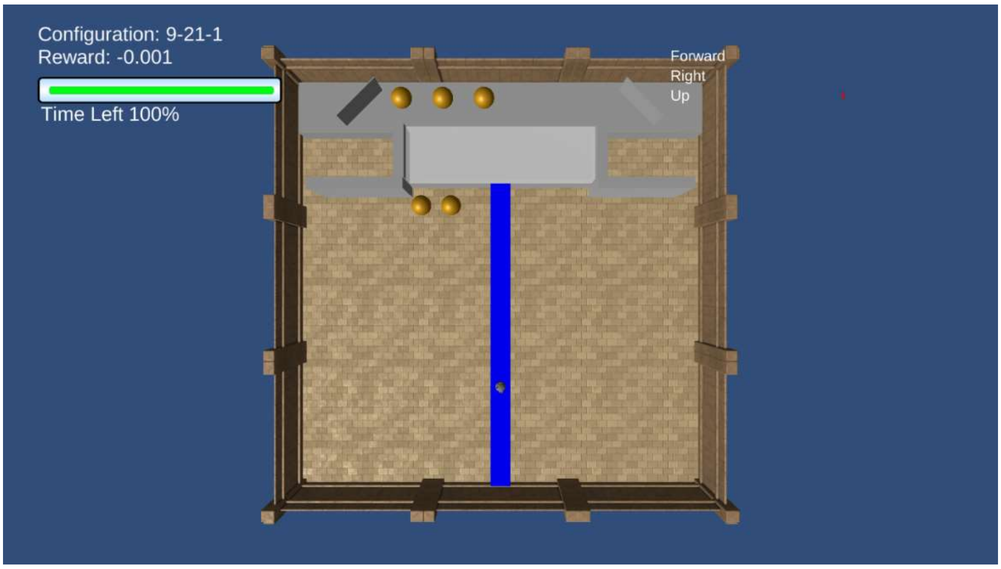

Explanation and pass criterion: forced choice numerosity task. The three yellow ‘fruit’ roll rightwards and drop behind the right barrier. To pass, the participant must choose the right side and navigate behind the barrier to collect the three yellow ‘fruit’, within the time limit.

Test 9-24-1

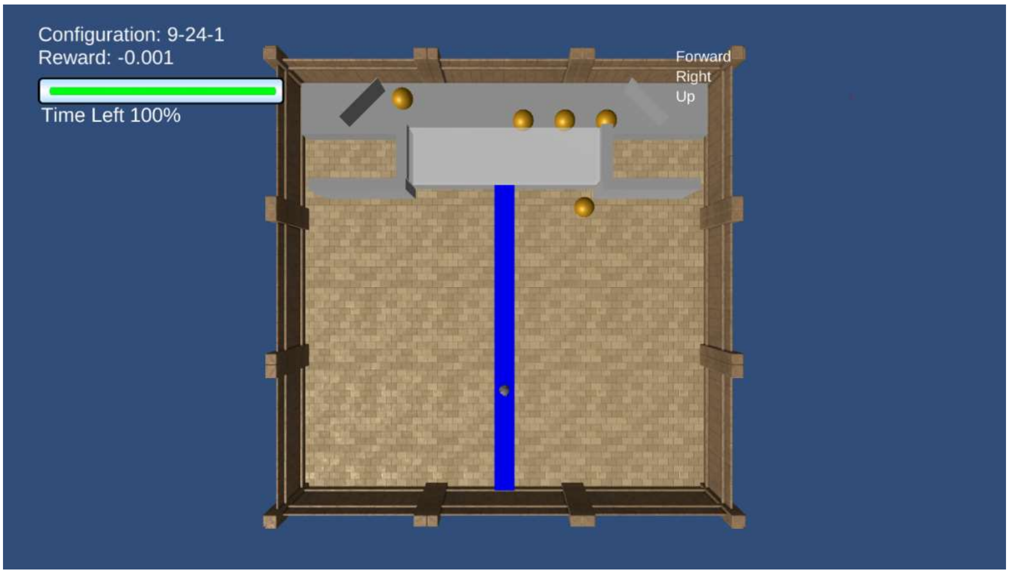

Explanation and pass criterion: forced choice numerosity task. The three yellow ‘fruit’ on the right roll leftwards and drop behind the left barrier. The one yellow ‘fruit’ on the right rolls rightwards and drops behind the right barrier. To pass, the participant must choose the left side and navigate behind the barrier to collect the three yellow ‘fruit’, within the time limit.

Test 9-8-1

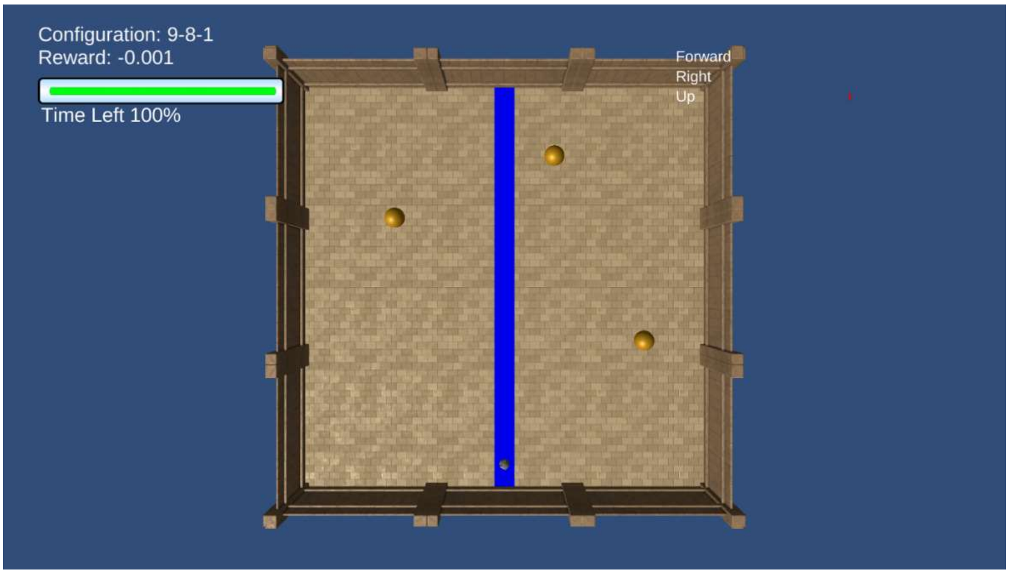

Pass criterion: forced choice numerosity task. To pass, the participant must select the right side and collect both yellow 'fruit', within the time limit.

Test 9-3-1

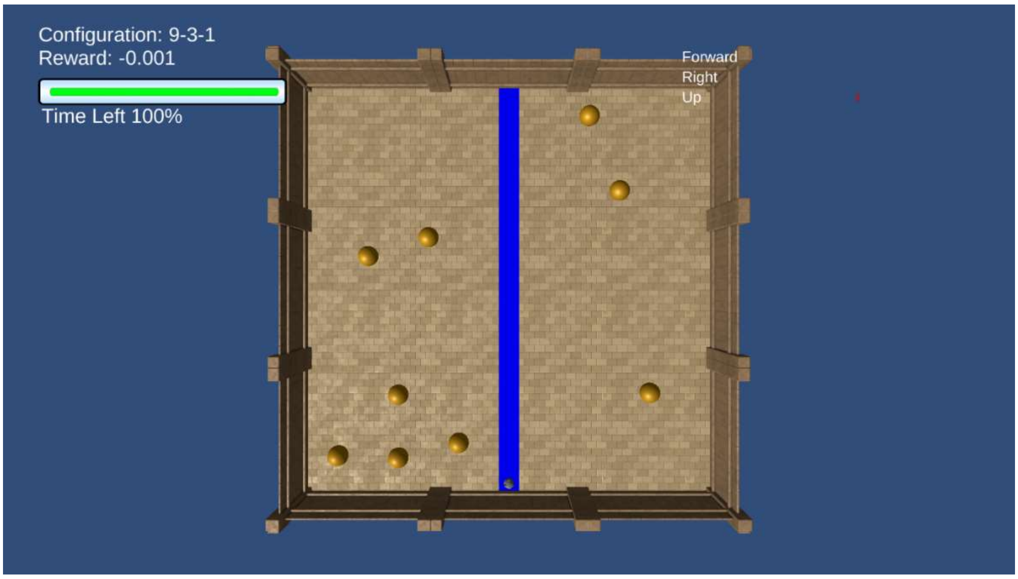

Pass criterion: forced choice numerosity task. To pass, the participant must select the left side and collect all six yellow 'fruit', within the time limit.

Test 10-16-1

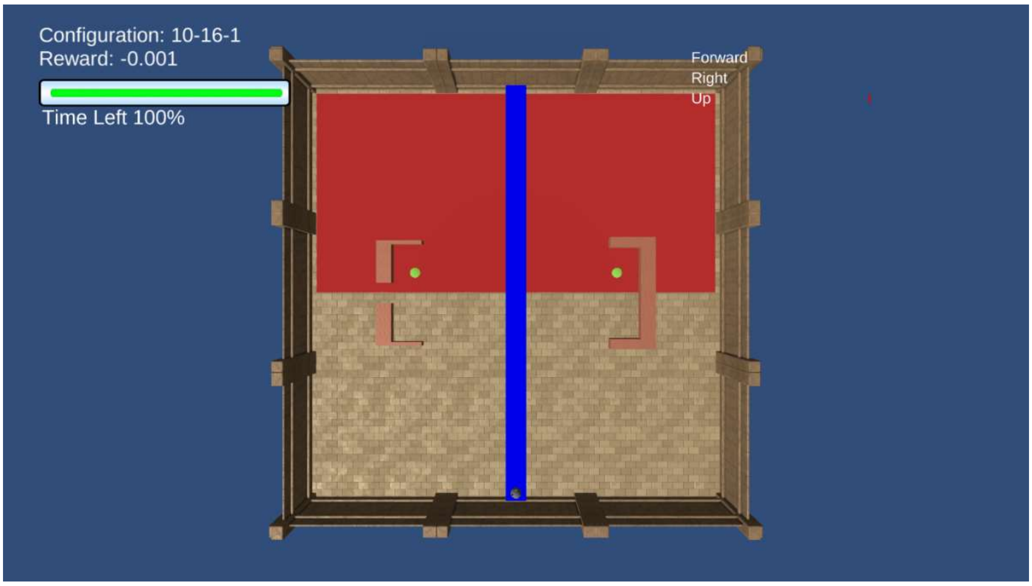

Explanation and pass criterion: This is an analogue of a forced-choice horizontal string-pulling task. It could also be interpreted as a hook type task. The objective is to navigate inside the crescent-shaped pushable object to use it to tug the green ‘fruit’ out of the ‘lava zone’. To pass, the participant must choose the right side, push the block to tug the ‘fruit’ out of the ‘lava zone’, and obtain the green ‘fruit’, within the time limit.

Test 10-21-1

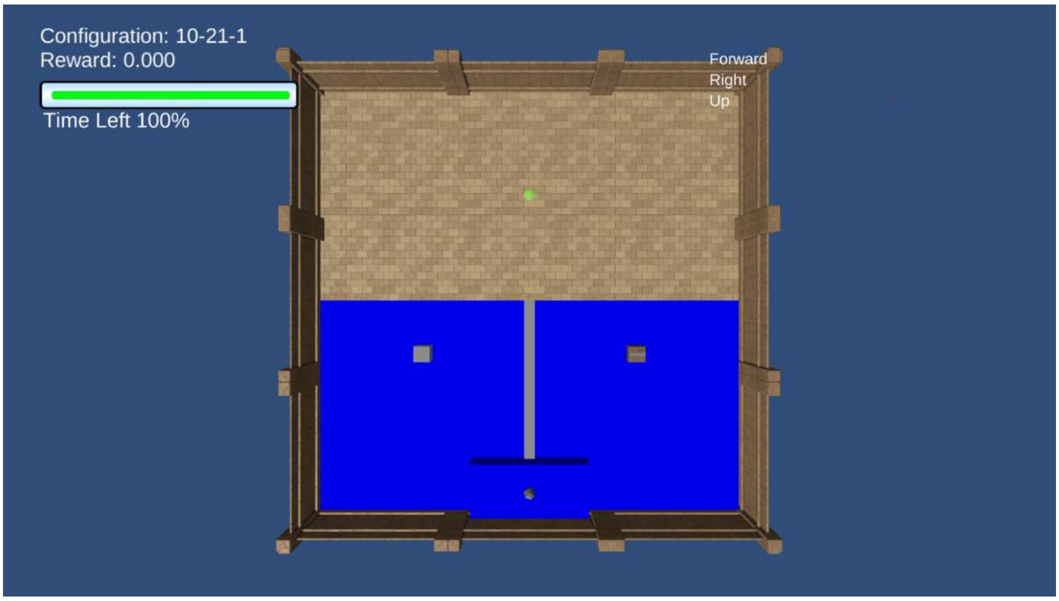

Explanation and pass criterion: This is a forced-choice tool use task. On the left there is an unmovable block, on the right there is a pushable ‘cardboard box’. The green ‘fruit’ is balanced on a pillar, out of reach, similarly to

5-15-1. To pass, the participant must select the right side, push the 'box' off the platform and towards the pillar to knock the green 'fruit' off, and then retrieve the green 'fruit' within the time limit.

Test 10-7-1

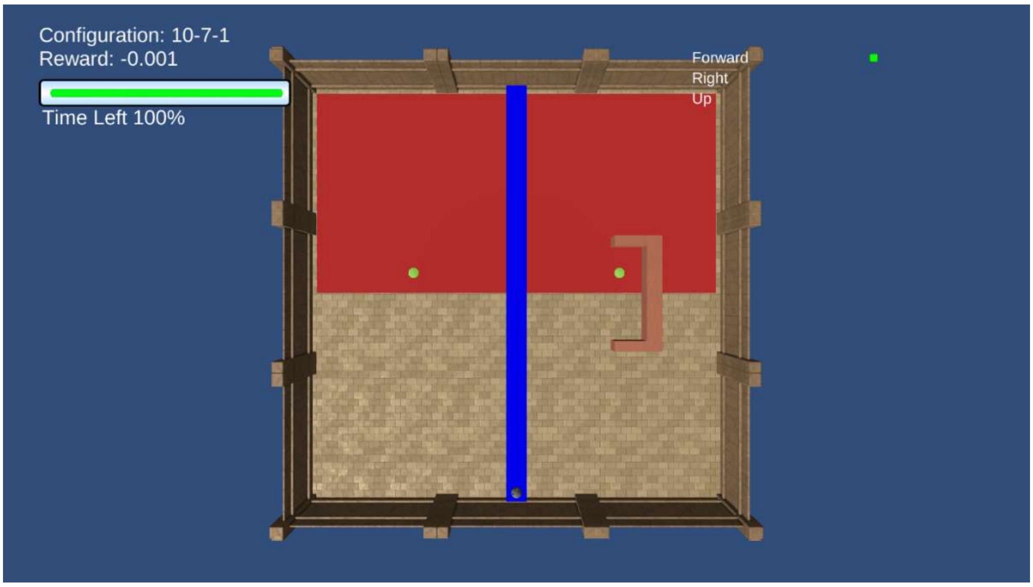

Explanation and pass criterion: As with 10-16-1, this requires the use of the pushable block as a tool, however, this task is simpler in that there is no distraction from the 'broken string'/'broken hook', i.e. the two L-shaped blocks in 10-16-1. To pass, the participant must select the right side, tug the green 'fruit' out of the 'lava zone', and retrieve it within the time limit.

Test 10-22-3

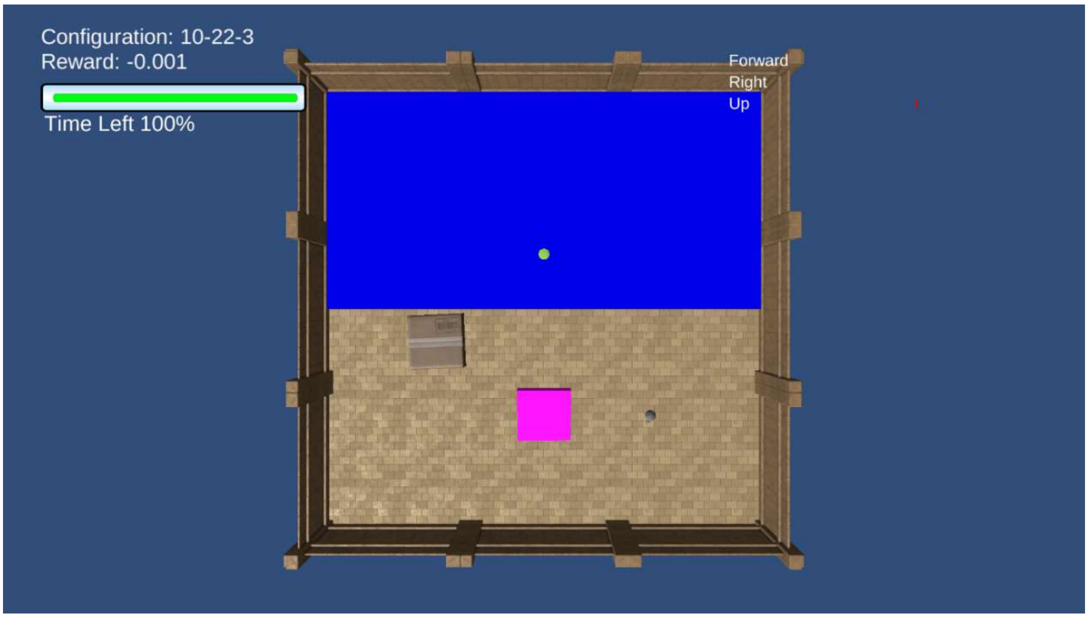

Explanation and pass criterion: This is an analogue of Köhler's (1917/1925) experiment with Sultan. It requires pushing the 'box' between the ramp and platform to create a bridge to retrieve the green 'fruit'. This is the pass criterion.
